# Supplementary material for: Visualizing stepwise evolution of carbon hybridization from sp3 to sp2 and to sp
Source: Nat Commun. 2025 Jan 15;16:690. doi: 10.1038/s41467-024-55719-4 (PMC11735776; doi:10.1038/s41467-024-55719-4)
Supplement: Supplementary file 1 — Supplementary Information [file 41467_2024_55719_MOESM1_ESM.pdf]

Supplementary Information

Visualizing stepwise evolution of carbon hybridization from *sp*^{3} to *sp*^{2} and to *sp*

Wei Xiong[1,‡], Guang Zhang[2,‡], De-Liang Bao[3,‡], Jianchen Lu[1,‡,*], Lei Gao[4], Yusen Li[2], Hui Zhang[1], Zilin Ruan[1], Zhenliang Hao[1], Hong-Jun Gao[5], Long Chen[2,6,*], Jinming Cai[1,7,*]

[1]Faculty of Materials Science and Engineering, Kunming University of Science and Technology; Kunming, 650093, PR China.

[2]Department of Chemistry and Tianjin Key Laboratory of Molecular Optoelectronic Science, Tianjin University; Tianjin, 300072, PR China.

[3]Department of Physics and Astronomy, Vanderbilt University; Nashville, TN, 37235, USA.

[4]Faculty of Science, Kunming University of Science and Technology; Kunming, 650093, PR China.

[5]Beijing National Center for Condensed Matter Physics and Institute of Physics, Chinese Academy of Sciences; Beijing, 100190, PR China.

[6]State Key Laboratory of Supramolecular Structure and Materials, College of Chemistry, Jilin University; Changchun, 130012, PR China.

[7]Southwest United Graduate School; Kunming, 650093, PR China.

‡These authors contributed equally: Wei Xiong, Guang Zhang, De-liang Bao, Jianchen Lu

*To whom correspondence should be addressed.

E-mail: jclu@kust.edu.cn, longchen@jlu.edu.cn, j.cai@kust.edu.cn.

1. Synthesis of molecular precursors

All chemicals and solvents were purchased from commercial sources and used as received except where noted. Reactions were all conducted under argon atmosphere. ^{1}H NMR and ^{13}C NMR spectra were recorded on Bruker AVANCE III HD 400 MHz spectrometer. The solvents for NMRs were $CDCl_3$ with the reference peak at 7.26 ppm (^{1}H) and $C_2D_2Cl_4$ with the reference peak at 5.91 ppm (^{1}H) and 74.2 ppm (^{13}C). MALDI-TOF mass spectra were recorded by a Bruker Autoflex MAX spectrometer with fullerene as the reference and dithranol as the matrix. The compound **M1**, **M2** were synthesized; **M3** and **DBP** was purchased from Leyan company.

M1: 1,6-di[2-(4-cyanomethylphenyl)ethynyl]pyrene

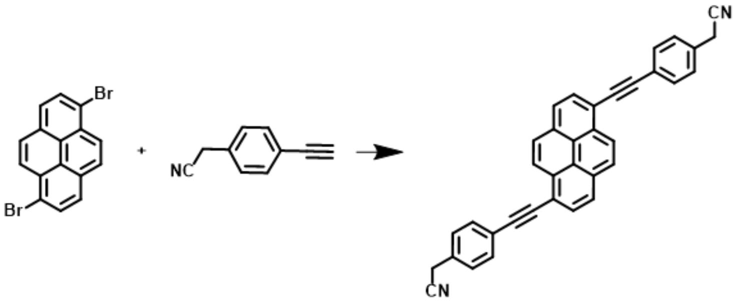

M1

In a Schlenk flask was added 1,6-dibromopyrene (0.170 g, 0.472 mmol), 4-ethynylphenylacetonitrile (0.186 g, 1.32 mmol), triphenylphosphine (12 mg, 0.047 mmol), CuI (4 mg, 0.024 mmol), $Pd(PPh_3)_2Cl_2$ (17 mg, 0.024 mmol), 5.1 mL of THF and 5.1 mL of triethylamine sequentially; degassed and refilled with argon 3 times; then heated the mixture at 80°C overnight. Then, cooled the mixture down to room temperature and added copious amount of water to precipitate the solid and filtered to get yellow-colored solid which was washed with methanol, ethyl acetate, and DCM sequentially; finally, 0.200 g of yellow-colored product was obtained after drying under reduced pressure (76.9%). ^{1}H NMR (400 MHz, $C_2D_2Cl_4$, 300 K) δ 8.60 (d, 6H), 8.19 (m, 6H), 7.68 (d, 4H), 7.36 (d, 4H), 3.77 (s, 4H); ^{13}C NMR (100

MHz, $C_2D_2Cl_4$, 300 K) δ 132.29, 131.75, 131.01, 130.03, 128.18, 128.02, 126.10, 125.23, 123.84, 123.07, 117.96, 117.75, 94.69, 89.19, 67.79, 25.53, 23.54 (Supplementary Fig. 24). MALDI-TOF: calculated for $C_{36}H_{20}N_2$: 480.16(100%), found: 479.90 [M^+] (Supplementary Fig. 25).

M2: 1,4-di[2-(4-cyanomethylphenyl)ethynyl]benzene

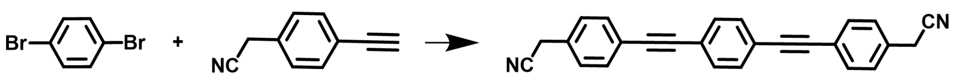

M2

In a Schlenk flask was added 1,4-dibromobenzene (80 mg, 0.339 mmol), 4-ethynylphenylacetonitrile (0.134 g, 0.949 mmol), triphenylphosphine (9 mg, 0.034 mmol), CuI (3 mg, 0.017 mmol), $Pd(PPh_3)_2Cl_2$ (12 mg, 0.017 mmol), 6.0 mL of DMF and 2.0 mL of triethylamine sequentially; degassed and refilled with argon 3 times; then heated the mixture at 80 ^oC for 2 days. Then, cooled the mixture down to room temperature and added copious amount of water to precipitate the solid and filtered to get yellow-colored solid which was washed with methanol, ethyl acetate, and DCM sequentially; finally, 79 mg of beige-colored product was obtained after drying under reduced pressure (66%). ^1H NMR(400 MHz, $CDCl_3$, 300 K) δ 7.54 (d, 4H), 7.52 (s, 4H), 7.35 (d, 4H), 3.79 (s, 4H); ^{13}C NMR (100 MHz, $C_2D_2Cl_4$, 300 K) δ 133.53, 132.67, 131.97, 130.44, 128.36, 123.14, 118.17, 90.89, 90.24, 23.93. (Supplementary Fig. 26). MALDI-TOF: calculated for $C_{26}H_{16}N_2$: 356.13 (100%), found: 355.70 [M^+] (Supplementary Fig. 27).

M3:2,2'-(5'-(4-(Cyanomethyl)phenyl)-[1,1':3',1''-terphenyl]-4,4''-diyl)diacetonitrile

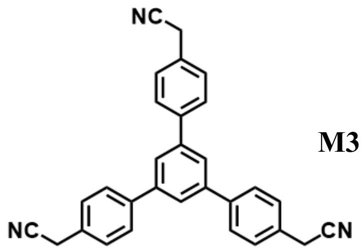

M3

DBP: 4,4'-diethyl-1,1'-biphenyl

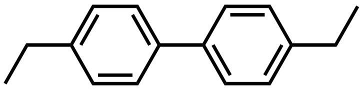

DBP

2. Experimental details

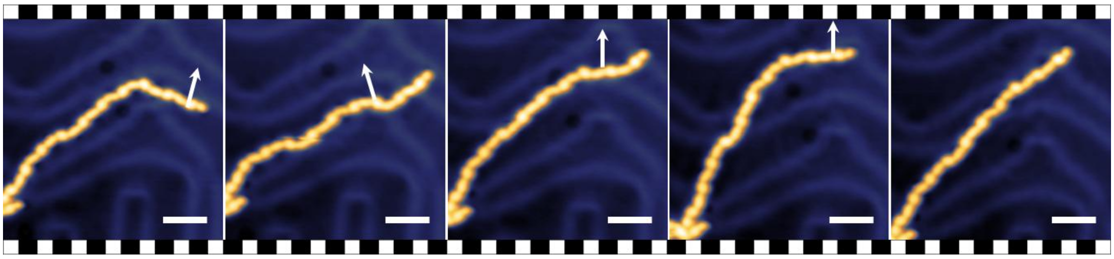

Supplementary Fig. 1 | A series of lateral tip manipulation experiments performed on a polymer 1A. The white arrows indicate the direction of the lateral tip manipulations (10 mV, 1 nA). Scanning parameters: (400 mV, 80 pA) for all STM images. All scale bars, 5 nm.

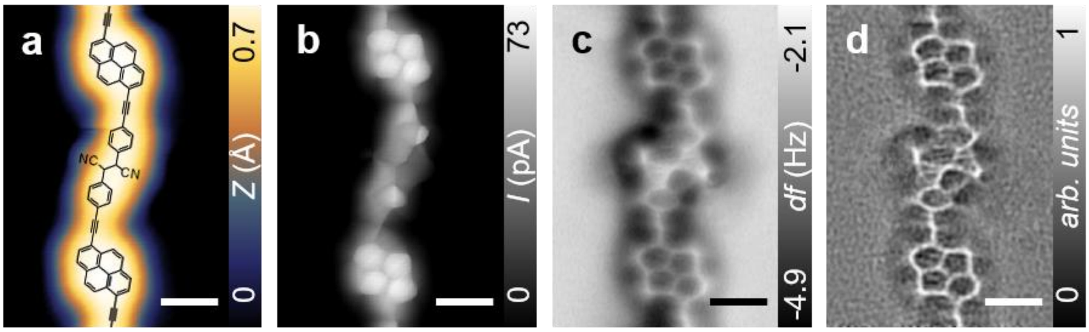

Supplementary Fig. 2 | High resolution characterization of polymer 1A. **a-d**, STM image (200 mV, 100 pA) (**a**), BR-STM image (2 mV, Δz = 170 pm) (**b**), nc-AFM image (2 mV, Δz = 200 pm) (**c**) and nc-AFM Laplace-filtered image (**d**) of a polymer **1A** comprising *sp*^{3}-hybridized linkage *L*(C–C). Scale bars, 0.6 nm (**a-d**).

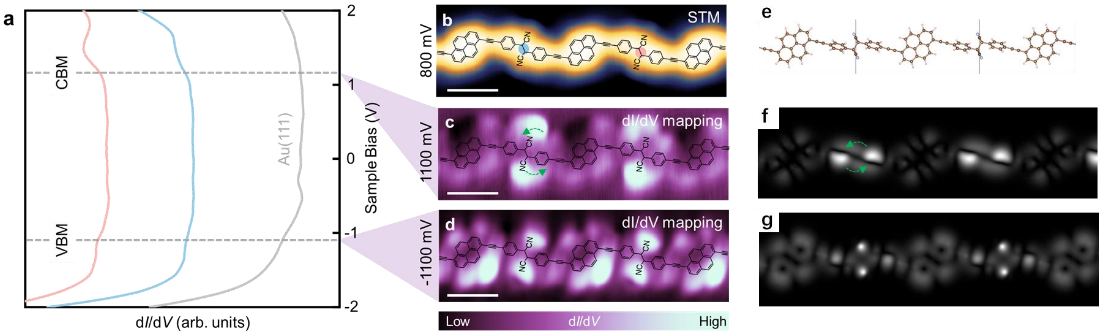

Supplementary Fig. 3 | Electronic structures of the polymer 1A on Au(111). a, d*I*/d*V* spectra recorded on two adjacent linkages of the polymer **1A**. The red and blue curves are from the corresponding colored spots in (**b**). The grey curve is taken on a bare Au(111) surface. **b**, STM image (400 mV, 20 pA) of the polymer **1A** superimposed by the corresponding chemical structures. The blue and pink dots represent the positions of spectra acquisition. **c-d**, Constant-current d*I*/d*V* maps conducted at potential energies of -1100 mV (**c**) and 1100 mV (**d**), respectively. d*I*/d*V* maps are superimposed by chemical structures to enhance the visualization of frontier molecular orbitals distribution. Scale bars, 1 nm (**b-d**). **e**, Top view of the atomic model of freestanding polymer **1A** chain. **f-g**, Simulated dI/dV mappings of polymer **1A**.

We highlight the similarity between the experiment and the simulation, particularly the bright protrusions at the –CN functional groups, which are upwards from the substrate. Notably, the electron density around –CN couples with the adjacent phenyl rings, forming an arc-like morphology on both sides, a feature also captured in simulations (dashed green arrows in Supplementary Fig. 3c and f). DFT-calculated dI/dV mapping simulation acquired at negative bias of -2 V present that two upward-going –CN functional groups appear as isolated bright dots, and the pyrene group show comparable brightness, consistent with the experimental dI/dV mapping in Supplementary Fig. 3d. It is important to note that due to the doping

effect of the substrate and future planeness of molecules on substrate, the bias voltages in simulations are not quantitative comparable to those in the experiments.

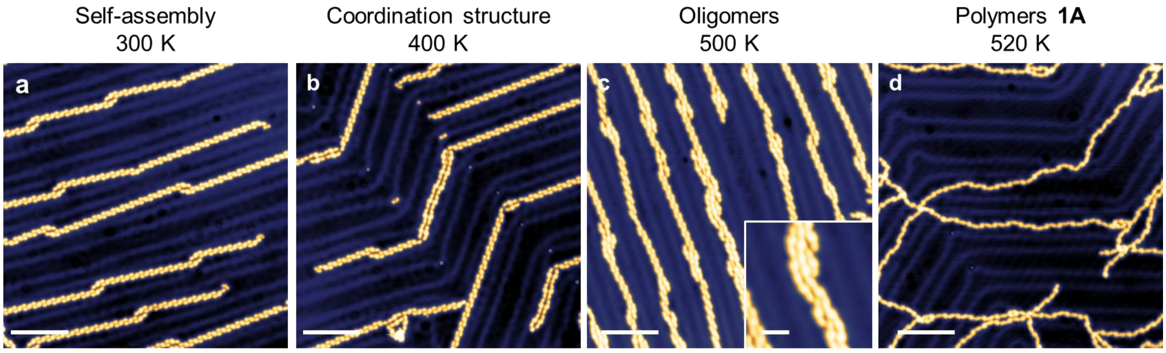

Supplementary Fig. 4 | Stepwise annealing M1 at different temperatures on Au(111). a, STM image (500 mV, 50 pA) showing the self-assembly of precursor **M1** on Au(111). **b**, STM image (300 mV, 20 pA) presenting the appearance of coordinated structure obtained after annealing at 400 K. **c**, STM image (400 mV, 40 pA) showing the appearance of some polymerized oligomers after annealing at 500 K. The inset: zoom-in STM topographic image (1000 mV, 20 pA) of the oligomers. **d**, STM image (500 mV, 50 pA) showing longer polymers **1A** *via* annealing at 520 K. Scale bar, 10 nm (**a-d**), 2 nm (inset image in **c**).

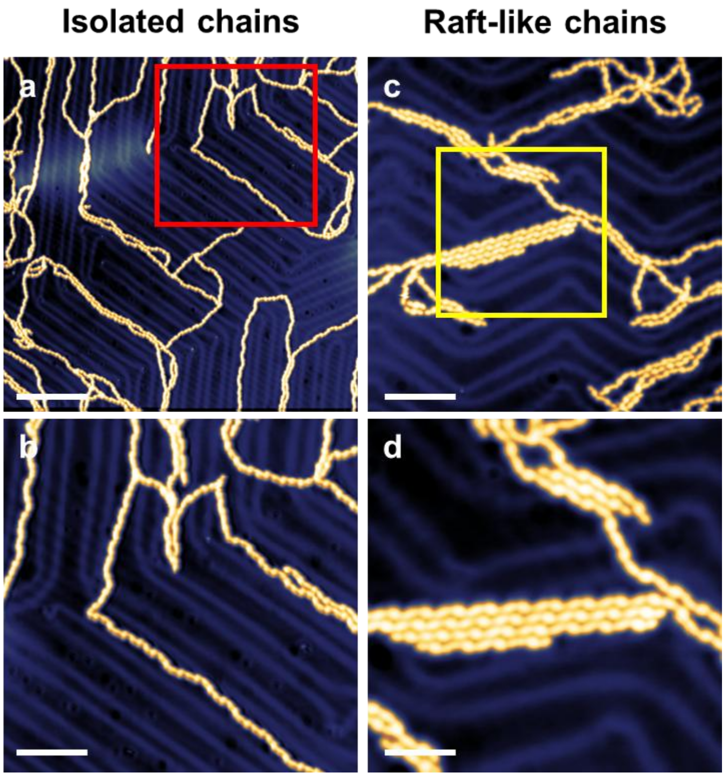

Supplementary Fig. 5 | Isolated and raft-like polymers 1A on Au(111). **a**, Large-scale STM image (500 mV, 50 pA) presenting isolated polymers **1A**. **b**, Zoom-in STM image (400 mV, 20 pA) extracted from (**a**) marked by red rectangle area. **c**, Large-scale STM image (500 mV, 20 pA) presenting raft-like polymers **1A**. **d**, Zoom-in STM image (500 mV, 20 pA) extracted from (**c**) marked by yellow rectangle area. The cause for such raft-like polymers **1A** is mainly attributed to the intermolecular weak interactions between –CN groups and aromatic skeletons (CN···H). Scale bars, 16 nm (**a**), 10 nm (**b**), 8 nm (**c**), 5 nm (**d**).

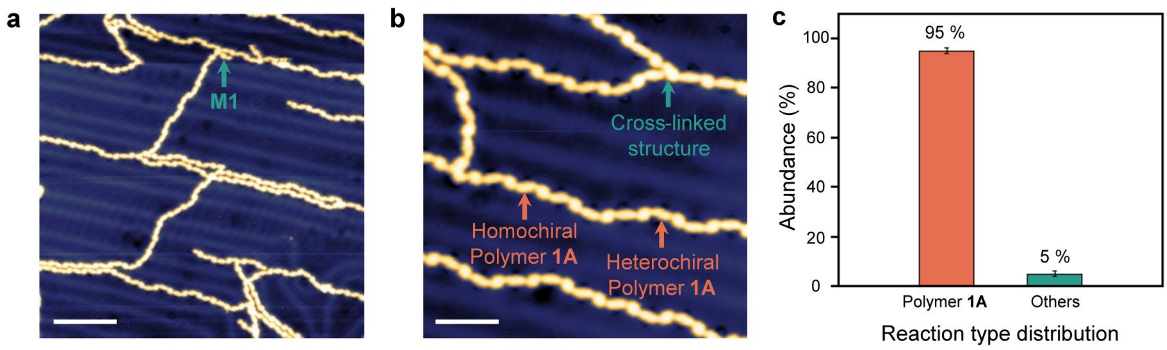

Supplementary Fig. 6 | Statistical analysis for the products of M1 after annealing to 520 K on Au(111) surface. a, Large-scale STM image (200 mV, 100 pA) showing the **M1**, polymers **1A** and other cross-linked structures on Au(111) surface. **b**, High-resolution STM image (100 mV, 250 pA) showing that the polymers **1A** display homochiral and heterochiral connected modes. The cross-linked structure is attributed to the cyclization between –CN groups with pyrene skeletons. **c**, Statistical analysis (counting 360 chains in total) for the polymer **1A** and other structures on Au(111) after annealing to 520 K. Scale bar, 10 nm (**a**), 5 nm (**b**).

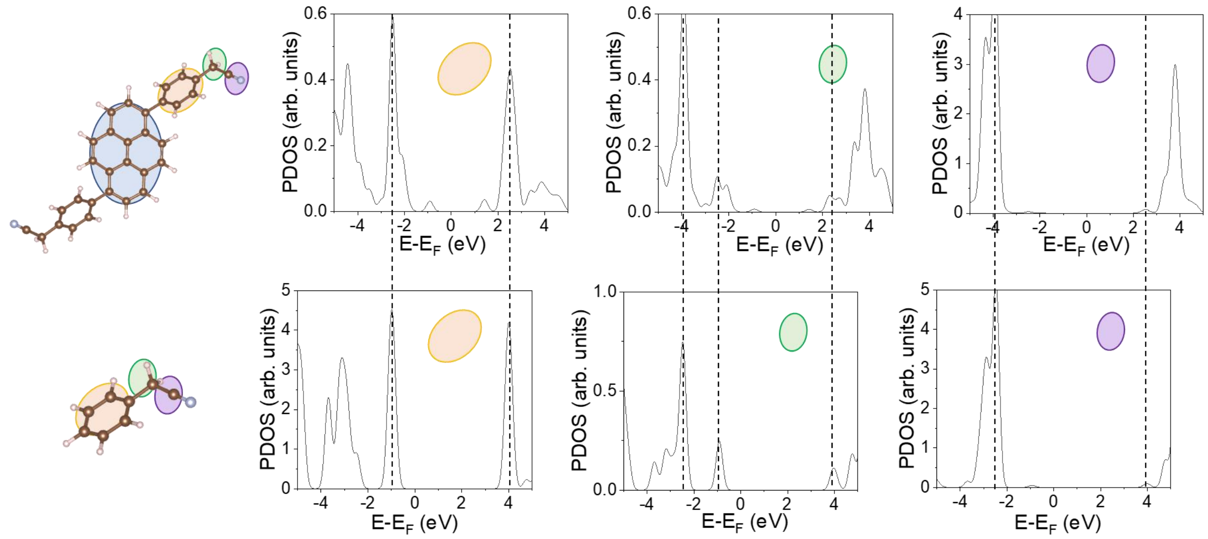

Supplementary Fig. 7 | The projected density of states (PDOS) on both M1 molecule and phenylacetonitrile molecules. The PDOS on each functional group, respectively, show identical features between two molecules, expect for a constant shift of the fermi level, which is understandable for in bigger molecule the involved pyrene functional group will modify the fermi level of the molecule. The influence of the electron-withdraw group –CN on dehydrogenation process has been effectively simulated. DFT simulation functional: PBE+D3.

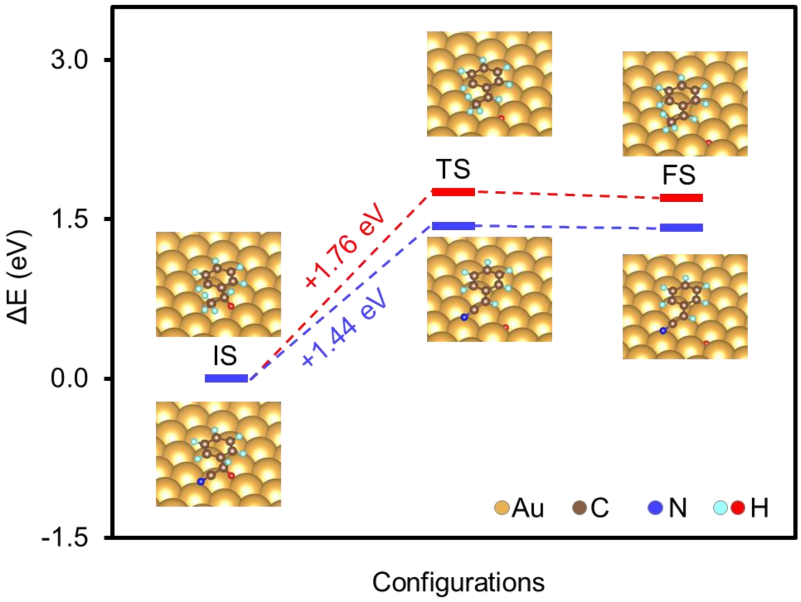

Supplementary Fig. 8 | DFT-calculated energy barriers of dehydrogenation in prototype molecules of ethylbenzene (higher) and phenylacetonitrile (lower). DFT-calculated energy barriers of dehydrogenation in prototype molecules of ethylbenzene (higher) and phenylacetonitrile (lower). The red atoms in inserts highlight the released H atoms. DFT simulation functional: PBE+D3.

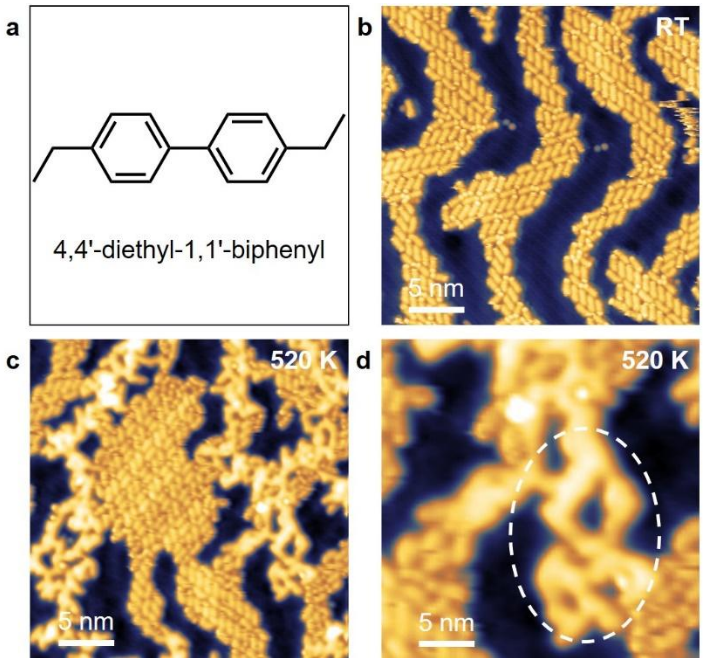

Supplementary Fig. 9 | A control experiment of DBP molecule on Au(111) substrate. **a,** Chemical structure of **DBP** molecule. **b,** STM image showing the self-assembled **DBP** molecules on Au(111) surface (200 mV, 120 pA). **c-d,** Overview and magnified STM images (200 mV, 50 pA) showing the edge-fused by-products after annealing **DBP** molecules to 520 K.

Supplementary Fig. 9a presents the chemical structure of **DBP** molecule functionalized with two $–CH_2–CH_3$ groups Initially, upon the deposition of **DBP** molecules onto Au(111) surface held at room temperature, island-like self-assembly structures are formed (as shown in Supplementary Fig. 9b). Subsequently, annealing the sample to 520 K triggers polymerization reactions among some **DBP** molecules (Supplementary Fig. 9c). From the magnified image, it can be observed that the regions marked by white dashed ellipses are all edge-fused products, indicating heterogeneous reaction sites, i.e., the terminal methyl groups cannot activate adjacent methylene groups (Supplementary Fig. 9d). Moreover, Chi et. Al. did not observe the dehydrogenative homocoupling between methylene groups during the synthesis of hexabenzocoronene-cored graphdiyne nanosheets using hexa(4-ethylphenyl)benzene (**HPB−Et**) molecules[1]. Based on the comparative experimental results between **M1** and **DBP** molecules, we believe that the cyano group plays a crucial role in activating the methylene group.

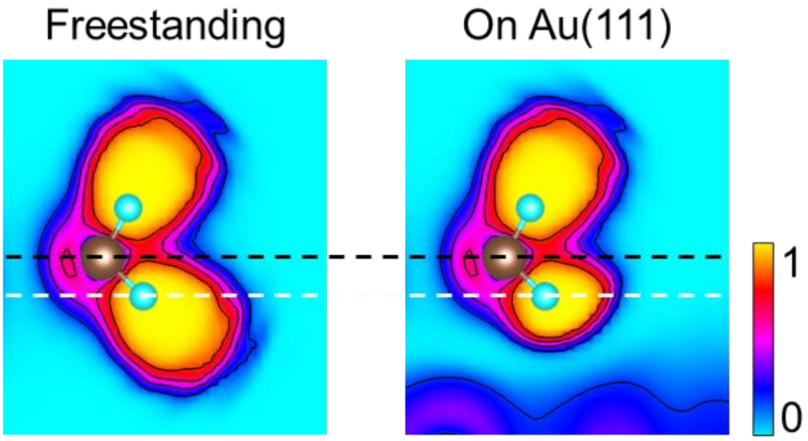

Supplementary Fig. 10 | Section-view electron localization function in the plane through –CH_2– atoms of phenylacetonitrile for freestanding (left) and on Au(111) (right). Contour lines interval: 0.2. Black and white dashed lines mark the relative positions of C and H atoms.

The effect of the substrate towards the selectivity of C–H activation can be validated through the electron localization function (ELF), which ranges from 0 to 1 and quantifies the degree of electron localization. The calculated ELF of –CH_2– in freestanding case is symmetric, *i.e.*, the two C–H bonds are equivalent. On the other hand, the ELF of –CH_2– on the surface, however, exhibits noticeable asymmetry, indicating a significant weakening of the C–H bond in proximity to the Au(111) surface. DFT simulation functional: PBE+D3.

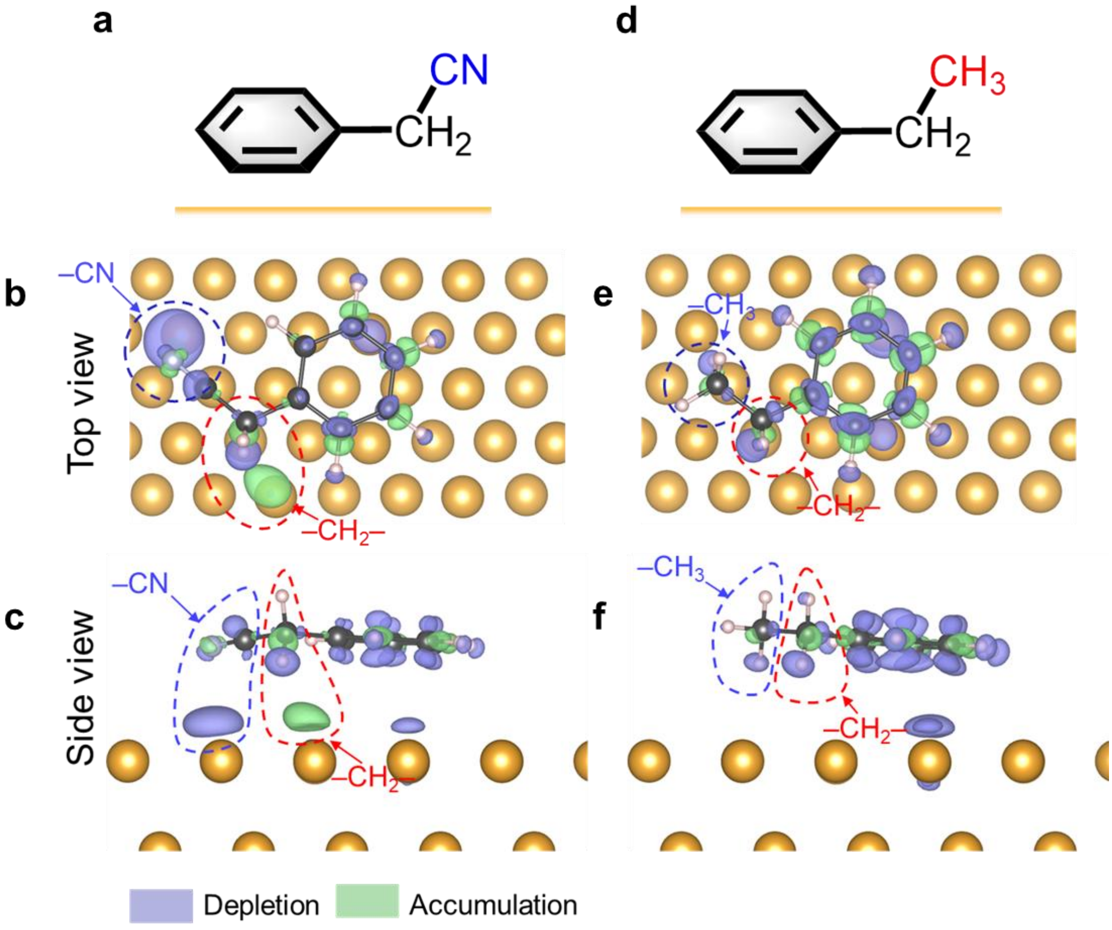

Supplementary Fig. 11 | DFT-calculated charge density differences of phenylacetonitrile and ethylbenzene on Au surface. a, Schematic of phenylacetonitrile on Au. **b-c**, Top (**b**) and side (**c**) views of charge density difference between phenylacetonitrile molecule and Au substrate, like the main text Fig. 2j. **d**, Schematic of ethylbenzene on Au. **e-f**, Top (**e**) and side (**f**) views of charge density difference between ethylbenzene molecule and Au substrate. In (**b-c**) and (**e-f**), purple isosurface presents electron depletion, while green isosurface presents electron accumulation. Isosurface value: 6.7×10^{-3} e/Å^3.

We emphasize that the charge density distributions for the two molecules exhibit distinct differences. As discussed in the main text, for phenylacetonitrile, there is a region of charge depletion between the –CN group and the substrate due to the electron-withdrawing nature of the –CN functional group (highlighted by blue dashed curves in panels Supplementary Fig. 11b-c). This leads to charge accumulation between the $–CH_2–$ group and the substrate, which

facilitates dehydrogenation (highlighted by red dashed curves in panels Supplementary Fig. 11b-c).

In contrast, for ethylbenzene on Au, there is no significant charge redistribution between the functional groups and the substrate, indicating no facilitation of dehydrogenation (highlighted by blue and red curves in panels Supplementary Fig. 11e-f).

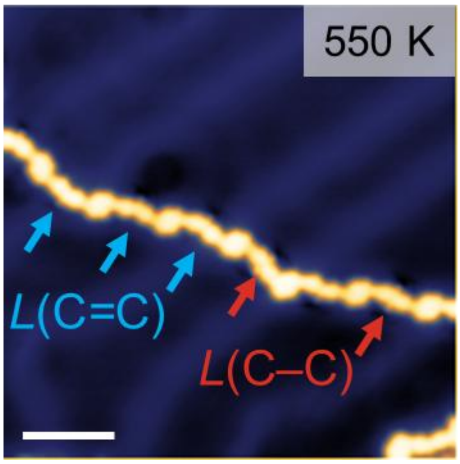

Supplementary Fig. 12 | Polymers with *L*(C–C) and *L*(C=C) prepared on Au(111) surface *via* annealing M1 at 550 K. STM image (500 mV, 20 pA) showing a polymer consisting of *L*(C–C) and *L*(C=C) linkages marked by red and blue arrows respectively. Scale bars, 3 nm.

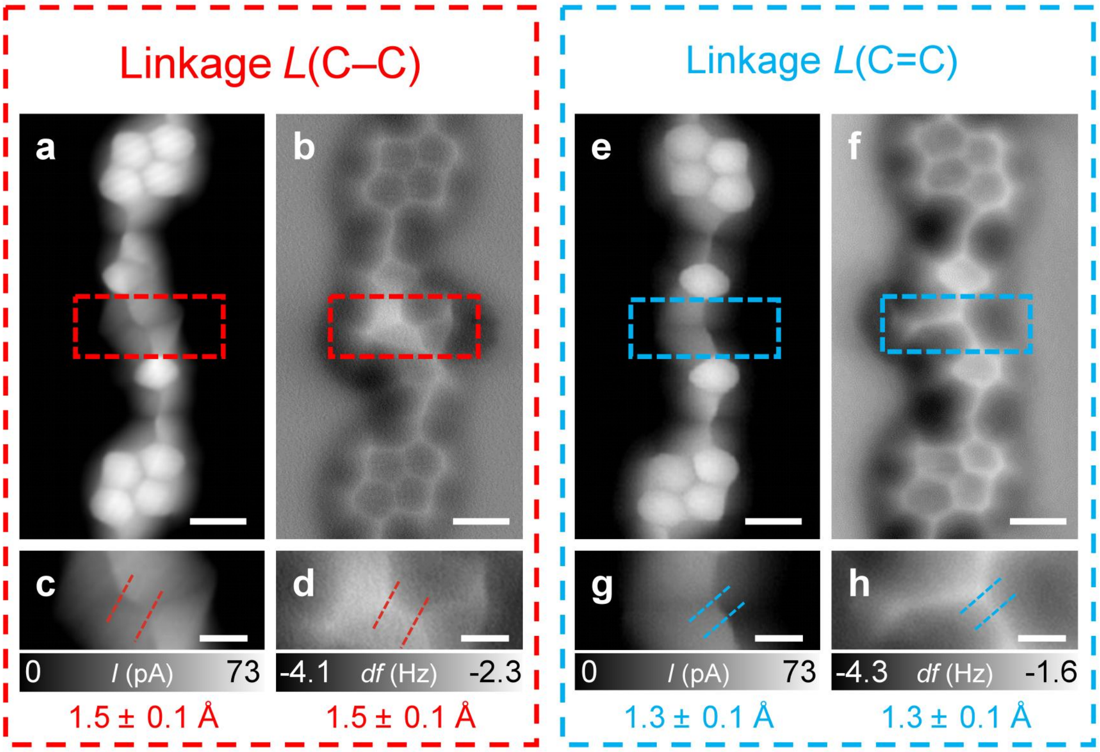

Supplementary Fig. 13 | Comparison between the length of newly-formed carbon bonds within *L*(C–C) and *L*(C=C) linkages. a-b, BR-STM (2 mV, Δz = 150 pm) (**a**) and nc-AFM image (2 mV, Δz = 220 pm) (**b**) of the polymer **1A** with the linkage *L*(C–C). **c-d**, Close-up BR-STM and nc-AFM image of linkage *L*(C–C) derived from the area indicated by red boxes in (**a**) and (**b**). The length of the newly-formed carbon single bond is measured to be 1.5 ± 0.1 Å. **e-f**, BR-STM (2 mV, Δz = 160 pm) (**e**) and nc-AFM image (2 mV, Δz = 190 pm) (**f**) of the polymer **1B** with the linkage *L*(C=C). **g-h**, Close-up BR-STM and nc-AFM image of linkage *L*(C=C) derived from the area indicated by blue boxes in (**e**) and (**f**). The length of the newly-formed carbon double bond is measured to be 1.3 ± 0.1 Å. Scale bars, 0.4 nm (**a-b**, **e-f**), 0.2 nm (**c-d**, **g-h**).

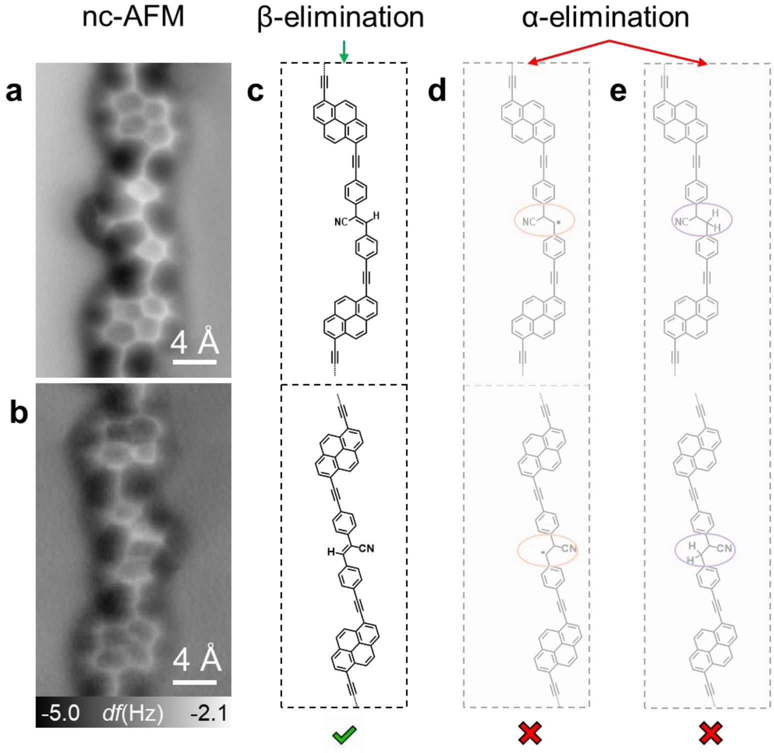

Supplementary Fig. 14 | Identification of elimination reaction types. a-b, nc-AFM images (2 mV, Δz = 220 pm) showing the homochiral and heterochiral polymers **1B** with the linkage *L*(C=C). **c**, Chemical structures of polymer **1B** induced by β-elimination reaction. **d-e**, Two potential chemical structures of polymer **1B** induced by α-elimination reaction.

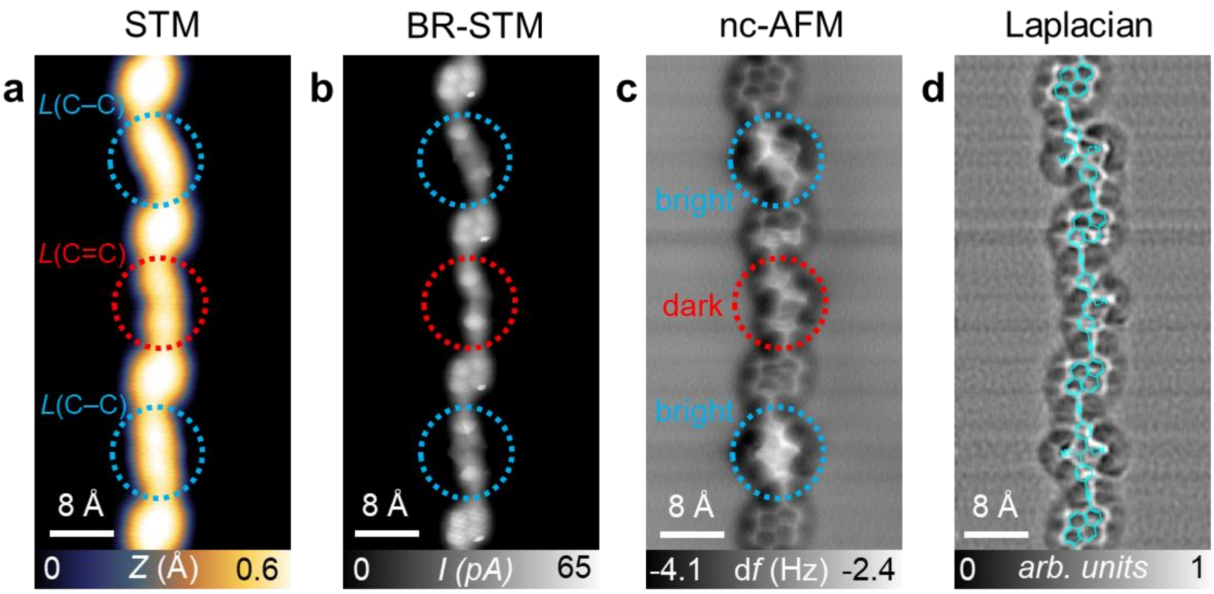

Supplementary Fig. 15 | High-resolution characterization of the intermediated polymer with linkages *L*(C–C) and *L*(C=C). a-d, High-resolution STM (200 mV, 100 pA) (**a**), BR-STM (2 mV, Δz = 170 pm) (**b**), nc-AFM (2 mV, Δz = 200 pm) (**c**) and Laplace-filtered images (**d**) of the polymer with both linkage *L*(C–C) and *L*(C=C).

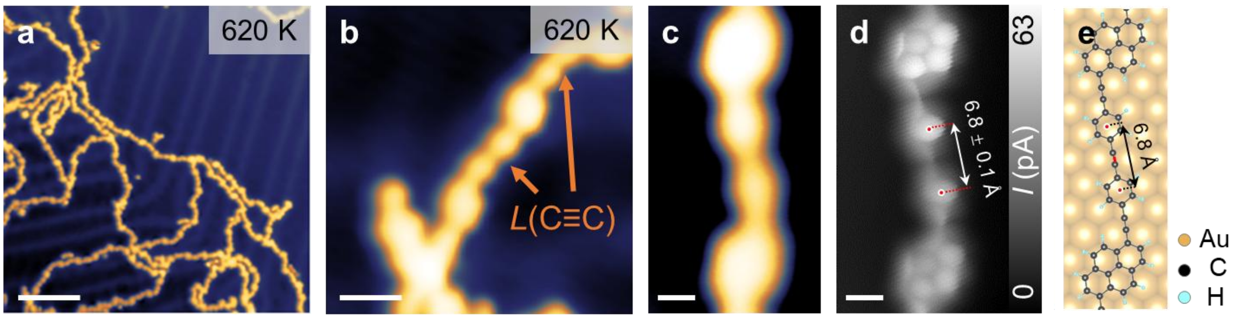

Supplementary Fig. 16 | Polymer 1C prepared on Au(111) surface *via* annealing M1 at 620 K. a, Overview STM image (500 mV, 20 pA) of the polymers after annealing to 620 K. **b**, Close-up STM image (100 mV, *I* = 50 pA) of a polymer **1C** with the linkages *L*(C≡C). **c**-**d**, High-resolution STM (100 mV, 150 pA) (**c**) and BR-STM image (2 mV, Δz = 170 pm) (**d**) of polymer **1C**. **e**, DFT-optimized model of a polymer **1C**. The experimental distance (**d**) between adjacent phenyl rings shows excellent accordance with that of calculated result (**e**). Scale bars, 10 nm (**a**), 1 nm (**b**), 0.4 nm (**c**-**d**).

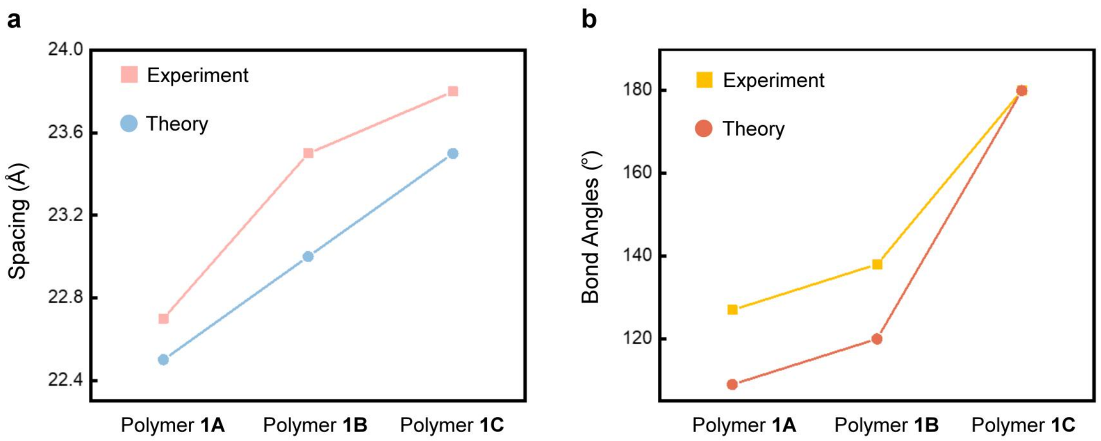

Supplementary Fig. 17 | The variation trend of intramolecular spacing and carbon bond angle of three polymers 1A, 1B, and 1C along with the transition of carbon hybridization. a-b, A gradual increase of the intramolecular spacing and carbon bond angle within three polymers along with the transitioning process of carbon hybridization from sp^3 to sp^2 and to sp.

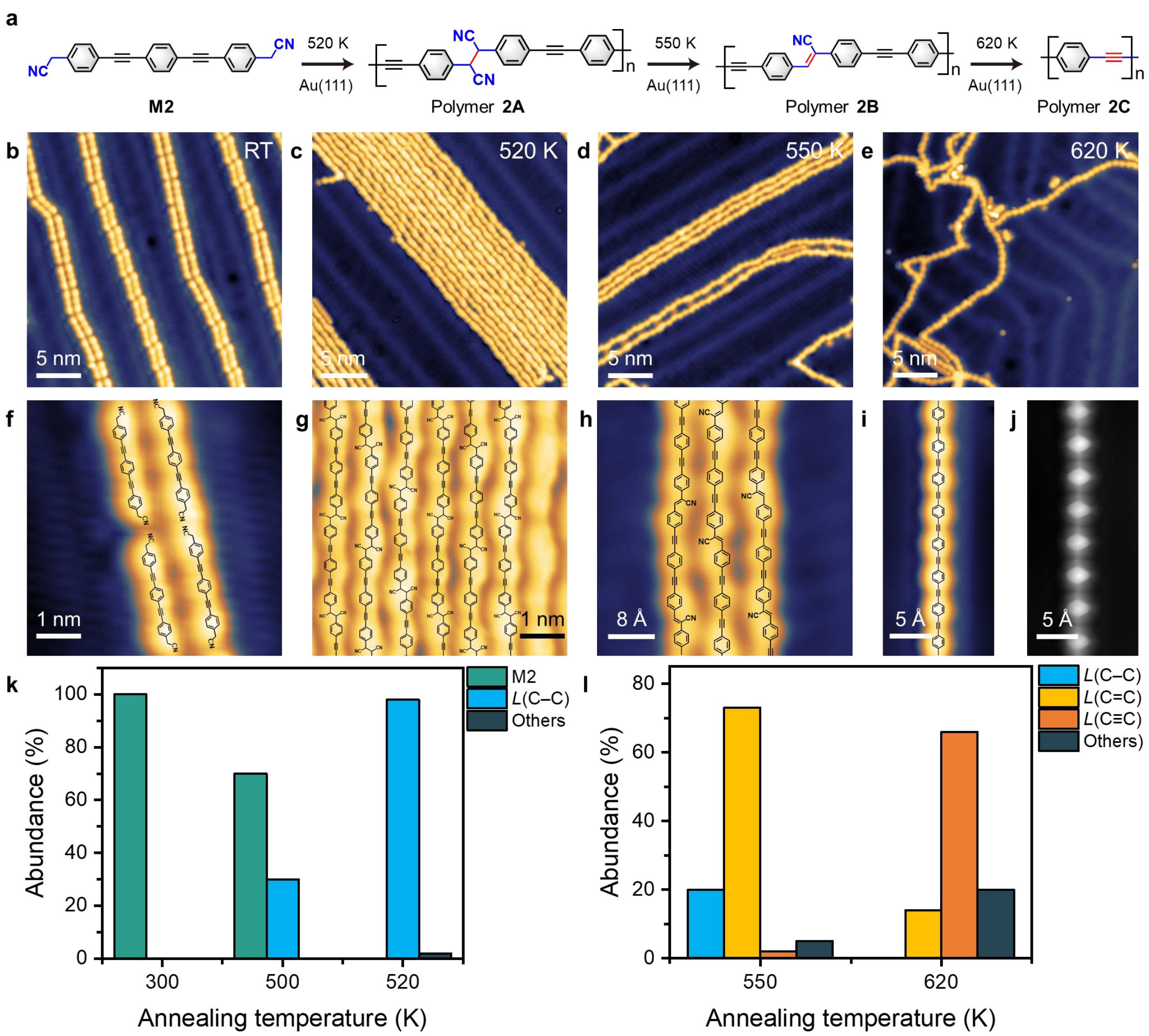

Supplementary Fig. 18 | Annealing M2 to different temperatures on Au(111) surface. **a**, Schematic representation showing the synthesis of polymers **2A**, **2B** and **2C**. **b-e**, Overview STM images (200 mV, 120 pA) showing the self-assembled **M2** molecules (**b**), polymer **2A** (**c**), **2B** (**d**) and **2C** (**e**) on Au(111) surface after annealing the sample to RT, 520 K, 550 K and 620 K for 20 mins, respectively. **f-h**, Magnified STM images (200 mV, 250 pA) showing the structures corresponding to (**b-e**) (200 mV, 250 pA). **i-j**, STM (200 mV, 250 pA) and BR-STM image (2 mV, Δz = 200 pm) of polymer **2C**. **k**, Yield statistics for the products resulting from the dehydrogenation reaction of **M2** molecules after annealing at 500 K and 520 K for 20 mins. **l**, Yield statistics for the products obtained from the two-step elimination reactions of **M2** molecules after annealing to 550 K and 620 K for 20 mins.

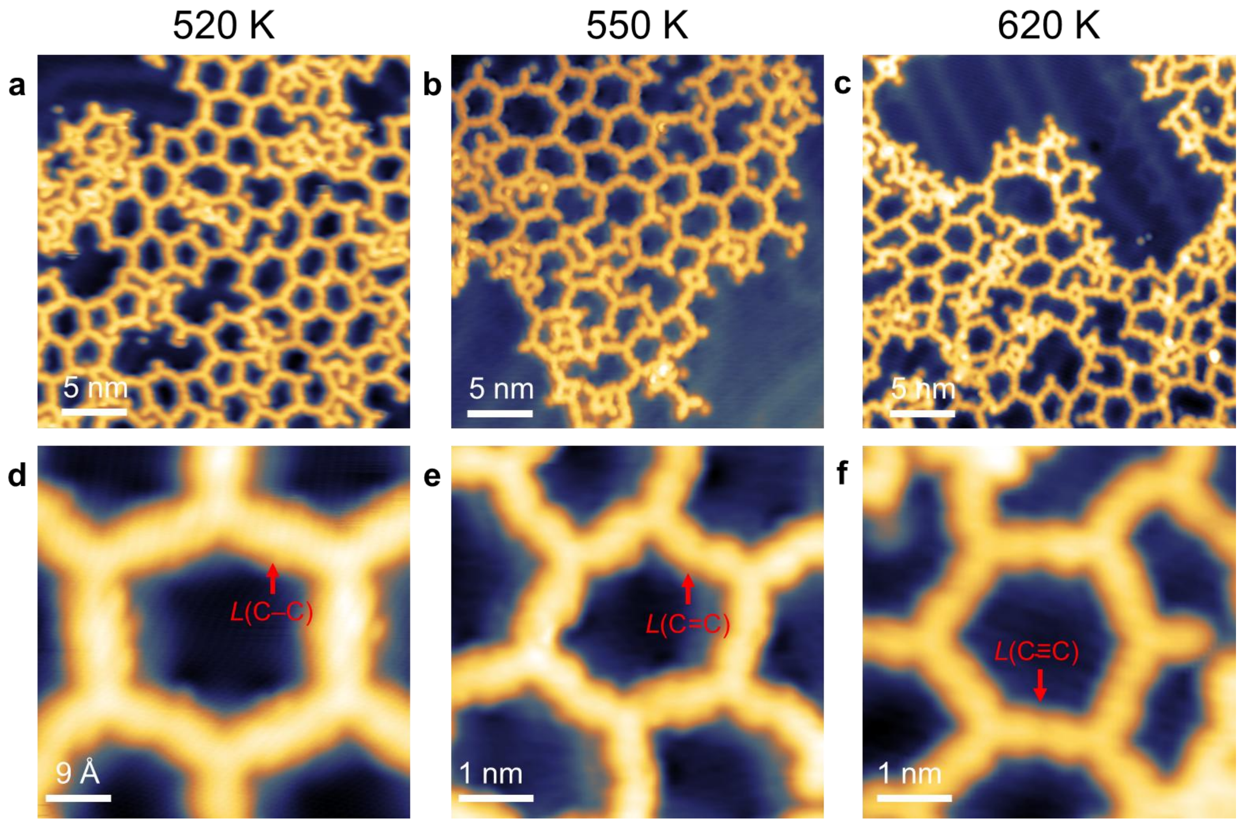

Supplementary Fig. 19 | The transition of carbon hybridization within 2D COFs fabricated *via* annealing M3 to different temperatures on Au(111). a-c, Large-scale STM images (200 mV, 150 pA) showing the COF structures fabricated by annealing **M3** to 520 K (**a**), 550 K (**b**), and 620 K (**c**) for 20 mins on Au(111) surface, respectively. **d-f**, Magnified STM image (400 mV, 150 pA) derived from (**a-c**).

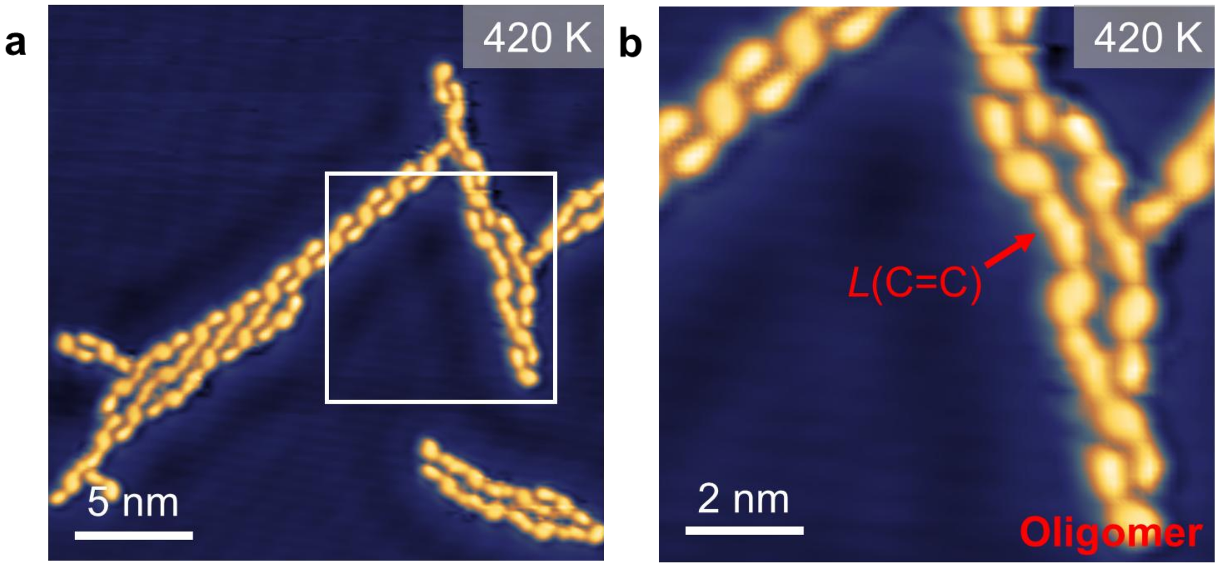

Supplementary Fig. 20 | The formation of oligomers with the linkage *L*(C=C) on Ag(111) surface *via* annealing M1 to 420 K for 20 mins. a-b, Overview and magnified STM images (200 mV, 100 pA) showing a coexistence of self-assembled **M1** molecules and initial polymeric oligomers with the linkage *L*(C=C) on Ag(111) surface.

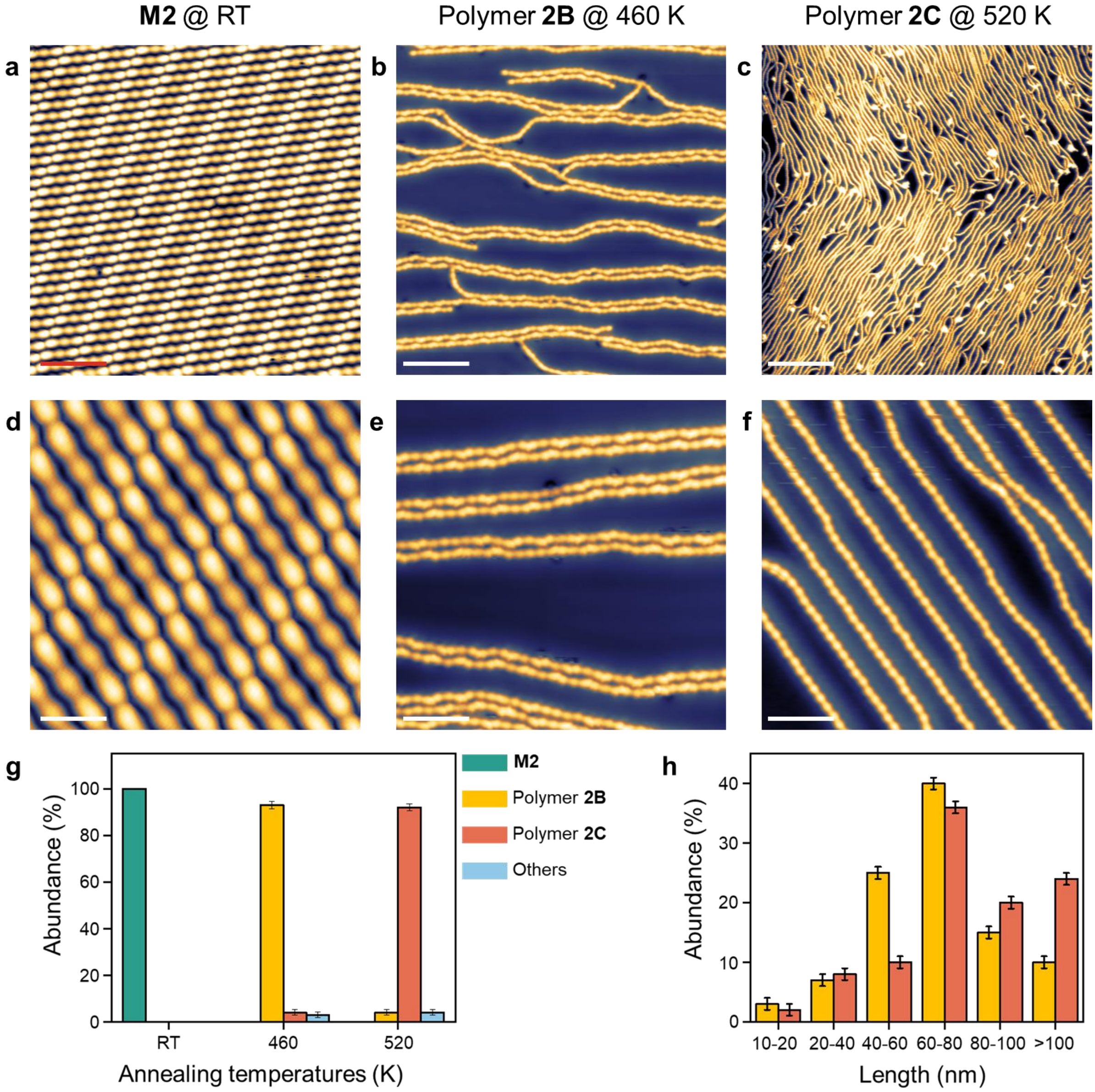

Supplementary Fig. 21 | Stepwise annealing M2 to different temperatures on Ag(110). a-c, Large-scale STM images (150 mV, 200 pA) of **M2** (**a**), polymer **2B** (**b**), and polymer **2C** (**c**) obtained *via* annealing to RT, 460 K, and 520 K, respectively. **d-f**, Close-up STM image (100 mV, 250 pA) corresponding to (**a-c**). **g**, Yield distribution statistics of **M2**, polymer **2B** and **2C** obtained *via* annealing to RT, 460 K, and 520 K respectively. **h**, Length distribution statistics of polymer **2B** and **2C**. Scale bar: 4 nm (**a**), 10 nm (**b**), 20 nm (**c**), 1 nm (**d**), 3 nm (**e**), 4 nm (**e-f**).

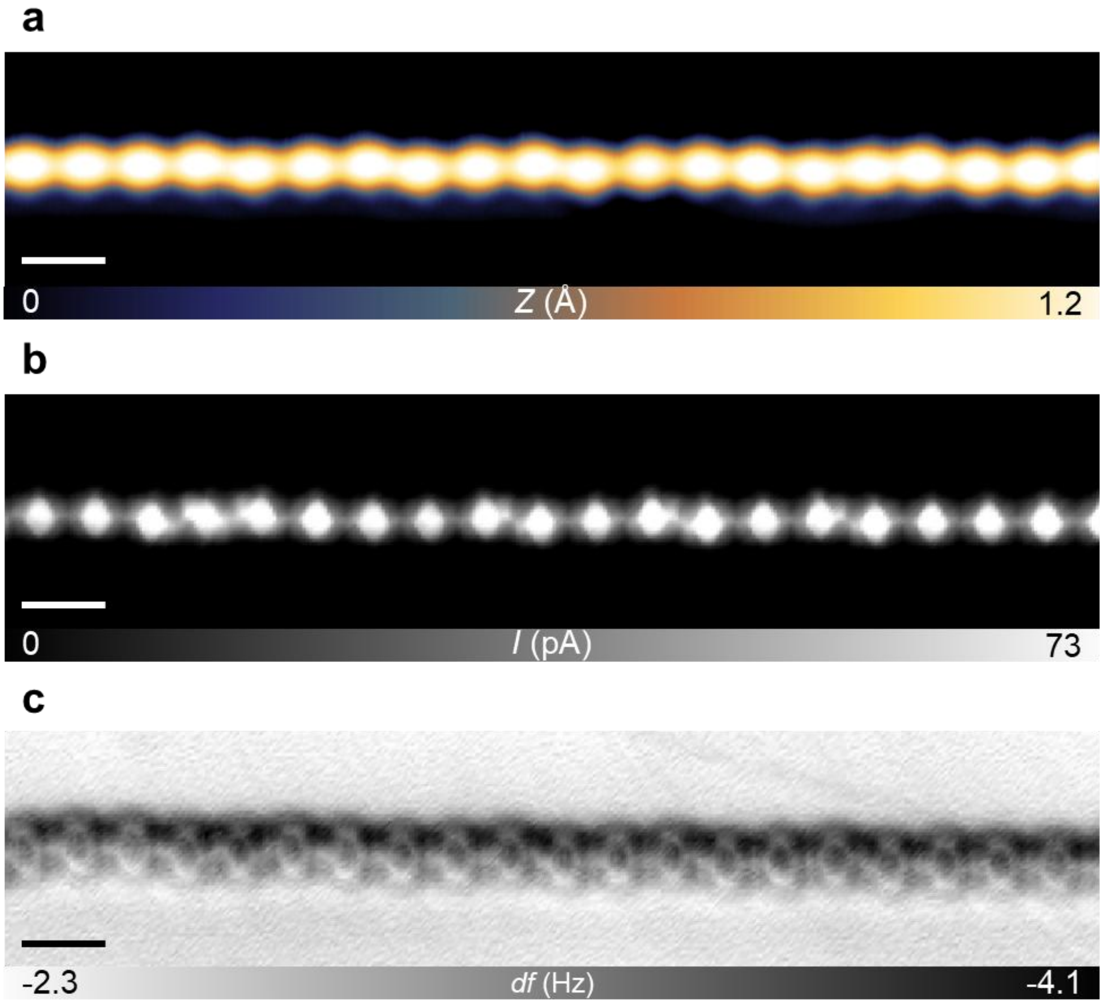

Supplementary Fig. 22 | Long-ordered polymer 2C. **a-c**, STM image (**a**), BR-STM image (**b**), nc-AFM image (**c**) of a polymer **2C** comprising uniform linkages *L*(C≡C). Scanning parameters: 100 mV, 150 pA (**a**) 2 mV, 150 pA, Δz = 140 pm (**b**); 2 mV, Δz = 220 pm (**c**). Scale bars, 1 nm (**a-c**).

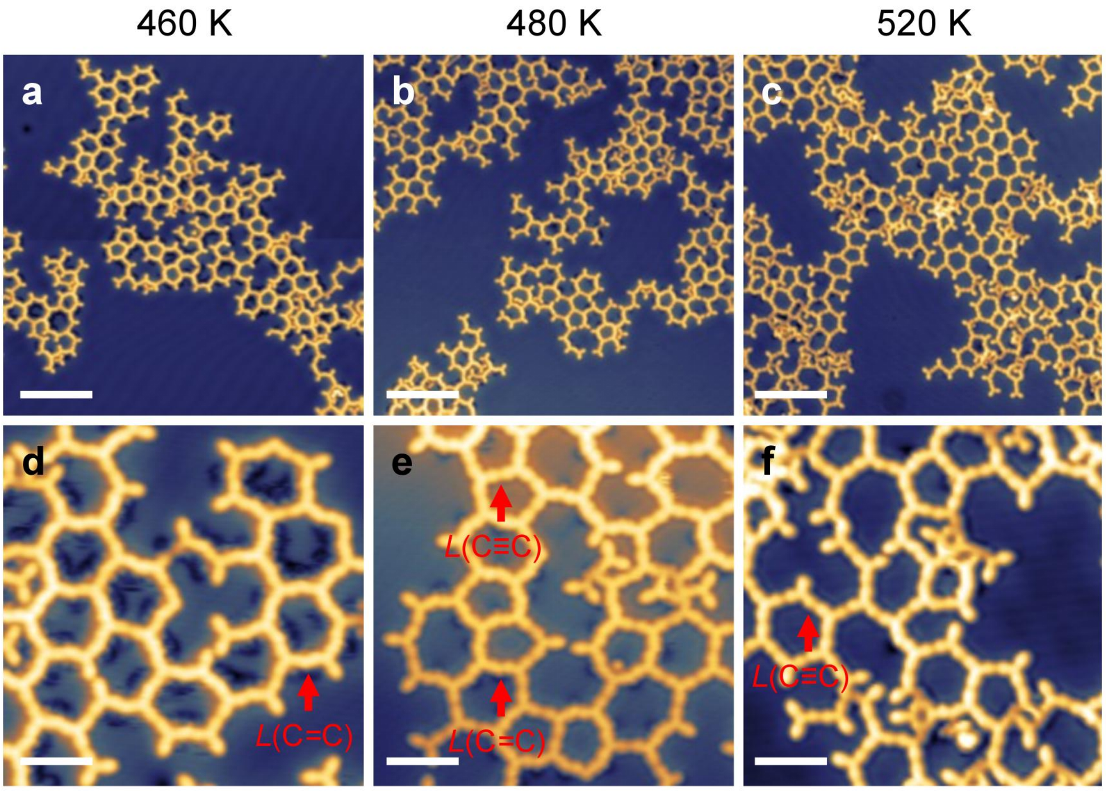

Supplementary Fig. 23 | The transition of carbon hybridization within 2D COFs fabricated *via* annealing M3 to different temperatures on Ag(111). a-c, Large-scale STM images (200 mV, 150 pA) showing the COF structures fabricated by annealing **M3** to 460 K (**a**), 480 K (**b**), and 520 K (**c**) on Ag(111), respectively. **d-f**, High-resolution STM image (400 mV, 150 pA) derived from (**a-c**). Scale bars, 10 nm (**a-c**), 3 nm (**d-f**)

3. NMR spectra and mass spectra of all new compounds

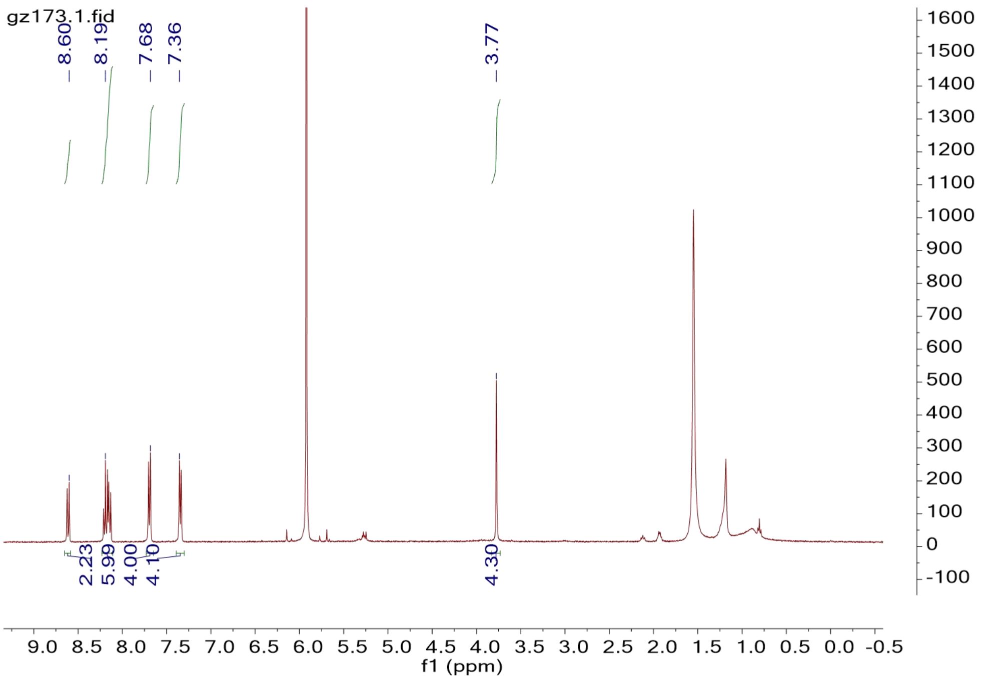

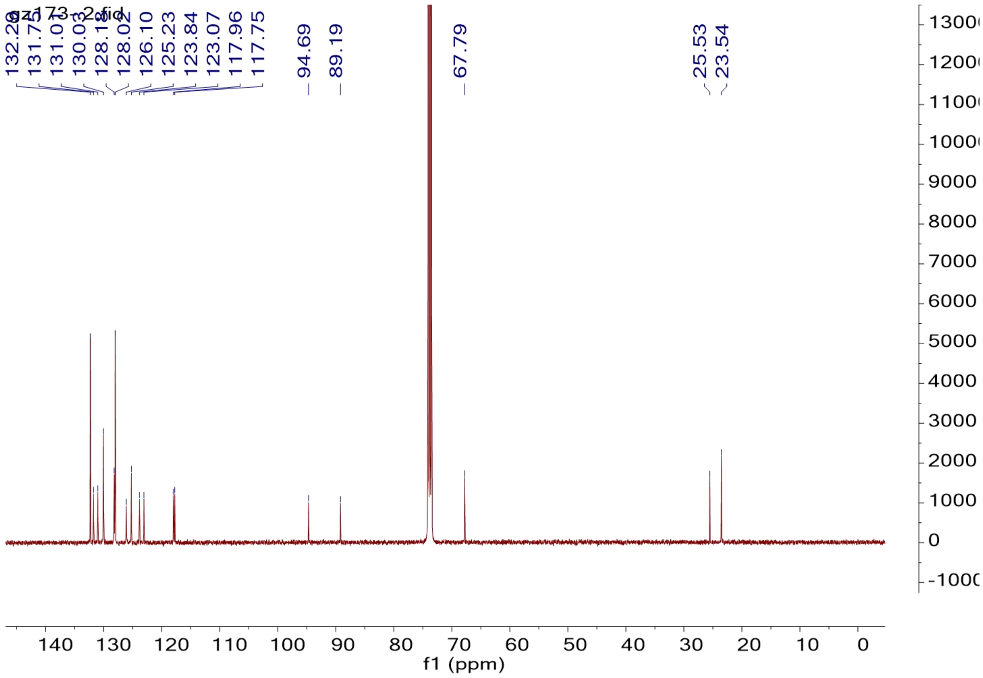

Supplementary Fig. 24 | ^{1}H NMR (top, 400 MHz) and ^{13}C NMR (down, 100 MHz) spectra of compound **M1** (solvent: CD_2Cl_4).

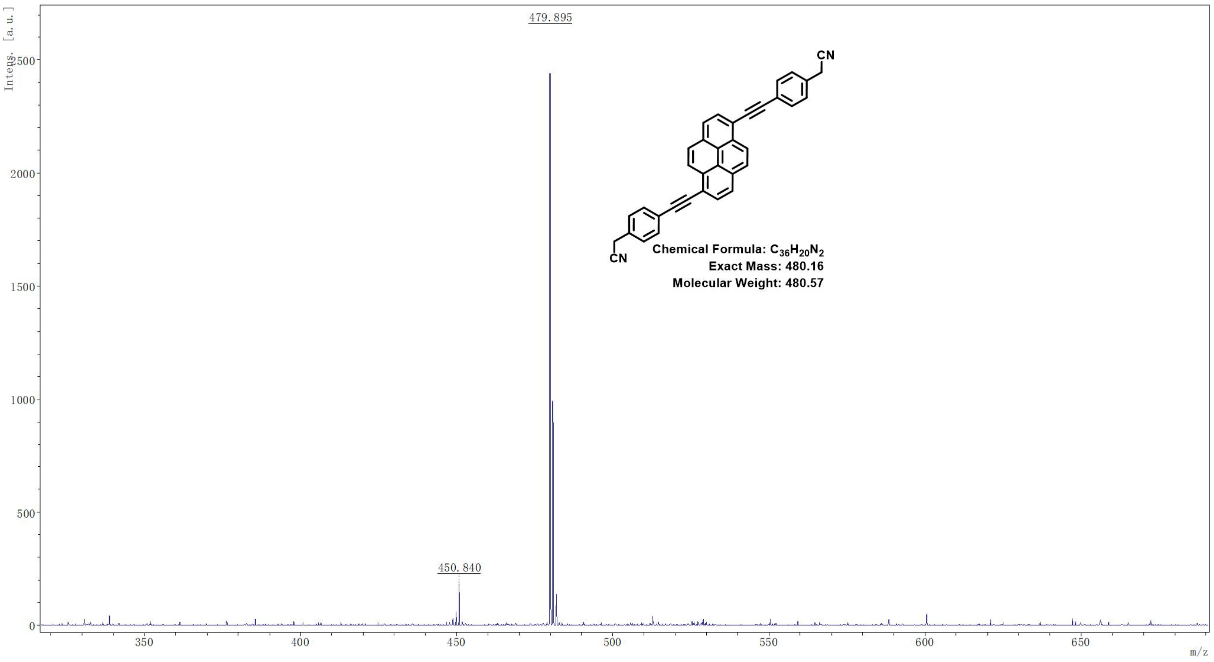

Supplementary Fig. 25 | MALDI-TOF mass spectra of compound **M1**.

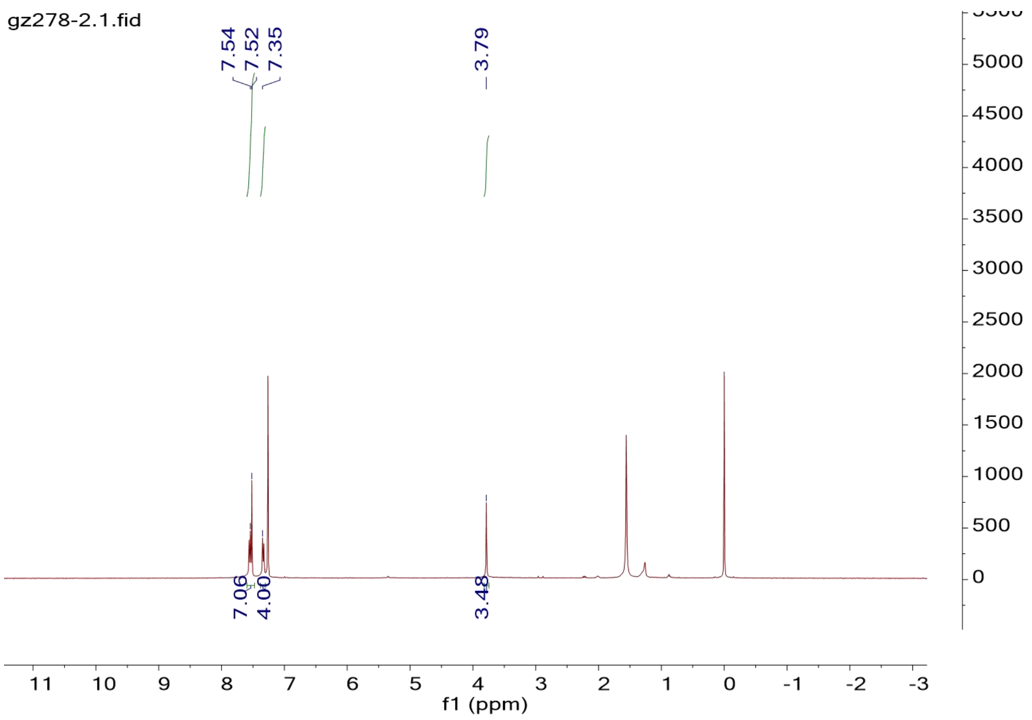

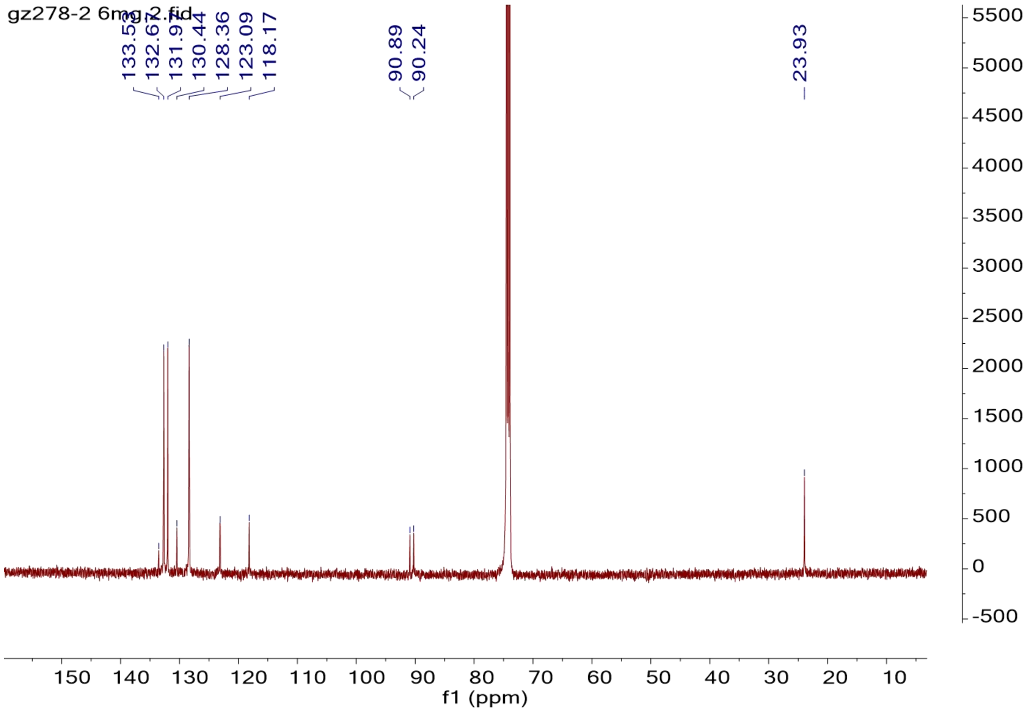

Supplementary Fig. 26 | ^{1}H NMR (top, 400 MHz) and ^{13}C NMR (down, 100 MHz) spectra of compound **M2** (solvent: $CDCl_3$ and CD_2Cl_4, respectively).

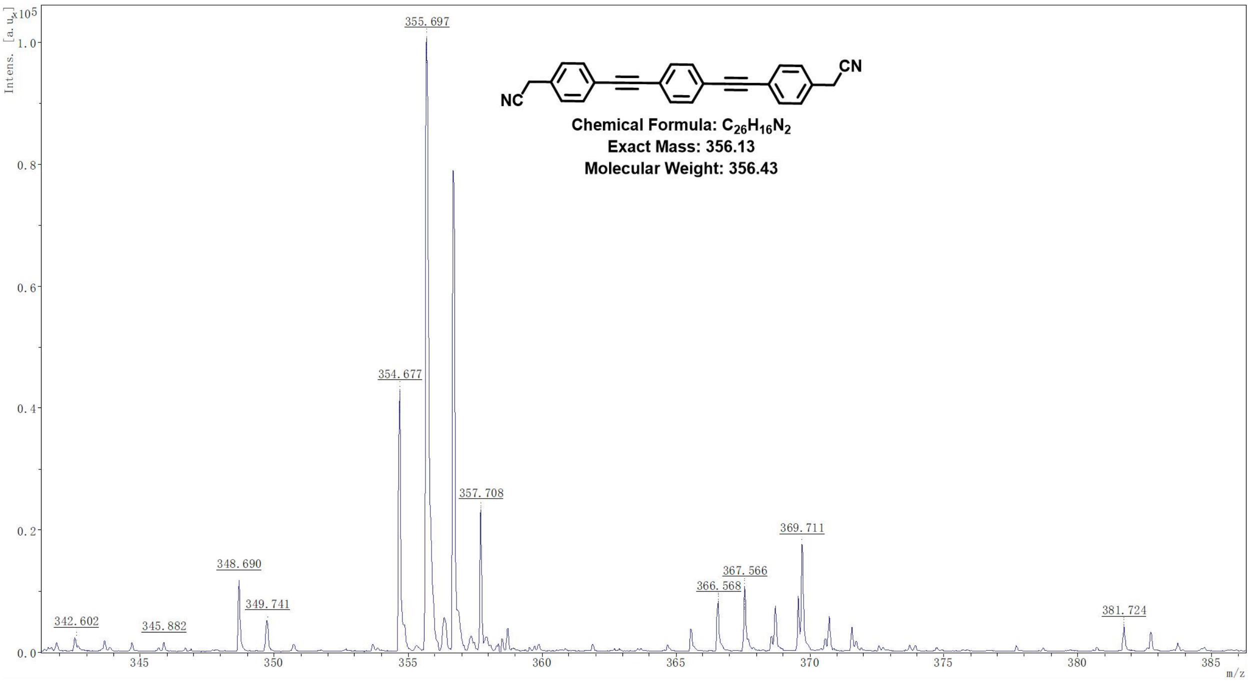

Supplementary Fig. 27 | MALDI-TOF mass spectra of compound **M2**.

References

1. Wang L*, et al.* Synthesis of Hexabenzocoronene-Cored Graphdiyne Nanosheets through Dehydrogenative Coupling on Au(111) Surface. *Angew. Chem. Int. Ed.* **63**, e202411722 (2024).
