# Supplementary material for: Visualizing stepwise evolution of carbon hybridization from sp3 to sp2 and to sp
Source: Nat Commun. 2025 Jan 15;16:690. doi: 10.1038/s41467-024-55719-4 (PMC11735776; doi:10.1038/s41467-024-55719-4)
Supplement: Supplementary file 2 — Description of Additional Supplementary Files [file 41467_2024_55719_MOESM2_ESM.pdf]

Description of Additional Supplementary Files

File name: Supplementary Software 1
Description: Contains coordinates of calculational structures.
