# Supplementary material for: Visualizing stepwise evolution of carbon hybridization from sp3 to sp2 and to sp
Source: Nat Commun. 2025 Jan 15;16:690. doi: 10.1038/s41467-024-55719-4 (PMC11735776; doi:10.1038/s41467-024-55719-4)
Supplement: Supplementary file 3 — Supplementary Software 1 [file 41467_2024_55719_MOESM3_ESM.pdf]

```
************************************************
*                                              *
* Coordinates of structures in format of POSCAR. *
*                                              *
************************************************
```

1. Phenylacetonitrile on Au in Figure 2

```
Au   C    N    H
   1.0000000000000000
  17.3481998443999998   0.0000000000000000   0.0000000000000000
   0.0000000000000000  20.0319004059000001   0.0000000000000000
   0.0000000000000000   0.0000000000000000  25.0000000000000000
Au   C    N    H
144  8  1  7
Selective dynamics
Direct
  0.0000000000000000  0.8333299760000017  0.0955699980000020 F F F
  0.0833299980000035  0.9583299760000016  0.0955699980000020 F F F
  0.0000000000000000  0.5833299760000017  0.0955699980000020 F F F
  0.0833299980000035  0.7083299760000017  0.0955699980000020 F F F
  0.1666699949999995  0.8333299760000017  0.0955699980000020 F F F
  0.2500000000000000  0.9583299760000016  0.0955699980000020 F F F
  0.0000000000000000  0.3333300050000005  0.0955699980000020 F F F
  0.0833299980000035  0.4583300050000005  0.0955699980000020 F F F
  0.1666699949999995  0.5833299760000017  0.0955699980000020 F F F
  0.2500000000000000  0.7083299760000017  0.0955699980000020 F F F
  0.3333300050000005  0.8333299760000017  0.0955699980000020 F F F
  0.4166699949999995  0.9583299760000016  0.0955699980000020 F F F
  0.0000000000000000  0.0833299980000035  0.0955699980000020 F F F
  0.0833299980000035  0.2083300050000005  0.0955699980000020 F F F
  0.1666699949999995  0.3333300050000005  0.0955699980000020 F F F
  0.2500000000000000  0.4583300050000005  0.0955699980000020 F F F
  0.3333300050000005  0.5833299760000017  0.0955699980000020 F F F
  0.4166699949999995  0.7083299760000017  0.0955699980000020 F F F
  0.5000000000000000  0.8333299760000017  0.0955699980000020 F F F
  0.5833299760000017  0.9583299760000016  0.0955699980000020 F F F
  0.1666699949999995  0.0833299980000035  0.0955699980000020 F F F
  0.2500000000000000  0.2083300050000005  0.0955699980000020 F F F
  0.3333300050000005  0.3333300050000005  0.0955699980000020 F F F
  0.4166699949999995  0.4583300050000005  0.0955699980000020 F F F
  0.5000000000000000  0.5833299760000017  0.0955699980000020 F F F
  0.5833299760000017  0.7083299760000017  0.0955699980000020 F F F
  0.6666700239999983  0.8333299760000017  0.0955699980000020 F F F
  0.7500000000000000  0.9583299760000016  0.0955699980000020 F F F
  0.3333300050000005  0.0833299980000035  0.0955699980000020 F F F
  0.4166699949999995  0.2083300050000005  0.0955699980000020 F F F
  0.5000000000000000  0.3333300050000005  0.0955699980000020 F F F
  0.5833299760000017  0.4583300050000005  0.0955699980000020 F F F
  0.6666700239999983  0.5833299760000017  0.0955699980000020 F F F
  0.7500000000000000  0.7083299760000017  0.0955699980000020 F F F
  0.8333299760000017  0.8333299760000017  0.0955699980000020 F F F
  0.9166700239999984  0.9583299760000016  0.0955699980000020 F F F
  0.5000000000000000  0.0833299980000035  0.0955699980000020 F F F
  0.5833299760000017  0.2083300050000005  0.0955699980000020 F F F
  0.6666700239999983  0.3333300050000005  0.0955699980000020 F F F
  0.7500000000000000  0.4583300050000005  0.0955699980000020 F F F
  0.8333299760000017  0.5833299760000017  0.0955699980000020 F F F
  0.9166700239999984  0.7083299760000017  0.0955699980000020 F F F
```

| | | | | | |
|---|---|---|---|---|---|
| 0.6666700239999983 | 0.0833299980000035 | 0.0955699980000020 | F | F | F |
| 0.7500000000000000 | 0.2083300050000005 | 0.0955699980000020 | F | F | F |
| 0.8333299760000017 | 0.3333300050000005 | 0.0955699980000020 | F | F | F |
| 0.9166700239999984 | 0.4583300050000005 | 0.0955699980000020 | F | F | F |
| 0.8333299760000017 | 0.0833299980000035 | 0.0955699980000020 | F | F | F |
| 0.9166700239999984 | 0.2083300050000005 | 0.0955699980000020 | F | F | F |
| 0.0000000000000000 | 0.0000000000000000 | 0.1939200010000022 | F | F | F |
| 0.0000000000000000 | 0.7500000000000000 | 0.1939200010000022 | F | F | F |
| 0.0833299980000035 | 0.8750000000000000 | 0.1939200010000022 | F | F | F |
| 0.1666699949999995 | 0.0000000000000000 | 0.1939200010000022 | F | F | F |
| 0.0000000000000000 | 0.5000000000000000 | 0.1939200010000022 | F | F | F |
| 0.0833299980000035 | 0.6250000000000000 | 0.1939200010000022 | F | F | F |
| 0.1666699949999995 | 0.7500000000000000 | 0.1939200010000022 | F | F | F |
| 0.2500000000000000 | 0.8750000000000000 | 0.1939200010000022 | F | F | F |
| 0.3333300050000005 | 0.0000000000000000 | 0.1939200010000022 | F | F | F |
| 0.0000000000000000 | 0.2500000000000000 | 0.1939200010000022 | F | F | F |
| 0.0833299980000035 | 0.3750000000000000 | 0.1939200010000022 | F | F | F |
| 0.1666699949999995 | 0.5000000000000000 | 0.1939200010000022 | F | F | F |
| 0.2500000000000000 | 0.6250000000000000 | 0.1939200010000022 | F | F | F |
| 0.3333300050000005 | 0.7500000000000000 | 0.1939200010000022 | F | F | F |
| 0.4166699949999995 | 0.8750000000000000 | 0.1939200010000022 | F | F | F |
| 0.5000000000000000 | 0.0000000000000000 | 0.1939200010000022 | F | F | F |
| 0.0833299980000035 | 0.1250000000000000 | 0.1939200010000022 | F | F | F |
| 0.1666699949999995 | 0.2500000000000000 | 0.1939200010000022 | F | F | F |
| 0.2500000000000000 | 0.3750000000000000 | 0.1939200010000022 | F | F | F |
| 0.3333300050000005 | 0.5000000000000000 | 0.1939200010000022 | F | F | F |
| 0.4166699949999995 | 0.6250000000000000 | 0.1939200010000022 | F | F | F |
| 0.5000000000000000 | 0.7500000000000000 | 0.1939200010000022 | F | F | F |
| 0.5833299760000017 | 0.8750000000000000 | 0.1939200010000022 | F | F | F |
| 0.6666700239999983 | 0.0000000000000000 | 0.1939200010000022 | F | F | F |
| 0.2500000000000000 | 0.1250000000000000 | 0.1939200010000022 | F | F | F |
| 0.3333300050000005 | 0.2500000000000000 | 0.1939200010000022 | F | F | F |
| 0.4166699949999995 | 0.3750000000000000 | 0.1939200010000022 | F | F | F |
| 0.5000000000000000 | 0.5000000000000000 | 0.1939200010000022 | F | F | F |
| 0.5833299760000017 | 0.6250000000000000 | 0.1939200010000022 | F | F | F |
| 0.6666700239999983 | 0.7500000000000000 | 0.1939200010000022 | F | F | F |
| 0.7500000000000000 | 0.8750000000000000 | 0.1939200010000022 | F | F | F |
| 0.8333299760000017 | 0.0000000000000000 | 0.1939200010000022 | F | F | F |
| 0.4166699949999995 | 0.1250000000000000 | 0.1939200010000022 | F | F | F |
| 0.5000000000000000 | 0.2500000000000000 | 0.1939200010000022 | F | F | F |
| 0.5833299760000017 | 0.3750000000000000 | 0.1939200010000022 | F | F | F |
| 0.6666700239999983 | 0.5000000000000000 | 0.1939200010000022 | F | F | F |
| 0.7500000000000000 | 0.6250000000000000 | 0.1939200010000022 | F | F | F |
| 0.8333299760000017 | 0.7500000000000000 | 0.1939200010000022 | F | F | F |
| 0.9166700239999984 | 0.8750000000000000 | 0.1939200010000022 | F | F | F |
| 0.5833299760000017 | 0.1250000000000000 | 0.1939200010000022 | F | F | F |
| 0.6666700239999983 | 0.2500000000000000 | 0.1939200010000022 | F | F | F |
| 0.7500000000000000 | 0.3750000000000000 | 0.1939200010000022 | F | F | F |
| 0.8333299760000017 | 0.5000000000000000 | 0.1939200010000022 | F | F | F |
| 0.9166700239999984 | 0.6250000000000000 | 0.1939200010000022 | F | F | F |
| 0.7500000000000000 | 0.1250000000000000 | 0.1939200010000022 | F | F | F |
| 0.8333299760000017 | 0.2500000000000000 | 0.1939200010000022 | F | F | F |
| 0.9166700239999984 | 0.3750000000000000 | 0.1939200010000022 | F | F | F |
| 0.9166700239999984 | 0.1250000000000000 | 0.1939200010000022 | F | F | F |
| 0.9998954098147774 | 0.9136377022584092 | 0.2919787086258063 | T | T | T |
| 0.9996532216644844 | 0.6636408589032007 | 0.2919390490455282 | T | T | T |
| 0.0830687696090134 | 0.7886189617719702 | 0.2922494777680740 | T | T | T |
| 0.1665771655345980 | 0.9137979534139988 | 0.2922223395574844 | T | T | T |
| 0.9999845521540400 | 0.4137458163022907 | 0.2920807574289066 | T | T | T |
| 0.0833069849891618 | 0.5387538564696537 | 0.2918767819273814 | T | T | T |

| | | | | | |
|---|---|---|---|---|---|
| 0.1663730148665741 | 0.6638871338280702 | 0.2917257166427501 | T | T | T |
| 0.2494747596910624 | 0.7890737079856683 | 0.2918331147115915 | T | T | T |
| 0.3330114783432282 | 0.9145161013728104 | 0.2920327904871363 | T | T | T |
| 0.9999252019048440 | 0.1635396475035414 | 0.2920323375997744 | T | T | T |
| 0.0832604183041638 | 0.2886233752454692 | 0.2919929654225407 | T | T | T |
| 0.1666562115556934 | 0.4136134025111955 | 0.2921719026163259 | T | T | T |
| 0.2501383143172408 | 0.5382546901297344 | 0.2912796005725127 | T | T | T |
| 0.3332114882691913 | 0.6641525119253875 | 0.2901483251960627 | T | T | T |
| 0.4164490560417202 | 0.7898743517900880 | 0.2906267070677173 | T | T | T |
| 0.5002362065972531 | 0.9144033431134280 | 0.2919431299114363 | T | T | T |
| 0.0831947353795558 | 0.0387519355160144 | 0.2921489843283348 | T | T | T |
| 0.1665915051242796 | 0.1636949131809436 | 0.2919937014534078 | T | T | T |
| 0.2500617510705830 | 0.2885939043298109 | 0.2920874873048413 | T | T | T |
| 0.3334189469991165 | 0.4134527498470009 | 0.2925127462975560 | T | T | T |
| 0.4164737576322714 | 0.5386485686857598 | 0.2925178755433210 | T | T | T |
| 0.5001294905420158 | 0.6635594134636672 | 0.2912961722633478 | T | T | T |
| 0.5835245488732729 | 0.7888625883573777 | 0.2915580957126184 | T | T | T |
| 0.6666701282820099 | 0.9135706137929001 | 0.2924768933778210 | T | T | T |
| 0.2498397077508753 | 0.0390838576596174 | 0.2922420329267230 | T | T | T |
| 0.3334447067675165 | 0.1637469345352992 | 0.2920586423481930 | T | T | T |
| 0.4168369765818424 | 0.2885791064814920 | 0.2921660109142575 | T | T | T |
| 0.4999486542301000 | 0.4137152550813190 | 0.2921936635716847 | T | T | T |
| 0.5830282922228289 | 0.5386242120053255 | 0.2926008265343332 | T | T | T |
| 0.6663763173912195 | 0.6635616125054260 | 0.2922286211599465 | T | T | T |
| 0.7497528797613751 | 0.7883919496474396 | 0.2923789635630676 | T | T | T |
| 0.8332214511417864 | 0.9134503208835860 | 0.2920520798721921 | T | T | T |
| 0.4167068320570886 | 0.0390360296057291 | 0.2923325219525432 | T | T | T |
| 0.5001959475040417 | 0.1636737827963325 | 0.2920692321072338 | T | T | T |
| 0.5834710850841415 | 0.2885986393173824 | 0.2921621958314923 | T | T | T |
| 0.6666103830803819 | 0.4137514110549390 | 0.2919782909680712 | T | T | T |
| 0.7497299107141546 | 0.5388079495331626 | 0.2922303492319378 | T | T | T |
| 0.8329987628723150 | 0.6635420070111226 | 0.2920717790904910 | T | T | T |
| 0.9164464653215366 | 0.7885003794476909 | 0.2921127336803114 | T | T | T |
| 0.5836057741500937 | 0.0389176257542530 | 0.2923222661550772 | T | T | T |
| 0.6669150043241365 | 0.1635972968261276 | 0.2920009490997093 | T | T | T |
| 0.7500971136072639 | 0.2886321377972317 | 0.2920842380693449 | T | T | T |
| 0.8333045883695708 | 0.4138469951546054 | 0.2919757802404697 | T | T | T |
| 0.9166156035351678 | 0.5388436936789025 | 0.2922868846742346 | T | T | T |
| 0.7501325568343070 | 0.0384879965333633 | 0.2922828099028028 | T | T | T |
| 0.8334106652709596 | 0.1634890881836606 | 0.2920228043999551 | T | T | T |
| 0.9166267391009818 | 0.2886555565884081 | 0.2919983754892960 | T | T | T |
| 0.9166037946949880 | 0.0385677985281086 | 0.2924048224342756 | T | T | T |
| 0.4678124994691966 | 0.7802735086434552 | 0.4180066452138831 | T | T | T |
| 0.4994253701406859 | 0.7160261561845011 | 0.4203977844081910 | T | T | T |
| 0.4512201856889142 | 0.6603746907526252 | 0.4236412655377711 | T | T | T |
| 0.3708032403921185 | 0.6682038288221840 | 0.4248283039024359 | T | T | T |
| 0.3394640815013577 | 0.7324299753405632 | 0.4218914283389659 | T | T | T |
| 0.3878106846623792 | 0.7882551312616117 | 0.4184611763833391 | T | T | T |
| 0.3209944907933442 | 0.6060148851444191 | 0.4294284558463400 | T | T | T |
| 0.2389634197025343 | 0.6179029573223019 | 0.4220931908225302 | T | T | T |
| 0.1735127035027070 | 0.6282331888347763 | 0.4149130687805080 | T | T | T |
| 0.5618187375782607 | 0.7092120925540929 | 0.4192654082322403 | T | T | T |
| 0.4762151070801383 | 0.6103269228595825 | 0.4249521923714152 | T | T | T |
| 0.2769763532541751 | 0.7391152338101094 | 0.4219435186503445 | T | T | T |
| 0.3625813015417843 | 0.8380758446192280 | 0.4160494492890550 | T | T | T |
| 0.3296801316655038 | 0.5819356280576299 | 0.4686759596862093 | T | T | T |
| 0.3389400241024985 | 0.5690748022022580 | 0.3988919199177646 | T | T | T |
| 0.5054164976270549 | 0.8238007731769330 | 0.4152097847438169 | T | T | T |

2. Ethylbenzene on Au in Figure 2.

```
POSCAR\(2)
   1.0000000000000000
    17.3481998443999998    0.0000000000000000    0.0000000000000000
     0.0000000000000000   20.0319004059000001    0.0000000000000000
     0.0000000000000000    0.0000000000000000   25.0000000000000000
   Au   C    H
   144     8    10
Selective dynamics
Direct
  0.0000000000000000  0.8333299760000017  0.0955699980000020   F   F   F
  0.0833299980000035  0.9583299760000017  0.0955699980000020   F   F   F
  0.0000000000000000  0.5833299760000017  0.0955699980000020   F   F   F
  0.0833299980000035  0.7083299760000017  0.0955699980000020   F   F   F
  0.1666699949999995  0.8333299760000017  0.0955699980000020   F   F   F
  0.2500000000000000  0.9583299760000017  0.0955699980000020   F   F   F
  0.0000000000000000  0.3333300050000005  0.0955699980000020   F   F   F
  0.0833299980000035  0.4583300050000005  0.0955699980000020   F   F   F
  0.1666699949999995  0.5833299760000017  0.0955699980000020   F   F   F
  0.2500000000000000  0.7083299760000017  0.0955699980000020   F   F   F
  0.3333300050000005  0.8333299760000017  0.0955699980000020   F   F   F
  0.4166699949999995  0.9583299760000017  0.0955699980000020   F   F   F
  0.0000000000000000  0.0833299980000035  0.0955699980000020   F   F   F
  0.0833299980000035  0.2083300050000005  0.0955699980000020   F   F   F
  0.1666699949999995  0.3333300050000005  0.0955699980000020   F   F   F
  0.2500000000000000  0.4583300050000005  0.0955699980000020   F   F   F
  0.3333300050000005  0.5833299760000017  0.0955699980000020   F   F   F
  0.4166699949999995  0.7083299760000017  0.0955699980000020   F   F   F
  0.5000000000000000  0.8333299760000017  0.0955699980000020   F   F   F
  0.5833299760000017  0.9583299760000017  0.0955699980000020   F   F   F
  0.1666699949999995  0.0833299980000035  0.0955699980000020   F   F   F
  0.2500000000000000  0.2083300050000005  0.0955699980000020   F   F   F
  0.3333300050000005  0.3333300050000005  0.0955699980000020   F   F   F
  0.4166699949999995  0.4583300050000005  0.0955699980000020   F   F   F
  0.5000000000000000  0.5833299760000017  0.0955699980000020   F   F   F
  0.5833299760000017  0.7083299760000017  0.0955699980000020   F   F   F
  0.6666700239999983  0.8333299760000017  0.0955699980000020   F   F   F
  0.7500000000000000  0.9583299760000017  0.0955699980000020   F   F   F
  0.3333300050000005  0.0833299980000035  0.0955699980000020   F   F   F
  0.4166699949999995  0.2083300050000005  0.0955699980000020   F   F   F
  0.5000000000000000  0.3333300050000005  0.0955699980000020   F   F   F
  0.5833299760000017  0.4583300050000005  0.0955699980000020   F   F   F
  0.6666700239999983  0.5833299760000017  0.0955699980000020   F   F   F
  0.7500000000000000  0.7083299760000017  0.0955699980000020   F   F   F
  0.8333299760000017  0.8333299760000017  0.0955699980000020   F   F   F
  0.9166700239999983  0.9583299760000017  0.0955699980000020   F   F   F
  0.5000000000000000  0.0833299980000035  0.0955699980000020   F   F   F
  0.5833299760000017  0.2083300050000005  0.0955699980000020   F   F   F
  0.6666700239999983  0.3333300050000005  0.0955699980000020   F   F   F
  0.7500000000000000  0.4583300050000005  0.0955699980000020   F   F   F
  0.8333299760000017  0.5833299760000017  0.0955699980000020   F   F   F
  0.9166700239999983  0.7083299760000017  0.0955699980000020   F   F   F
  0.6666700239999983  0.0833299980000035  0.0955699980000020   F   F   F
  0.7500000000000000  0.2083300050000005  0.0955699980000020   F   F   F
  0.8333299760000017  0.3333300050000005  0.0955699980000020   F   F   F
  0.9166700239999983  0.4583300050000005  0.0955699980000020   F   F   F
  0.8333299760000017  0.0833299980000035  0.0955699980000020   F   F   F
  0.9166700239999983  0.2083300050000005  0.0955699980000020   F   F   F
  0.0000000000000000  0.0000000000000000  0.1939200010000022   F   F   F
```

| | | | | | |
|---|---|---|---|---|---|
| 0.0000000000000000 | 0.7500000000000000 | 0.1939200010000022 | F | F | F |
| 0.0833299980000035 | 0.8750000000000000 | 0.1939200010000022 | F | F | F |
| 0.1666699949999995 | 0.0000000000000000 | 0.1939200010000022 | F | F | F |
| 0.0000000000000000 | 0.5000000000000000 | 0.1939200010000022 | F | F | F |
| 0.0833299980000035 | 0.6250000000000000 | 0.1939200010000022 | F | F | F |
| 0.1666699949999995 | 0.7500000000000000 | 0.1939200010000022 | F | F | F |
| 0.2500000000000000 | 0.8750000000000000 | 0.1939200010000022 | F | F | F |
| 0.3333300050000005 | 0.0000000000000000 | 0.1939200010000022 | F | F | F |
| 0.0000000000000000 | 0.2500000000000000 | 0.1939200010000022 | F | F | F |
| 0.0833299980000035 | 0.3750000000000000 | 0.1939200010000022 | F | F | F |
| 0.1666699949999995 | 0.5000000000000000 | 0.1939200010000022 | F | F | F |
| 0.2500000000000000 | 0.6250000000000000 | 0.1939200010000022 | F | F | F |
| 0.3333300050000005 | 0.7500000000000000 | 0.1939200010000022 | F | F | F |
| 0.4166699949999995 | 0.8750000000000000 | 0.1939200010000022 | F | F | F |
| 0.5000000000000000 | 0.0000000000000000 | 0.1939200010000022 | F | F | F |
| 0.0833299980000035 | 0.1250000000000000 | 0.1939200010000022 | F | F | F |
| 0.1666699949999995 | 0.2500000000000000 | 0.1939200010000022 | F | F | F |
| 0.2500000000000000 | 0.3750000000000000 | 0.1939200010000022 | F | F | F |
| 0.3333300050000005 | 0.5000000000000000 | 0.1939200010000022 | F | F | F |
| 0.4166699949999995 | 0.6250000000000000 | 0.1939200010000022 | F | F | F |
| 0.5000000000000000 | 0.7500000000000000 | 0.1939200010000022 | F | F | F |
| 0.5833299760000017 | 0.8750000000000000 | 0.1939200010000022 | F | F | F |
| 0.6666700239999983 | 0.0000000000000000 | 0.1939200010000022 | F | F | F |
| 0.2500000000000000 | 0.1250000000000000 | 0.1939200010000022 | F | F | F |
| 0.3333300050000005 | 0.2500000000000000 | 0.1939200010000022 | F | F | F |
| 0.4166699949999995 | 0.3750000000000000 | 0.1939200010000022 | F | F | F |
| 0.5000000000000000 | 0.5000000000000000 | 0.1939200010000022 | F | F | F |
| 0.5833299760000017 | 0.6250000000000000 | 0.1939200010000022 | F | F | F |
| 0.6666700239999983 | 0.7500000000000000 | 0.1939200010000022 | F | F | F |
| 0.7500000000000000 | 0.8750000000000000 | 0.1939200010000022 | F | F | F |
| 0.8333299760000017 | 0.0000000000000000 | 0.1939200010000022 | F | F | F |
| 0.4166699949999995 | 0.1250000000000000 | 0.1939200010000022 | F | F | F |
| 0.5000000000000000 | 0.2500000000000000 | 0.1939200010000022 | F | F | F |
| 0.5833299760000017 | 0.3750000000000000 | 0.1939200010000022 | F | F | F |
| 0.6666700239999983 | 0.5000000000000000 | 0.1939200010000022 | F | F | F |
| 0.7500000000000000 | 0.6250000000000000 | 0.1939200010000022 | F | F | F |
| 0.8333299760000017 | 0.7500000000000000 | 0.1939200010000022 | F | F | F |
| 0.9166700239999983 | 0.8750000000000000 | 0.1939200010000022 | F | F | F |
| 0.5833299760000017 | 0.1250000000000000 | 0.1939200010000022 | F | F | F |
| 0.6666700239999983 | 0.2500000000000000 | 0.1939200010000022 | F | F | F |
| 0.7500000000000000 | 0.3750000000000000 | 0.1939200010000022 | F | F | F |
| 0.8333299760000017 | 0.5000000000000000 | 0.1939200010000022 | F | F | F |
| 0.9166700239999983 | 0.6250000000000000 | 0.1939200010000022 | F | F | F |
| 0.7500000000000000 | 0.1250000000000000 | 0.1939200010000022 | F | F | F |
| 0.8333299760000017 | 0.2500000000000000 | 0.1939200010000022 | F | F | F |
| 0.9166700239999983 | 0.3750000000000000 | 0.1939200010000022 | F | F | F |
| 0.9166700239999983 | 0.1250000000000000 | 0.1939200010000022 | F | F | F |
| 0.9999750204739399 | 0.9135506878572954 | 0.2921492490026267 | T | T | T |
| 0.0000143715291188 | 0.6635182536717679 | 0.2921074594630042 | T | T | T |
| 0.0831601674822764 | 0.7886239521587086 | 0.2924751653564144 | T | T | T |
| 0.1665585813592116 | 0.9138625447465145 | 0.2922680815143366 | T | T | T |
| 0.0000222474995866 | 0.4137709229908708 | 0.2921310744840611 | T | T | T |
| 0.0834335725168387 | 0.5387917972283560 | 0.2924312314819915 | T | T | T |
| 0.1664185849210753 | 0.6639368301428163 | 0.2916515056397486 | T | T | T |
| 0.2493963149657787 | 0.7892058402599247 | 0.2917630041785344 | T | T | T |
| 0.3329492036467769 | 0.9147424618496416 | 0.2918356045173809 | T | T | T |
| 0.9999514577483808 | 0.1635245440976476 | 0.2920415106487368 | T | T | T |
| 0.0832516575616040 | 0.2886519091778368 | 0.2920153620922363 | T | T | T |
| 0.1666338763964674 | 0.4136849266224232 | 0.2921880000482489 | T | T | T |
| 0.2499568593636496 | 0.5385173449685539 | 0.2915028755313145 | T | T | T |

| | | | | | |
|---|---|---|---|---|---|
| 0.3330930108621786 | 0.6643094447257454 | 0.2900762012994633 | T | T | T |
| 0.4166509653607591 | 0.7902612261478055 | 0.2906331068344414 | T | T | T |
| 0.5004233291206930 | 0.9147728116115843 | 0.2917495198822806 | T | T | T |
| 0.0831836089467615 | 0.0386801110495725 | 0.2923164145933512 | T | T | T |
| 0.1665137499383249 | 0.1637150857960705 | 0.2920036440088296 | T | T | T |
| 0.2499759945260678 | 0.2886573200300333 | 0.2920717809269746 | T | T | T |
| 0.3333482259291429 | 0.4136420142157249 | 0.2922194347154701 | T | T | T |
| 0.4166039261291914 | 0.5387233928446378 | 0.2920323707608432 | T | T | T |
| 0.5003653919025908 | 0.6635510112979759 | 0.2911709469859503 | T | T | T |
| 0.5839987566321057 | 0.7890768363190972 | 0.2910960197237245 | T | T | T |
| 0.6668528538987175 | 0.9137766468312094 | 0.2923937724097740 | T | T | T |
| 0.2497936956596050 | 0.0391766325168277 | 0.2922335079376950 | T | T | T |
| 0.3333580017508098 | 0.1638406310837155 | 0.2920185839034133 | T | T | T |
| 0.4167665794996580 | 0.2886700717128150 | 0.2920835751824313 | T | T | T |
| 0.4999762906177523 | 0.4137746774501707 | 0.2921356107140340 | T | T | T |
| 0.5832580191225176 | 0.5387022487776534 | 0.2925466470952998 | T | T | T |
| 0.6668009645492674 | 0.6636369350004188 | 0.2921643116504242 | T | T | T |
| 0.7501693019219999 | 0.7884343386009078 | 0.2924055393014910 | T | T | T |
| 0.8333497747369520 | 0.9135103609098607 | 0.2921450267571544 | T | T | T |
| 0.4167017626808232 | 0.0391728486053253 | 0.2922853505810821 | T | T | T |
| 0.5001479632531556 | 0.1637975559582756 | 0.2920303297177256 | T | T | T |
| 0.5834757387606686 | 0.2886756164136699 | 0.2920653792355310 | T | T | T |
| 0.6666998199658636 | 0.4138401626736510 | 0.2919052841033363 | T | T | T |
| 0.7499526068899627 | 0.5388355177803703 | 0.2923399802656519 | T | T | T |
| 0.8334032782589631 | 0.6634759328939666 | 0.2920751509000254 | T | T | T |
| 0.9166626098133790 | 0.7884613569527659 | 0.2920933070246932 | T | T | T |
| 0.5836827139889849 | 0.0391147993291057 | 0.2922160621617707 | T | T | T |
| 0.6669596100910127 | 0.1636876050331537 | 0.2919732188480424 | T | T | T |
| 0.7501443626799258 | 0.2886512479436618 | 0.2920699699236664 | T | T | T |
| 0.8333636534908209 | 0.4137925853834190 | 0.2920358605359894 | T | T | T |
| 0.9167579667017947 | 0.5387382406912025 | 0.2923852576687906 | T | T | T |
| 0.7502624510736879 | 0.0386060462455333 | 0.2922757953118946 | T | T | T |
| 0.8334647985081105 | 0.1635047959067890 | 0.2920391297185034 | T | T | T |
| 0.9166786890708920 | 0.2886422635967989 | 0.2920525806598278 | T | T | T |
| 0.9167137689134890 | 0.0385239685975165 | 0.2924327854238697 | T | T | T |
| 0.4681752507310932 | 0.7799389794791836 | 0.4133828095762624 | T | T | T |
| 0.4997820277332821 | 0.7156285044727682 | 0.4164792922922587 | T | T | T |
| 0.4515209771213051 | 0.6602070997309184 | 0.4210511439025666 | T | T | T |
| 0.3707212019257824 | 0.6671847305756984 | 0.4230209398941156 | T | T | T |
| 0.3399585414397848 | 0.7317183759432950 | 0.4191813322089430 | T | T | T |
| 0.3881330372935636 | 0.7876438668227266 | 0.4143916791960720 | T | T | T |
| 0.3212917430528819 | 0.6055128814802231 | 0.4292888634037695 | T | T | T |
| 0.2345474169931592 | 0.6165718867192922 | 0.4326375662808246 | T | T | T |
| 0.5622069873698479 | 0.7088883534862731 | 0.4153509932084845 | T | T | T |
| 0.4767002306362329 | 0.6102637802541153 | 0.4234567876854314 | T | T | T |
| 0.2776509285204298 | 0.7390453559924026 | 0.4200437480947201 | T | T | T |
| 0.3627837470621316 | 0.8374372395121057 | 0.4118902372484925 | T | T | T |
| 0.3412766373659935 | 0.5781825921471935 | 0.4650319246649630 | T | T | T |
| 0.3340438024266419 | 0.5717555165772623 | 0.3953085506514853 | T | T | T |
| 0.5056613672359020 | 0.8235154578820172 | 0.4101123534698701 | T | T | T |
| 0.2041764133845945 | 0.5686005793182431 | 0.4358801247677135 | T | T | T |
| 0.2123482581331260 | 0.6415951133740236 | 0.3964462871886951 | T | T | T |
| 0.2184839250539667 | 0.6473024883781022 | 0.4673761975111717 | T | T | T |

3. MM in Figure 3.

CONTCAR
1.0
17.3481998444 0.0000000000 0.0000000000

```
     0.0000000000        20.0319004059         0.0000000000
     0.0000000000         0.0000000000        25.0000000000
   Au    C    N    H
  144   16    2   14
Direct
    0.000000000         0.833329976         0.095569998
    0.083329998         0.958329976         0.095569998
    0.000000000         0.583329976         0.095569998
    0.083329998         0.708329976         0.095569998
    0.166669995         0.833329976         0.095569998
    0.250000000         0.958329976         0.095569998
    0.000000000         0.333330005         0.095569998
    0.083329998         0.458330005         0.095569998
    0.166669995         0.583329976         0.095569998
    0.250000000         0.708329976         0.095569998
    0.333330005         0.833329976         0.095569998
    0.416669995         0.958329976         0.095569998
    0.000000000         0.083329998         0.095569998
    0.083329998         0.208330005         0.095569998
    0.166669995         0.333330005         0.095569998
    0.250000000         0.458330005         0.095569998
    0.333330005         0.583329976         0.095569998
    0.416669995         0.708329976         0.095569998
    0.500000000         0.833329976         0.095569998
    0.583329976         0.958329976         0.095569998
    0.166669995         0.083329998         0.095569998
    0.250000000         0.208330005         0.095569998
    0.333330005         0.333330005         0.095569998
    0.416669995         0.458330005         0.095569998
    0.500000000         0.583329976         0.095569998
    0.583329976         0.708329976         0.095569998
    0.666670024         0.833329976         0.095569998
    0.750000000         0.958329976         0.095569998
    0.333330005         0.083329998         0.095569998
    0.416669995         0.208330005         0.095569998
    0.500000000         0.333330005         0.095569998
    0.583329976         0.458330005         0.095569998
    0.666670024         0.583329976         0.095569998
    0.750000000         0.708329976         0.095569998
    0.833329976         0.833329976         0.095569998
    0.916670024         0.958329976         0.095569998
    0.500000000         0.083329998         0.095569998
    0.583329976         0.208330005         0.095569998
    0.666670024         0.333330005         0.095569998
    0.750000000         0.458330005         0.095569998
    0.833329976         0.583329976         0.095569998
    0.916670024         0.708329976         0.095569998
    0.666670024         0.083329998         0.095569998
    0.750000000         0.208330005         0.095569998
    0.833329976         0.333330005         0.095569998
    0.916670024         0.458330005         0.095569998
    0.833329976         0.083329998         0.095569998
    0.916670024         0.208330005         0.095569998
    0.000000000         0.000000000         0.193918005
    0.000000000         0.750000000         0.193918005
    0.083329998         0.875000000         0.193918005
    0.166669995         0.000000000         0.193918005
    0.000000000         0.500000000         0.193918005
    0.083329998         0.625000000         0.193918005
    0.166669995         0.750000000         0.193918005
```

| | | |
|---|---|---|
| 0.250000000 | 0.875000000 | 0.193918005 |
| 0.333330005 | 0.000000000 | 0.193918005 |
| 0.000000000 | 0.250000000 | 0.193918005 |
| 0.083329998 | 0.375000000 | 0.193918005 |
| 0.166669995 | 0.500000000 | 0.193918005 |
| 0.250000000 | 0.625000000 | 0.193918005 |
| 0.333330005 | 0.750000000 | 0.193918005 |
| 0.416669995 | 0.875000000 | 0.193918005 |
| 0.500000000 | 0.000000000 | 0.193918005 |
| 0.083329998 | 0.125000000 | 0.193918005 |
| 0.166669995 | 0.250000000 | 0.193918005 |
| 0.250000000 | 0.375000000 | 0.193918005 |
| 0.333330005 | 0.500000000 | 0.193918005 |
| 0.416669995 | 0.625000000 | 0.193918005 |
| 0.500000000 | 0.750000000 | 0.193918005 |
| 0.583329976 | 0.875000000 | 0.193918005 |
| 0.666670024 | 0.000000000 | 0.193918005 |
| 0.250000000 | 0.125000000 | 0.193918005 |
| 0.333330005 | 0.250000000 | 0.193918005 |
| 0.416669995 | 0.375000000 | 0.193918005 |
| 0.500000000 | 0.500000000 | 0.193918005 |
| 0.583329976 | 0.625000000 | 0.193918005 |
| 0.666670024 | 0.750000000 | 0.193918005 |
| 0.750000000 | 0.875000000 | 0.193918005 |
| 0.833329976 | 0.000000000 | 0.193918005 |
| 0.416669995 | 0.125000000 | 0.193918005 |
| 0.500000000 | 0.250000000 | 0.193918005 |
| 0.583329976 | 0.375000000 | 0.193918005 |
| 0.666670024 | 0.500000000 | 0.193918005 |
| 0.750000000 | 0.625000000 | 0.193918005 |
| 0.833329976 | 0.750000000 | 0.193918005 |
| 0.916670024 | 0.875000000 | 0.193918005 |
| 0.583329976 | 0.125000000 | 0.193918005 |
| 0.666670024 | 0.250000000 | 0.193918005 |
| 0.750000000 | 0.375000000 | 0.193918005 |
| 0.833329976 | 0.500000000 | 0.193918005 |
| 0.916670024 | 0.625000000 | 0.193918005 |
| 0.750000000 | 0.125000000 | 0.193918005 |
| 0.833329976 | 0.250000000 | 0.193918005 |
| 0.916670024 | 0.375000000 | 0.193918005 |
| 0.916670024 | 0.125000000 | 0.193918005 |
| 0.999964893 | 0.913846493 | 0.291899443 |
| 0.999815464 | 0.663598180 | 0.292041242 |
| 0.083053462 | 0.788667738 | 0.292279840 |
| 0.166586936 | 0.913857102 | 0.292228490 |
| 0.000022443 | 0.413816899 | 0.292218804 |
| 0.083386511 | 0.538920581 | 0.292308986 |
| 0.166442156 | 0.664003789 | 0.291902572 |
| 0.249506608 | 0.789092183 | 0.291935503 |
| 0.333005875 | 0.914467990 | 0.292123139 |
| 0.000213178 | 0.163707584 | 0.292074174 |
| 0.083397366 | 0.288845360 | 0.292436361 |
| 0.166381821 | 0.414079905 | 0.292199045 |
| 0.250087440 | 0.538758516 | 0.291667998 |
| 0.333209693 | 0.664216280 | 0.290398985 |
| 0.416460782 | 0.789834738 | 0.290686488 |
| 0.500169337 | 0.914393842 | 0.292074084 |
| 0.083331332 | 0.038894251 | 0.292347670 |
| 0.166772053 | 0.163776502 | 0.292214245 |
| 0.249757439 | 0.288820386 | 0.290928155 |

| | | |
|---|---|---|
| 0.333203435 | 0.414376587 | 0.291604936 |
| 0.416549534 | 0.538920522 | 0.293160290 |
| 0.500064611 | 0.663637102 | 0.291516364 |
| 0.583482444 | 0.788872242 | 0.291581213 |
| 0.666652739 | 0.913683355 | 0.292343765 |
| 0.249883130 | 0.039073966 | 0.292411774 |
| 0.333490610 | 0.163594171 | 0.291721374 |
| 0.417692959 | 0.288648039 | 0.290986717 |
| 0.500039637 | 0.413884670 | 0.289820820 |
| 0.583079278 | 0.538859010 | 0.292528182 |
| 0.666425526 | 0.663557708 | 0.292301685 |
| 0.749783456 | 0.788468122 | 0.292295903 |
| 0.833242476 | 0.913684845 | 0.292008340 |
| 0.416685671 | 0.038965095 | 0.292538166 |
| 0.500147223 | 0.163382649 | 0.292344898 |
| 0.583839059 | 0.288304150 | 0.292178243 |
| 0.666820467 | 0.413772762 | 0.292221993 |
| 0.749777913 | 0.538790703 | 0.292361796 |
| 0.833110690 | 0.663518131 | 0.292093933 |
| 0.916495144 | 0.788595796 | 0.291966319 |
| 0.583581507 | 0.038897678 | 0.292382479 |
| 0.666869879 | 0.163589358 | 0.292138845 |
| 0.750139952 | 0.288667172 | 0.292028040 |
| 0.833353937 | 0.413907886 | 0.291924566 |
| 0.916658640 | 0.538823187 | 0.292333543 |
| 0.750104427 | 0.038694486 | 0.292392880 |
| 0.833491385 | 0.163594410 | 0.292020231 |
| 0.916766226 | 0.288703591 | 0.291938543 |
| 0.916691601 | 0.038768288 | 0.292400599 |
| 0.467272937 | 0.780078828 | 0.419187605 |
| 0.499042690 | 0.715913355 | 0.421622574 |
| 0.450930804 | 0.660220444 | 0.424841970 |
| 0.370510548 | 0.667913139 | 0.425870836 |
| 0.339018285 | 0.732059896 | 0.422994971 |
| 0.387274921 | 0.787939191 | 0.419675380 |
| 0.320883989 | 0.605564475 | 0.429664314 |
| 0.238452062 | 0.617115438 | 0.424325079 |
| 0.294626802 | 0.271687806 | 0.419496506 |
| 0.268084317 | 0.337575883 | 0.421300650 |
| 0.320556223 | 0.390363365 | 0.424218386 |
| 0.400079668 | 0.377939373 | 0.425676823 |
| 0.426425040 | 0.311956674 | 0.423091471 |
| 0.373854488 | 0.259106964 | 0.420150757 |
| 0.454498440 | 0.437175155 | 0.429720074 |
| 0.535856903 | 0.421204001 | 0.423588037 |
| 0.172508091 | 0.626579285 | 0.418803513 |
| 0.601005733 | 0.408588350 | 0.417590499 |
| 0.561445534 | 0.709183455 | 0.420404762 |
| 0.476217747 | 0.610262394 | 0.426023126 |
| 0.276522428 | 0.738622010 | 0.422940224 |
| 0.361937344 | 0.837711394 | 0.417124867 |
| 0.331359833 | 0.579687417 | 0.467971951 |
| 0.337618679 | 0.570446134 | 0.397400975 |
| 0.504783392 | 0.823650837 | 0.416229367 |
| 0.206381083 | 0.348040700 | 0.419843882 |
| 0.299194872 | 0.441674262 | 0.425197482 |
| 0.488250911 | 0.301673621 | 0.423264652 |
| 0.395189345 | 0.207924709 | 0.417861581 |
| 0.446659952 | 0.462787420 | 0.468448848 |
| 0.440003335 | 0.473892182 | 0.398079574 |

0.253772974 0.230395362 0.416947126

4. MM. in Figure 3.

```
CONTCAR
1.0
      17.3481998444         0.0000000000         0.0000000000
       0.0000000000        20.0319004059         0.0000000000
       0.0000000000         0.0000000000        25.0000000000
   Au    C    N    H
  144   16    2   14
Direct
     0.000000000         0.833329976         0.095569998
     0.083329998         0.958329976         0.095569998
     0.000000000         0.583329976         0.095569998
     0.083329998         0.708329976         0.095569998
     0.166669995         0.833329976         0.095569998
     0.250000000         0.958329976         0.095569998
     0.000000000         0.333330005         0.095569998
     0.083329998         0.458330005         0.095569998
     0.166669995         0.583329976         0.095569998
     0.250000000         0.708329976         0.095569998
     0.333330005         0.833329976         0.095569998
     0.416669995         0.958329976         0.095569998
     0.000000000         0.083329998         0.095569998
     0.083329998         0.208330005         0.095569998
     0.166669995         0.333330005         0.095569998
     0.250000000         0.458330005         0.095569998
     0.333330005         0.583329976         0.095569998
     0.416669995         0.708329976         0.095569998
     0.500000000         0.833329976         0.095569998
     0.583329976         0.958329976         0.095569998
     0.166669995         0.083329998         0.095569998
     0.250000000         0.208330005         0.095569998
     0.333330005         0.333330005         0.095569998
     0.416669995         0.458330005         0.095569998
     0.500000000         0.583329976         0.095569998
     0.583329976         0.708329976         0.095569998
     0.666670024         0.833329976         0.095569998
     0.750000000         0.958329976         0.095569998
     0.333330005         0.083329998         0.095569998
     0.416669995         0.208330005         0.095569998
     0.500000000         0.333330005         0.095569998
     0.583329976         0.458330005         0.095569998
     0.666670024         0.583329976         0.095569998
     0.750000000         0.708329976         0.095569998
     0.833329976         0.833329976         0.095569998
     0.916670024         0.958329976         0.095569998
     0.500000000         0.083329998         0.095569998
     0.583329976         0.208330005         0.095569998
     0.666670024         0.333330005         0.095569998
     0.750000000         0.458330005         0.095569998
     0.833329976         0.583329976         0.095569998
     0.916670024         0.708329976         0.095569998
     0.666670024         0.083329998         0.095569998
     0.750000000         0.208330005         0.095569998
     0.833329976         0.333330005         0.095569998
     0.916670024         0.458330005         0.095569998
     0.833329976         0.083329998         0.095569998
```

| | | |
|---|---|---|
| 0.916670024 | 0.208330005 | 0.095569998 |
| 0.000000000 | 0.000000000 | 0.193918005 |
| 0.000000000 | 0.750000000 | 0.193918005 |
| 0.083329998 | 0.875000000 | 0.193918005 |
| 0.166669995 | 0.000000000 | 0.193918005 |
| 0.000000000 | 0.500000000 | 0.193918005 |
| 0.083329998 | 0.625000000 | 0.193918005 |
| 0.166669995 | 0.750000000 | 0.193918005 |
| 0.250000000 | 0.875000000 | 0.193918005 |
| 0.333330005 | 0.000000000 | 0.193918005 |
| 0.000000000 | 0.250000000 | 0.193918005 |
| 0.083329998 | 0.375000000 | 0.193918005 |
| 0.166669995 | 0.500000000 | 0.193918005 |
| 0.250000000 | 0.625000000 | 0.193918005 |
| 0.333330005 | 0.750000000 | 0.193918005 |
| 0.416669995 | 0.875000000 | 0.193918005 |
| 0.500000000 | 0.000000000 | 0.193918005 |
| 0.083329998 | 0.125000000 | 0.193918005 |
| 0.166669995 | 0.250000000 | 0.193918005 |
| 0.250000000 | 0.375000000 | 0.193918005 |
| 0.333330005 | 0.500000000 | 0.193918005 |
| 0.416669995 | 0.625000000 | 0.193918005 |
| 0.500000000 | 0.750000000 | 0.193918005 |
| 0.583329976 | 0.875000000 | 0.193918005 |
| 0.666670024 | 0.000000000 | 0.193918005 |
| 0.250000000 | 0.125000000 | 0.193918005 |
| 0.333330005 | 0.250000000 | 0.193918005 |
| 0.416669995 | 0.375000000 | 0.193918005 |
| 0.500000000 | 0.500000000 | 0.193918005 |
| 0.583329976 | 0.625000000 | 0.193918005 |
| 0.666670024 | 0.750000000 | 0.193918005 |
| 0.750000000 | 0.875000000 | 0.193918005 |
| 0.833329976 | 0.000000000 | 0.193918005 |
| 0.416669995 | 0.125000000 | 0.193918005 |
| 0.500000000 | 0.250000000 | 0.193918005 |
| 0.583329976 | 0.375000000 | 0.193918005 |
| 0.666670024 | 0.500000000 | 0.193918005 |
| 0.750000000 | 0.625000000 | 0.193918005 |
| 0.833329976 | 0.750000000 | 0.193918005 |
| 0.916670024 | 0.875000000 | 0.193918005 |
| 0.583329976 | 0.125000000 | 0.193918005 |
| 0.666670024 | 0.250000000 | 0.193918005 |
| 0.750000000 | 0.375000000 | 0.193918005 |
| 0.833329976 | 0.500000000 | 0.193918005 |
| 0.916670024 | 0.625000000 | 0.193918005 |
| 0.750000000 | 0.125000000 | 0.193918005 |
| 0.833329976 | 0.250000000 | 0.193918005 |
| 0.916670024 | 0.375000000 | 0.193918005 |
| 0.916670024 | 0.125000000 | 0.193918005 |
| 0.000878126 | 0.915772259 | 0.293342084 |
| 0.998118997 | 0.662988365 | 0.292061269 |
| 0.082941622 | 0.788371444 | 0.291511238 |
| 0.165510565 | 0.916023612 | 0.291767567 |
| 0.999768436 | 0.413351089 | 0.292735249 |
| 0.082485333 | 0.537985325 | 0.292645395 |
| 0.162886158 | 0.663369298 | 0.290085822 |
| 0.247792140 | 0.792292237 | 0.289615214 |
| 0.332631797 | 0.916149378 | 0.291926563 |
| 0.000190681 | 0.164836347 | 0.292305827 |
| 0.083223946 | 0.289028943 | 0.292765796 |

| | | |
|---|---|---|
| 0.165863767 | 0.413359016 | 0.292616576 |
| 0.248630390 | 0.537648857 | 0.291802078 |
| 0.331535965 | 0.663427114 | 0.295721352 |
| 0.416821092 | 0.792427838 | 0.288478166 |
| 0.500568628 | 0.915864706 | 0.291594386 |
| 0.083044805 | 0.039933726 | 0.291915536 |
| 0.166543826 | 0.164348811 | 0.292436570 |
| 0.249807164 | 0.288543612 | 0.291059405 |
| 0.332569599 | 0.413983613 | 0.290667027 |
| 0.416924745 | 0.537972689 | 0.289988607 |
| 0.503889322 | 0.664067924 | 0.288713664 |
| 0.584878325 | 0.790413916 | 0.291244239 |
| 0.666751742 | 0.914996922 | 0.291926473 |
| 0.249802217 | 0.040107317 | 0.292635679 |
| 0.333237857 | 0.163866699 | 0.291718870 |
| 0.417072028 | 0.288643897 | 0.291210949 |
| 0.500696659 | 0.413149744 | 0.291058421 |
| 0.583945692 | 0.538043022 | 0.292371035 |
| 0.668411672 | 0.662999809 | 0.292382300 |
| 0.749034882 | 0.787679672 | 0.291845769 |
| 0.831055403 | 0.915348053 | 0.294060707 |
| 0.416627735 | 0.039679211 | 0.292618722 |
| 0.499948978 | 0.163909405 | 0.292411268 |
| 0.583312452 | 0.288526922 | 0.292682230 |
| 0.666769981 | 0.413265258 | 0.292905182 |
| 0.750097752 | 0.537961483 | 0.292853296 |
| 0.833396852 | 0.662848532 | 0.291856945 |
| 0.915923476 | 0.786295116 | 0.296210557 |
| 0.583227396 | 0.039886910 | 0.292593658 |
| 0.666433096 | 0.164301455 | 0.292015940 |
| 0.749801934 | 0.288996309 | 0.292570233 |
| 0.833074331 | 0.413471788 | 0.292598367 |
| 0.916251302 | 0.537594378 | 0.292896807 |
| 0.749620497 | 0.039360147 | 0.291857541 |
| 0.832785428 | 0.164649397 | 0.292030841 |
| 0.916552305 | 0.288984001 | 0.292246461 |
| 0.916328847 | 0.041181352 | 0.291928023 |
| 0.550299704 | 0.771525621 | 0.422105014 |
| 0.548289716 | 0.701981723 | 0.416595668 |
| 0.478869677 | 0.669344902 | 0.405651450 |
| 0.409360617 | 0.705442011 | 0.400475711 |
| 0.412076831 | 0.775490105 | 0.406533569 |
| 0.481806815 | 0.807844877 | 0.417476326 |
| 0.337161779 | 0.670077085 | 0.386220932 |
| 0.266627997 | 0.701764524 | 0.401155233 |
| 0.309448391 | 0.281900674 | 0.420118183 |
| 0.266120851 | 0.340754390 | 0.421148777 |
| 0.303165913 | 0.402602315 | 0.423250347 |
| 0.383917540 | 0.406304181 | 0.424735337 |
| 0.427090973 | 0.347327471 | 0.423079282 |
| 0.389905751 | 0.285382628 | 0.420750976 |
| 0.420659989 | 0.474971741 | 0.427228183 |
| 0.504365087 | 0.476315588 | 0.424384713 |
| 0.208738178 | 0.729118943 | 0.411963731 |
| 0.571405232 | 0.478927284 | 0.420590311 |
| 0.601076484 | 0.672674775 | 0.420339555 |
| 0.478341639 | 0.615010858 | 0.402044088 |
| 0.358740360 | 0.804340422 | 0.404272437 |
| 0.482408315 | 0.862078249 | 0.421763241 |
| 0.337366492 | 0.617062449 | 0.397031963 |

```
    0.915905118         0.871640801         0.326855719
    0.604632914         0.797012746         0.430152446
    0.203288153         0.338637859         0.419806421
    0.268963397         0.448424816         0.423618406
    0.489993036         0.349563181         0.423413694
    0.424144417         0.239705607         0.419382900
    0.402453095         0.501469851         0.463807404
    0.399431169         0.504932702         0.392781615
    0.280576825         0.233560875         0.418213338
```

5. M.M. in Figure 3.

```
CONTCAR
1.0
      17.3481998444         0.0000000000         0.0000000000
       0.0000000000        20.0319004059         0.0000000000
       0.0000000000         0.0000000000        25.0000000000
   Au    C    N    H
  144   16    2   14
Direct
    0.000000000         0.833329976         0.095569998
    0.083329998         0.958329976         0.095569998
    0.000000000         0.583329976         0.095569998
    0.083329998         0.708329976         0.095569998
    0.166669995         0.833329976         0.095569998
    0.250000000         0.958329976         0.095569998
    0.000000000         0.333330005         0.095569998
    0.083329998         0.458330005         0.095569998
    0.166669995         0.583329976         0.095569998
    0.250000000         0.708329976         0.095569998
    0.333330005         0.833329976         0.095569998
    0.416669995         0.958329976         0.095569998
    0.000000000         0.083329998         0.095569998
    0.083329998         0.208330005         0.095569998
    0.166669995         0.333330005         0.095569998
    0.250000000         0.458330005         0.095569998
    0.333330005         0.583329976         0.095569998
    0.416669995         0.708329976         0.095569998
    0.500000000         0.833329976         0.095569998
    0.583329976         0.958329976         0.095569998
    0.166669995         0.083329998         0.095569998
    0.250000000         0.208330005         0.095569998
    0.333330005         0.333330005         0.095569998
    0.416669995         0.458330005         0.095569998
    0.500000000         0.583329976         0.095569998
    0.583329976         0.708329976         0.095569998
    0.666670024         0.833329976         0.095569998
    0.750000000         0.958329976         0.095569998
    0.333330005         0.083329998         0.095569998
    0.416669995         0.208330005         0.095569998
    0.500000000         0.333330005         0.095569998
    0.583329976         0.458330005         0.095569998
    0.666670024         0.583329976         0.095569998
    0.750000000         0.708329976         0.095569998
    0.833329976         0.833329976         0.095569998
    0.916670024         0.958329976         0.095569998
    0.500000000         0.083329998         0.095569998
    0.583329976         0.208330005         0.095569998
    0.666670024         0.333330005         0.095569998
```

| | | |
|---|---|---|
| 0.750000000 | 0.458330005 | 0.095569998 |
| 0.833329976 | 0.583329976 | 0.095569998 |
| 0.916670024 | 0.708329976 | 0.095569998 |
| 0.666670024 | 0.083329998 | 0.095569998 |
| 0.750000000 | 0.208330005 | 0.095569998 |
| 0.833329976 | 0.333330005 | 0.095569998 |
| 0.916670024 | 0.458330005 | 0.095569998 |
| 0.833329976 | 0.083329998 | 0.095569998 |
| 0.916670024 | 0.208330005 | 0.095569998 |
| 0.000000000 | 0.000000000 | 0.193918005 |
| 0.000000000 | 0.750000000 | 0.193918005 |
| 0.083329998 | 0.875000000 | 0.193918005 |
| 0.166669995 | 0.000000000 | 0.193918005 |
| 0.000000000 | 0.500000000 | 0.193918005 |
| 0.083329998 | 0.625000000 | 0.193918005 |
| 0.166669995 | 0.750000000 | 0.193918005 |
| 0.250000000 | 0.875000000 | 0.193918005 |
| 0.333330005 | 0.000000000 | 0.193918005 |
| 0.000000000 | 0.250000000 | 0.193918005 |
| 0.083329998 | 0.375000000 | 0.193918005 |
| 0.166669995 | 0.500000000 | 0.193918005 |
| 0.250000000 | 0.625000000 | 0.193918005 |
| 0.333330005 | 0.750000000 | 0.193918005 |
| 0.416669995 | 0.875000000 | 0.193918005 |
| 0.500000000 | 0.000000000 | 0.193918005 |
| 0.083329998 | 0.125000000 | 0.193918005 |
| 0.166669995 | 0.250000000 | 0.193918005 |
| 0.250000000 | 0.375000000 | 0.193918005 |
| 0.333330005 | 0.500000000 | 0.193918005 |
| 0.416669995 | 0.625000000 | 0.193918005 |
| 0.500000000 | 0.750000000 | 0.193918005 |
| 0.583329976 | 0.875000000 | 0.193918005 |
| 0.666670024 | 0.000000000 | 0.193918005 |
| 0.250000000 | 0.125000000 | 0.193918005 |
| 0.333330005 | 0.250000000 | 0.193918005 |
| 0.416669995 | 0.375000000 | 0.193918005 |
| 0.500000000 | 0.500000000 | 0.193918005 |
| 0.583329976 | 0.625000000 | 0.193918005 |
| 0.666670024 | 0.750000000 | 0.193918005 |
| 0.750000000 | 0.875000000 | 0.193918005 |
| 0.833329976 | 0.000000000 | 0.193918005 |
| 0.416669995 | 0.125000000 | 0.193918005 |
| 0.500000000 | 0.250000000 | 0.193918005 |
| 0.583329976 | 0.375000000 | 0.193918005 |
| 0.666670024 | 0.500000000 | 0.193918005 |
| 0.750000000 | 0.625000000 | 0.193918005 |
| 0.833329976 | 0.750000000 | 0.193918005 |
| 0.916670024 | 0.875000000 | 0.193918005 |
| 0.583329976 | 0.125000000 | 0.193918005 |
| 0.666670024 | 0.250000000 | 0.193918005 |
| 0.750000000 | 0.375000000 | 0.193918005 |
| 0.833329976 | 0.500000000 | 0.193918005 |
| 0.916670024 | 0.625000000 | 0.193918005 |
| 0.750000000 | 0.125000000 | 0.193918005 |
| 0.833329976 | 0.250000000 | 0.193918005 |
| 0.916670024 | 0.375000000 | 0.193918005 |
| 0.916670024 | 0.125000000 | 0.193918005 |
| 0.999120831 | 0.915458798 | 0.292134821 |
| 0.997954249 | 0.664764762 | 0.293047309 |
| 0.081599288 | 0.790798664 | 0.292173773 |

| | | |
|---|---|---|
| 0.165228695 | 0.916235864 | 0.292421401 |
| 0.999561131 | 0.414687455 | 0.292192847 |
| 0.082166642 | 0.539592862 | 0.293011665 |
| 0.161615714 | 0.665067971 | 0.290278584 |
| 0.247468755 | 0.792807758 | 0.289583892 |
| 0.332596809 | 0.916340828 | 0.292507887 |
| 0.999892652 | 0.164926231 | 0.292578667 |
| 0.082753785 | 0.289279848 | 0.292967916 |
| 0.163870618 | 0.414437026 | 0.292665988 |
| 0.247650757 | 0.539144576 | 0.291802794 |
| 0.329915404 | 0.664521217 | 0.295948118 |
| 0.416926354 | 0.792846859 | 0.288942933 |
| 0.500849068 | 0.916200101 | 0.291825861 |
| 0.082683697 | 0.040330786 | 0.292369187 |
| 0.166229680 | 0.164536387 | 0.292628229 |
| 0.248387665 | 0.288761526 | 0.291410744 |
| 0.329348117 | 0.415203780 | 0.289004892 |
| 0.415843755 | 0.540550530 | 0.289394408 |
| 0.503572285 | 0.665761828 | 0.288919687 |
| 0.585372210 | 0.791575432 | 0.291707724 |
| 0.667545259 | 0.915476263 | 0.292894632 |
| 0.249281168 | 0.039950930 | 0.292675763 |
| 0.332697511 | 0.163311049 | 0.291939080 |
| 0.416613281 | 0.287370890 | 0.288813800 |
| 0.502595127 | 0.417293906 | 0.296124101 |
| 0.585717678 | 0.541662991 | 0.291819006 |
| 0.669257283 | 0.665968120 | 0.292450696 |
| 0.750596702 | 0.790737748 | 0.292962700 |
| 0.833379090 | 0.915154099 | 0.292595446 |
| 0.416640610 | 0.039625101 | 0.292755008 |
| 0.500345409 | 0.163273275 | 0.292457253 |
| 0.584833801 | 0.288014978 | 0.289508432 |
| 0.671054721 | 0.415708125 | 0.290207267 |
| 0.751339257 | 0.540654182 | 0.292997360 |
| 0.833791971 | 0.665247798 | 0.292232573 |
| 0.916213989 | 0.790392756 | 0.292580724 |
| 0.583889902 | 0.039709210 | 0.292578936 |
| 0.667380571 | 0.163879439 | 0.292357534 |
| 0.750809312 | 0.289566278 | 0.292210758 |
| 0.835068047 | 0.415136755 | 0.292746186 |
| 0.916663349 | 0.539825737 | 0.293171406 |
| 0.750392139 | 0.039698012 | 0.292465359 |
| 0.833618283 | 0.164723739 | 0.292183280 |
| 0.916864276 | 0.289534837 | 0.292590290 |
| 0.916530252 | 0.040045932 | 0.292449206 |
| 0.547878325 | 0.770198226 | 0.422803819 |
| 0.545352161 | 0.700699508 | 0.417121440 |
| 0.475717455 | 0.668414056 | 0.406158209 |
| 0.406463295 | 0.704940259 | 0.401023895 |
| 0.409792572 | 0.775017381 | 0.406984299 |
| 0.479703546 | 0.806946039 | 0.418081015 |
| 0.334135264 | 0.670095563 | 0.386837304 |
| 0.263697177 | 0.702220380 | 0.401262760 |
| 0.288907647 | 0.305919647 | 0.422455221 |
| 0.288445413 | 0.375524044 | 0.417411208 |
| 0.356613368 | 0.410211325 | 0.406666905 |
| 0.427320927 | 0.375977933 | 0.400995672 |
| 0.427047640 | 0.305744052 | 0.406416178 |
| 0.358609259 | 0.271442711 | 0.417360514 |
| 0.498121500 | 0.413173169 | 0.386900246 |

| | | |
|---|---|---|
| 0.569815576 | 0.383626640 | 0.401992351 |
| 0.205840200 | 0.729990900 | 0.411487520 |
| 0.628698051 | 0.358055800 | 0.412974417 |
| 0.598097622 | 0.671312988 | 0.420742780 |
| 0.474264205 | 0.614041626 | 0.402558178 |
| 0.356667846 | 0.804151654 | 0.404639661 |
| 0.480719626 | 0.861173391 | 0.422422171 |
| 0.333873808 | 0.616944969 | 0.397265762 |
| 0.916575968 | 0.754647553 | 0.622292519 |
| 0.602390230 | 0.795312524 | 0.430971980 |
| 0.234480172 | 0.403111607 | 0.421440691 |
| 0.355697840 | 0.464635193 | 0.403684705 |
| 0.481396407 | 0.278414488 | 0.403805524 |
| 0.359957933 | 0.217178151 | 0.421293586 |
| 0.495792717 | 0.466334820 | 0.397146076 |
| 0.918272197 | 0.717214048 | 0.621015489 |
| 0.235542133 | 0.278935820 | 0.430467904 |

6. MM: in Figure 3

```
CONTCAR
1.0
      17.3481998444         0.0000000000         0.0000000000
       0.0000000000        20.0319004059         0.0000000000
       0.0000000000         0.0000000000        25.0000000000
   Au    C    N    H
  144   16    2   14
Direct
    0.000000000    0.833329976    0.095569998
    0.083329998    0.958329976    0.095569998
    0.000000000    0.583329976    0.095569998
    0.083329998    0.708329976    0.095569998
    0.166669995    0.833329976    0.095569998
    0.250000000    0.958329976    0.095569998
    0.000000000    0.333330005    0.095569998
    0.083329998    0.458330005    0.095569998
    0.166669995    0.583329976    0.095569998
    0.250000000    0.708329976    0.095569998
    0.333330005    0.833329976    0.095569998
    0.416669995    0.958329976    0.095569998
    0.000000000    0.083329998    0.095569998
    0.083329998    0.208330005    0.095569998
    0.166669995    0.333330005    0.095569998
    0.250000000    0.458330005    0.095569998
    0.333330005    0.583329976    0.095569998
    0.416669995    0.708329976    0.095569998
    0.500000000    0.833329976    0.095569998
    0.583329976    0.958329976    0.095569998
    0.166669995    0.083329998    0.095569998
    0.250000000    0.208330005    0.095569998
    0.333330005    0.333330005    0.095569998
    0.416669995    0.458330005    0.095569998
    0.500000000    0.583329976    0.095569998
    0.583329976    0.708329976    0.095569998
    0.666670024    0.833329976    0.095569998
    0.750000000    0.958329976    0.095569998
    0.333330005    0.083329998    0.095569998
    0.416669995    0.208330005    0.095569998
    0.500000000    0.333330005    0.095569998
```

| | | |
|---|---|---|
| 0.583329976 | 0.458330005 | 0.095569998 |
| 0.666670024 | 0.583329976 | 0.095569998 |
| 0.750000000 | 0.708329976 | 0.095569998 |
| 0.833329976 | 0.833329976 | 0.095569998 |
| 0.916670024 | 0.958329976 | 0.095569998 |
| 0.500000000 | 0.083329998 | 0.095569998 |
| 0.583329976 | 0.208330005 | 0.095569998 |
| 0.666670024 | 0.333330005 | 0.095569998 |
| 0.750000000 | 0.458330005 | 0.095569998 |
| 0.833329976 | 0.583329976 | 0.095569998 |
| 0.916670024 | 0.708329976 | 0.095569998 |
| 0.666670024 | 0.083329998 | 0.095569998 |
| 0.750000000 | 0.208330005 | 0.095569998 |
| 0.833329976 | 0.333330005 | 0.095569998 |
| 0.916670024 | 0.458330005 | 0.095569998 |
| 0.833329976 | 0.083329998 | 0.095569998 |
| 0.916670024 | 0.208330005 | 0.095569998 |
| 0.000000000 | 0.000000000 | 0.193918005 |
| 0.000000000 | 0.750000000 | 0.193918005 |
| 0.083329998 | 0.875000000 | 0.193918005 |
| 0.166669995 | 0.000000000 | 0.193918005 |
| 0.000000000 | 0.500000000 | 0.193918005 |
| 0.083329998 | 0.625000000 | 0.193918005 |
| 0.166669995 | 0.750000000 | 0.193918005 |
| 0.250000000 | 0.875000000 | 0.193918005 |
| 0.333330005 | 0.000000000 | 0.193918005 |
| 0.000000000 | 0.250000000 | 0.193918005 |
| 0.083329998 | 0.375000000 | 0.193918005 |
| 0.166669995 | 0.500000000 | 0.193918005 |
| 0.250000000 | 0.625000000 | 0.193918005 |
| 0.333330005 | 0.750000000 | 0.193918005 |
| 0.416669995 | 0.875000000 | 0.193918005 |
| 0.500000000 | 0.000000000 | 0.193918005 |
| 0.083329998 | 0.125000000 | 0.193918005 |
| 0.166669995 | 0.250000000 | 0.193918005 |
| 0.250000000 | 0.375000000 | 0.193918005 |
| 0.333330005 | 0.500000000 | 0.193918005 |
| 0.416669995 | 0.625000000 | 0.193918005 |
| 0.500000000 | 0.750000000 | 0.193918005 |
| 0.583329976 | 0.875000000 | 0.193918005 |
| 0.666670024 | 0.000000000 | 0.193918005 |
| 0.250000000 | 0.125000000 | 0.193918005 |
| 0.333330005 | 0.250000000 | 0.193918005 |
| 0.416669995 | 0.375000000 | 0.193918005 |
| 0.500000000 | 0.500000000 | 0.193918005 |
| 0.583329976 | 0.625000000 | 0.193918005 |
| 0.666670024 | 0.750000000 | 0.193918005 |
| 0.750000000 | 0.875000000 | 0.193918005 |
| 0.833329976 | 0.000000000 | 0.193918005 |
| 0.416669995 | 0.125000000 | 0.193918005 |
| 0.500000000 | 0.250000000 | 0.193918005 |
| 0.583329976 | 0.375000000 | 0.193918005 |
| 0.666670024 | 0.500000000 | 0.193918005 |
| 0.750000000 | 0.625000000 | 0.193918005 |
| 0.833329976 | 0.750000000 | 0.193918005 |
| 0.916670024 | 0.875000000 | 0.193918005 |
| 0.583329976 | 0.125000000 | 0.193918005 |
| 0.666670024 | 0.250000000 | 0.193918005 |
| 0.750000000 | 0.375000000 | 0.193918005 |
| 0.833329976 | 0.500000000 | 0.193918005 |

| | | |
|---|---|---|
| 0.916670024 | 0.625000000 | 0.193918005 |
| 0.750000000 | 0.125000000 | 0.193918005 |
| 0.833329976 | 0.250000000 | 0.193918005 |
| 0.916670024 | 0.375000000 | 0.193918005 |
| 0.916670024 | 0.125000000 | 0.193918005 |
| 0.000532929 | 0.914556742 | 0.292439163 |
| 0.998842359 | 0.664480388 | 0.292453706 |
| 0.082859039 | 0.790125966 | 0.292874604 |
| 0.166715816 | 0.914758921 | 0.291943520 |
| 0.998910427 | 0.413346916 | 0.293392718 |
| 0.079958960 | 0.538427651 | 0.292666316 |
| 0.162394300 | 0.665012360 | 0.291292787 |
| 0.249683782 | 0.790270984 | 0.291310281 |
| 0.333573192 | 0.915264666 | 0.292146534 |
| 0.000040760 | 0.164304361 | 0.291809678 |
| 0.082588442 | 0.288222641 | 0.292669147 |
| 0.164172173 | 0.411769301 | 0.292713642 |
| 0.242775917 | 0.535303175 | 0.288917392 |
| 0.328533977 | 0.664153278 | 0.291648209 |
| 0.418786943 | 0.792542577 | 0.290601671 |
| 0.501337171 | 0.916518390 | 0.292737961 |
| 0.083302453 | 0.039305888 | 0.292296380 |
| 0.166436642 | 0.163514748 | 0.292451948 |
| 0.249432638 | 0.288370311 | 0.290841758 |
| 0.332716346 | 0.415073305 | 0.290121347 |
| 0.422871917 | 0.541522205 | 0.302998453 |
| 0.510040343 | 0.669921458 | 0.286591470 |
| 0.587619424 | 0.793921888 | 0.292773545 |
| 0.668837190 | 0.916587412 | 0.292411596 |
| 0.250155449 | 0.039557222 | 0.292410254 |
| 0.333592385 | 0.164119035 | 0.291163325 |
| 0.417749584 | 0.290052414 | 0.291221499 |
| 0.498805523 | 0.416497350 | 0.289684951 |
| 0.587882102 | 0.539154410 | 0.290013701 |
| 0.672552407 | 0.666199327 | 0.292617977 |
| 0.752380848 | 0.790992141 | 0.293391794 |
| 0.834587932 | 0.915062368 | 0.292543173 |
| 0.416988581 | 0.039989561 | 0.292199552 |
| 0.500064909 | 0.164839074 | 0.292260051 |
| 0.583338439 | 0.290058106 | 0.291933894 |
| 0.667385876 | 0.414003879 | 0.292835414 |
| 0.751616836 | 0.539361477 | 0.292841494 |
| 0.835298181 | 0.664792895 | 0.292011619 |
| 0.917205155 | 0.789999306 | 0.293132901 |
| 0.583985806 | 0.040691983 | 0.292319298 |
| 0.666767538 | 0.165205076 | 0.292203516 |
| 0.749700248 | 0.289659381 | 0.292413652 |
| 0.833352149 | 0.413998216 | 0.292942941 |
| 0.916023970 | 0.538779378 | 0.292385995 |
| 0.750626385 | 0.040403366 | 0.291964620 |
| 0.833317816 | 0.164864898 | 0.291636020 |
| 0.916286707 | 0.289357513 | 0.292399406 |
| 0.917052031 | 0.039608765 | 0.291990429 |
| 0.538199306 | 0.748633206 | 0.437826067 |
| 0.562256753 | 0.684708595 | 0.423502743 |
| 0.510287285 | 0.639636040 | 0.400282919 |
| 0.433006644 | 0.657999516 | 0.390525043 |
| 0.408969700 | 0.722056508 | 0.407444596 |
| 0.460987985 | 0.766728938 | 0.430370927 |
| 0.377983600 | 0.613191187 | 0.361229122 |

```
0.319788992    0.584027231    0.392993867
0.289373636    0.268542260    0.415809155
0.258159935    0.332877606    0.418401778
0.306443155    0.388457775    0.423109829
0.386669040    0.380035907    0.425499946
0.417910993    0.315683335    0.421885312
0.369394928    0.260179311    0.417266846
0.436387509    0.441763997    0.432980925
0.518248260    0.432193696    0.423011839
0.272211313    0.557693660    0.419470847
0.583843052    0.425307989    0.413927734
0.621982753    0.669339061    0.429612100
0.529335737    0.589198709    0.390252203
0.349063963    0.736795545    0.401144683
0.441222221    0.816531479    0.441606671
0.916550338    0.754601955    0.622356474
0.918308675    0.717177331    0.621103942
0.579086244    0.784219146    0.454738438
0.195818856    0.340009123    0.416718811
0.281821191    0.438654214    0.425403863
0.480350435    0.308731526    0.422896713
0.394371212    0.210269585    0.414614946
0.429072380    0.461787879    0.473802298
0.416532367    0.481547803    0.405613989
0.251597285    0.225172505    0.412456334
```

7. L(C-C) in Figure 3.

```
CONTCAR
1.0
     17.3481998444     0.0000000000     0.0000000000
      0.0000000000    20.0319004059     0.0000000000
      0.0000000000     0.0000000000    25.0000000000
  Au   C   N   H
 144  16   2  14
Direct
0.000000000    0.833329976    0.095569998
0.083329998    0.958329976    0.095569998
0.000000000    0.583329976    0.095569998
0.083329998    0.708329976    0.095569998
0.166669995    0.833329976    0.095569998
0.250000000    0.958329976    0.095569998
0.000000000    0.333330005    0.095569998
0.083329998    0.458330005    0.095569998
0.166669995    0.583329976    0.095569998
0.250000000    0.708329976    0.095569998
0.333330005    0.833329976    0.095569998
0.416669995    0.958329976    0.095569998
0.000000000    0.083329998    0.095569998
0.083329998    0.208330005    0.095569998
0.166669995    0.333330005    0.095569998
0.250000000    0.458330005    0.095569998
0.333330005    0.583329976    0.095569998
0.416669995    0.708329976    0.095569998
0.500000000    0.833329976    0.095569998
0.583329976    0.958329976    0.095569998
0.166669995    0.083329998    0.095569998
0.250000000    0.208330005    0.095569998
0.333330005    0.333330005    0.095569998
```

| | | |
|---|---|---|
| 0.416669995 | 0.458330005 | 0.095569998 |
| 0.500000000 | 0.583329976 | 0.095569998 |
| 0.583329976 | 0.708329976 | 0.095569998 |
| 0.666670024 | 0.833329976 | 0.095569998 |
| 0.750000000 | 0.958329976 | 0.095569998 |
| 0.333330005 | 0.083329998 | 0.095569998 |
| 0.416669995 | 0.208330005 | 0.095569998 |
| 0.500000000 | 0.333330005 | 0.095569998 |
| 0.583329976 | 0.458330005 | 0.095569998 |
| 0.666670024 | 0.583329976 | 0.095569998 |
| 0.750000000 | 0.708329976 | 0.095569998 |
| 0.833329976 | 0.833329976 | 0.095569998 |
| 0.916670024 | 0.958329976 | 0.095569998 |
| 0.500000000 | 0.083329998 | 0.095569998 |
| 0.583329976 | 0.208330005 | 0.095569998 |
| 0.666670024 | 0.333330005 | 0.095569998 |
| 0.750000000 | 0.458330005 | 0.095569998 |
| 0.833329976 | 0.583329976 | 0.095569998 |
| 0.916670024 | 0.708329976 | 0.095569998 |
| 0.666670024 | 0.083329998 | 0.095569998 |
| 0.750000000 | 0.208330005 | 0.095569998 |
| 0.833329976 | 0.333330005 | 0.095569998 |
| 0.916670024 | 0.458330005 | 0.095569998 |
| 0.833329976 | 0.083329998 | 0.095569998 |
| 0.916670024 | 0.208330005 | 0.095569998 |
| 0.000000000 | 0.000000000 | 0.193918005 |
| 0.000000000 | 0.750000000 | 0.193918005 |
| 0.083329998 | 0.875000000 | 0.193918005 |
| 0.166669995 | 0.000000000 | 0.193918005 |
| 0.000000000 | 0.500000000 | 0.193918005 |
| 0.083329998 | 0.625000000 | 0.193918005 |
| 0.166669995 | 0.750000000 | 0.193918005 |
| 0.250000000 | 0.875000000 | 0.193918005 |
| 0.333330005 | 0.000000000 | 0.193918005 |
| 0.000000000 | 0.250000000 | 0.193918005 |
| 0.083329998 | 0.375000000 | 0.193918005 |
| 0.166669995 | 0.500000000 | 0.193918005 |
| 0.250000000 | 0.625000000 | 0.193918005 |
| 0.333330005 | 0.750000000 | 0.193918005 |
| 0.416669995 | 0.875000000 | 0.193918005 |
| 0.500000000 | 0.000000000 | 0.193918005 |
| 0.083329998 | 0.125000000 | 0.193918005 |
| 0.166669995 | 0.250000000 | 0.193918005 |
| 0.250000000 | 0.375000000 | 0.193918005 |
| 0.333330005 | 0.500000000 | 0.193918005 |
| 0.416669995 | 0.625000000 | 0.193918005 |
| 0.500000000 | 0.750000000 | 0.193918005 |
| 0.583329976 | 0.875000000 | 0.193918005 |
| 0.666670024 | 0.000000000 | 0.193918005 |
| 0.250000000 | 0.125000000 | 0.193918005 |
| 0.333330005 | 0.250000000 | 0.193918005 |
| 0.416669995 | 0.375000000 | 0.193918005 |
| 0.500000000 | 0.500000000 | 0.193918005 |
| 0.583329976 | 0.625000000 | 0.193918005 |
| 0.666670024 | 0.750000000 | 0.193918005 |
| 0.750000000 | 0.875000000 | 0.193918005 |
| 0.833329976 | 0.000000000 | 0.193918005 |
| 0.416669995 | 0.125000000 | 0.193918005 |
| 0.500000000 | 0.250000000 | 0.193918005 |
| 0.583329976 | 0.375000000 | 0.193918005 |

| | | |
|---|---|---|
| 0.666670024 | 0.500000000 | 0.193918005 |
| 0.750000000 | 0.625000000 | 0.193918005 |
| 0.833329976 | 0.750000000 | 0.193918005 |
| 0.916670024 | 0.875000000 | 0.193918005 |
| 0.583329976 | 0.125000000 | 0.193918005 |
| 0.666670024 | 0.250000000 | 0.193918005 |
| 0.750000000 | 0.375000000 | 0.193918005 |
| 0.833329976 | 0.500000000 | 0.193918005 |
| 0.916670024 | 0.625000000 | 0.193918005 |
| 0.750000000 | 0.125000000 | 0.193918005 |
| 0.833329976 | 0.250000000 | 0.193918005 |
| 0.916670024 | 0.375000000 | 0.193918005 |
| 0.916670024 | 0.125000000 | 0.193918005 |
| 0.000054389 | 0.914853215 | 0.291899949 |
| 0.002239489 | 0.662901878 | 0.293396652 |
| 0.084452093 | 0.790568888 | 0.290555626 |
| 0.167221829 | 0.915052354 | 0.292143345 |
| 0.000371599 | 0.414763033 | 0.292450458 |
| 0.083947048 | 0.539306462 | 0.292085499 |
| 0.166975245 | 0.664592624 | 0.290903598 |
| 0.250222385 | 0.790249348 | 0.292806715 |
| 0.333460689 | 0.915017545 | 0.292919755 |
| 0.002519972 | 0.162887931 | 0.293689579 |
| 0.084648438 | 0.290258378 | 0.290644109 |
| 0.166967347 | 0.414989769 | 0.292139947 |
| 0.250586987 | 0.538948417 | 0.290514261 |
| 0.333640486 | 0.665806770 | 0.289148271 |
| 0.416237593 | 0.790619969 | 0.291082680 |
| 0.499376148 | 0.914821506 | 0.292729855 |
| 0.083886705 | 0.039463356 | 0.291893274 |
| 0.167459041 | 0.164047867 | 0.292033017 |
| 0.250455588 | 0.289216727 | 0.291036218 |
| 0.333932102 | 0.414646357 | 0.292355508 |
| 0.416533142 | 0.539226770 | 0.292070448 |
| 0.499899358 | 0.664561510 | 0.290636063 |
| 0.582712114 | 0.789926827 | 0.291890323 |
| 0.665577710 | 0.914868414 | 0.292544574 |
| 0.250579089 | 0.039554872 | 0.292230844 |
| 0.333544850 | 0.164642930 | 0.291571110 |
| 0.416689545 | 0.289652050 | 0.291544408 |
| 0.499342889 | 0.414668053 | 0.293280214 |
| 0.582274675 | 0.539139032 | 0.292699575 |
| 0.665572107 | 0.663935781 | 0.291874260 |
| 0.748200119 | 0.790103316 | 0.290684611 |
| 0.832604408 | 0.914633274 | 0.291901380 |
| 0.416549653 | 0.039524313 | 0.292185694 |
| 0.499695212 | 0.164746016 | 0.291425139 |
| 0.582629442 | 0.289836913 | 0.292538643 |
| 0.665701985 | 0.414885402 | 0.292045265 |
| 0.748946726 | 0.539409816 | 0.291990399 |
| 0.830243945 | 0.662794888 | 0.293967783 |
| 0.916417360 | 0.792646527 | 0.295807004 |
| 0.582433820 | 0.039418858 | 0.292284489 |
| 0.665508509 | 0.164153025 | 0.291758180 |
| 0.748530567 | 0.290286273 | 0.290536672 |
| 0.832915306 | 0.414888978 | 0.291845143 |
| 0.916721761 | 0.538397431 | 0.291374922 |
| 0.748985112 | 0.039340954 | 0.291982919 |
| 0.830375135 | 0.162939698 | 0.293614626 |
| 0.916704535 | 0.292858213 | 0.295998871 |

| | | |
|---|---|---|
| 0.916384876 | 0.038427554 | 0.291366607 |
| 0.489584804 | 0.765102327 | 0.423618406 |
| 0.515836418 | 0.699534178 | 0.417427003 |
| 0.463675857 | 0.646412015 | 0.417605102 |
| 0.384588480 | 0.658140957 | 0.425289243 |
| 0.358735859 | 0.723963857 | 0.431351751 |
| 0.410916895 | 0.777167499 | 0.430212855 |
| 0.328913003 | 0.599327803 | 0.422690004 |
| 0.249948159 | 0.621892631 | 0.414203942 |
| 0.209735245 | 0.367661387 | 0.421568871 |
| 0.167898893 | 0.422574222 | 0.440054536 |
| 0.206320688 | 0.480060488 | 0.457115978 |
| 0.286923438 | 0.483679682 | 0.454714686 |
| 0.328607291 | 0.428880036 | 0.435213298 |
| 0.290184408 | 0.370925158 | 0.419339955 |
| 0.327981532 | 0.548042893 | 0.471150696 |
| 0.405749977 | 0.534425855 | 0.490564406 |
| 0.186893493 | 0.640894711 | 0.408051372 |
| 0.467691809 | 0.522103250 | 0.505847037 |
| 0.577111602 | 0.689533830 | 0.411648721 |
| 0.484912455 | 0.595685601 | 0.411698103 |
| 0.297259122 | 0.734173477 | 0.435846955 |
| 0.389624029 | 0.828207016 | 0.434062600 |
| 0.344632506 | 0.569445491 | 0.386883050 |
| 0.916511655 | 0.707321525 | 0.325033963 |
| 0.530266643 | 0.806635737 | 0.422425330 |
| 0.105072953 | 0.420589626 | 0.441619307 |
| 0.173107237 | 0.522413135 | 0.472076893 |
| 0.391453177 | 0.430895418 | 0.433155715 |
| 0.323338240 | 0.328109920 | 0.405157238 |
| 0.296353161 | 0.571803629 | 0.504364073 |
| 0.916477621 | 0.207494125 | 0.324949890 |
| 0.179853007 | 0.322358966 | 0.408938259 |

8. L(C=C): in Figure 3

CONTCAR
1.0

| | | |
|---|---|---|
| 17.3481998444 | 0.0000000000 | 0.0000000000 |
| 0.0000000000 | 20.0319004059 | 0.0000000000 |
| 0.0000000000 | 0.0000000000 | 25.0000000000 |

| | | | |
|---|---|---|---|
| Au | C | N | H |
| 144 | 16 | 2 | 14 |

Direct

| | | |
|---|---|---|
| 0.000000000 | 0.833329976 | 0.095569998 |
| 0.083329998 | 0.958329976 | 0.095569998 |
| 0.000000000 | 0.583329976 | 0.095569998 |
| 0.083329998 | 0.708329976 | 0.095569998 |
| 0.166669995 | 0.833329976 | 0.095569998 |
| 0.250000000 | 0.958329976 | 0.095569998 |
| 0.000000000 | 0.333330005 | 0.095569998 |
| 0.083329998 | 0.458330005 | 0.095569998 |
| 0.166669995 | 0.583329976 | 0.095569998 |
| 0.250000000 | 0.708329976 | 0.095569998 |
| 0.333330005 | 0.833329976 | 0.095569998 |
| 0.416669995 | 0.958329976 | 0.095569998 |
| 0.000000000 | 0.083329998 | 0.095569998 |
| 0.083329998 | 0.208330005 | 0.095569998 |
| 0.166669995 | 0.333330005 | 0.095569998 |

| | | |
|---|---|---|
| 0.250000000 | 0.458330005 | 0.095569998 |
| 0.333330005 | 0.583329976 | 0.095569998 |
| 0.416669995 | 0.708329976 | 0.095569998 |
| 0.500000000 | 0.833329976 | 0.095569998 |
| 0.583329976 | 0.958329976 | 0.095569998 |
| 0.166669995 | 0.083329998 | 0.095569998 |
| 0.250000000 | 0.208330005 | 0.095569998 |
| 0.333330005 | 0.333330005 | 0.095569998 |
| 0.416669995 | 0.458330005 | 0.095569998 |
| 0.500000000 | 0.583329976 | 0.095569998 |
| 0.583329976 | 0.708329976 | 0.095569998 |
| 0.666670024 | 0.833329976 | 0.095569998 |
| 0.750000000 | 0.958329976 | 0.095569998 |
| 0.333330005 | 0.083329998 | 0.095569998 |
| 0.416669995 | 0.208330005 | 0.095569998 |
| 0.500000000 | 0.333330005 | 0.095569998 |
| 0.583329976 | 0.458330005 | 0.095569998 |
| 0.666670024 | 0.583329976 | 0.095569998 |
| 0.750000000 | 0.708329976 | 0.095569998 |
| 0.833329976 | 0.833329976 | 0.095569998 |
| 0.916670024 | 0.958329976 | 0.095569998 |
| 0.500000000 | 0.083329998 | 0.095569998 |
| 0.583329976 | 0.208330005 | 0.095569998 |
| 0.666670024 | 0.333330005 | 0.095569998 |
| 0.750000000 | 0.458330005 | 0.095569998 |
| 0.833329976 | 0.583329976 | 0.095569998 |
| 0.916670024 | 0.708329976 | 0.095569998 |
| 0.666670024 | 0.083329998 | 0.095569998 |
| 0.750000000 | 0.208330005 | 0.095569998 |
| 0.833329976 | 0.333330005 | 0.095569998 |
| 0.916670024 | 0.458330005 | 0.095569998 |
| 0.833329976 | 0.083329998 | 0.095569998 |
| 0.916670024 | 0.208330005 | 0.095569998 |
| 0.000000000 | 0.000000000 | 0.193918005 |
| 0.000000000 | 0.750000000 | 0.193918005 |
| 0.083329998 | 0.875000000 | 0.193918005 |
| 0.166669995 | 0.000000000 | 0.193918005 |
| 0.000000000 | 0.500000000 | 0.193918005 |
| 0.083329998 | 0.625000000 | 0.193918005 |
| 0.166669995 | 0.750000000 | 0.193918005 |
| 0.250000000 | 0.875000000 | 0.193918005 |
| 0.333330005 | 0.000000000 | 0.193918005 |
| 0.000000000 | 0.250000000 | 0.193918005 |
| 0.083329998 | 0.375000000 | 0.193918005 |
| 0.166669995 | 0.500000000 | 0.193918005 |
| 0.250000000 | 0.625000000 | 0.193918005 |
| 0.333330005 | 0.750000000 | 0.193918005 |
| 0.416669995 | 0.875000000 | 0.193918005 |
| 0.500000000 | 0.000000000 | 0.193918005 |
| 0.083329998 | 0.125000000 | 0.193918005 |
| 0.166669995 | 0.250000000 | 0.193918005 |
| 0.250000000 | 0.375000000 | 0.193918005 |
| 0.333330005 | 0.500000000 | 0.193918005 |
| 0.416669995 | 0.625000000 | 0.193918005 |
| 0.500000000 | 0.750000000 | 0.193918005 |
| 0.583329976 | 0.875000000 | 0.193918005 |
| 0.666670024 | 0.000000000 | 0.193918005 |
| 0.250000000 | 0.125000000 | 0.193918005 |
| 0.333330005 | 0.250000000 | 0.193918005 |
| 0.416669995 | 0.375000000 | 0.193918005 |

| | | |
|---|---|---|
| 0.500000000 | 0.500000000 | 0.193918005 |
| 0.583329976 | 0.625000000 | 0.193918005 |
| 0.666670024 | 0.750000000 | 0.193918005 |
| 0.750000000 | 0.875000000 | 0.193918005 |
| 0.833329976 | 0.000000000 | 0.193918005 |
| 0.416669995 | 0.125000000 | 0.193918005 |
| 0.500000000 | 0.250000000 | 0.193918005 |
| 0.583329976 | 0.375000000 | 0.193918005 |
| 0.666670024 | 0.500000000 | 0.193918005 |
| 0.750000000 | 0.625000000 | 0.193918005 |
| 0.833329976 | 0.750000000 | 0.193918005 |
| 0.916670024 | 0.875000000 | 0.193918005 |
| 0.583329976 | 0.125000000 | 0.193918005 |
| 0.666670024 | 0.250000000 | 0.193918005 |
| 0.750000000 | 0.375000000 | 0.193918005 |
| 0.833329976 | 0.500000000 | 0.193918005 |
| 0.916670024 | 0.625000000 | 0.193918005 |
| 0.750000000 | 0.125000000 | 0.193918005 |
| 0.833329976 | 0.250000000 | 0.193918005 |
| 0.916670024 | 0.375000000 | 0.193918005 |
| 0.916670024 | 0.125000000 | 0.193918005 |
| 0.000056688 | 0.914637208 | 0.291823328 |
| 0.999694824 | 0.664726317 | 0.292076170 |
| 0.083274670 | 0.789558351 | 0.292484790 |
| 0.166729093 | 0.914625227 | 0.292110682 |
| 0.999752462 | 0.414750993 | 0.292121410 |
| 0.082988858 | 0.539946675 | 0.292234033 |
| 0.165954933 | 0.664767563 | 0.292367280 |
| 0.249512017 | 0.789672434 | 0.292161494 |
| 0.333136499 | 0.914812028 | 0.292057514 |
| 0.000151310 | 0.164672509 | 0.292103499 |
| 0.083257951 | 0.289592385 | 0.292004526 |
| 0.166153461 | 0.415094882 | 0.291045189 |
| 0.249792203 | 0.539607704 | 0.290678889 |
| 0.332350552 | 0.664842486 | 0.290467620 |
| 0.416814327 | 0.790303528 | 0.290934116 |
| 0.500131726 | 0.914805889 | 0.292199522 |
| 0.083431587 | 0.039687499 | 0.292015076 |
| 0.166740119 | 0.164728060 | 0.292291880 |
| 0.250099987 | 0.289376259 | 0.291101247 |
| 0.334304422 | 0.414695501 | 0.290923178 |
| 0.416663349 | 0.540181577 | 0.290826648 |
| 0.499993294 | 0.664690852 | 0.291442066 |
| 0.583589554 | 0.789692998 | 0.292273372 |
| 0.666654050 | 0.914608181 | 0.292013437 |
| 0.250016034 | 0.039819121 | 0.292105526 |
| 0.333453894 | 0.164767981 | 0.292262793 |
| 0.416889936 | 0.289714307 | 0.292446434 |
| 0.500289500 | 0.414916575 | 0.292346627 |
| 0.583376884 | 0.539862037 | 0.292265356 |
| 0.666444302 | 0.664671421 | 0.292357594 |
| 0.749958277 | 0.789460361 | 0.292243958 |
| 0.833315253 | 0.914612532 | 0.291871637 |
| 0.416654408 | 0.039738961 | 0.292088240 |
| 0.499908686 | 0.164818227 | 0.292111129 |
| 0.583271861 | 0.289777994 | 0.292299330 |
| 0.666627705 | 0.414784938 | 0.291804522 |
| 0.749921799 | 0.539727807 | 0.292057961 |
| 0.833122611 | 0.664568305 | 0.291980714 |
| 0.916609228 | 0.789570153 | 0.292231172 |

| | | |
|---|---|---|
| 0.583289564 | 0.039744381 | 0.292036623 |
| 0.666571200 | 0.164664209 | 0.291919351 |
| 0.750024974 | 0.289528489 | 0.292021096 |
| 0.833312094 | 0.414607316 | 0.291930109 |
| 0.916613936 | 0.539689183 | 0.292101115 |
| 0.749941230 | 0.039657876 | 0.292058676 |
| 0.833372295 | 0.164573491 | 0.291975856 |
| 0.916785479 | 0.289583087 | 0.292386711 |
| 0.916703582 | 0.039711110 | 0.292061448 |
| 0.434198171 | 0.770457625 | 0.420619726 |
| 0.474702090 | 0.710096478 | 0.421142966 |
| 0.435996205 | 0.649175942 | 0.423088849 |
| 0.354514450 | 0.646399200 | 0.424663872 |
| 0.314700931 | 0.708202720 | 0.423891395 |
| 0.353770196 | 0.768927038 | 0.422057360 |
| 0.308399141 | 0.583762586 | 0.425831527 |
| 0.208133236 | 0.330645889 | 0.420474768 |
| 0.167574897 | 0.390994757 | 0.419687539 |
| 0.206158921 | 0.452020884 | 0.421543658 |
| 0.287532747 | 0.454905599 | 0.424212754 |
| 0.327419341 | 0.393142283 | 0.424072206 |
| 0.288477421 | 0.332306236 | 0.422482550 |
| 0.333530515 | 0.517581522 | 0.425506830 |
| 0.414550215 | 0.504595041 | 0.425312817 |
| 0.227377921 | 0.596735656 | 0.426096559 |
| 0.479729623 | 0.489174396 | 0.424761117 |
| 0.162155971 | 0.612017930 | 0.425724745 |
| 0.918330252 | 0.217234656 | 0.620965600 |
| 0.918332160 | 0.717233539 | 0.620926321 |
| 0.537574828 | 0.710096896 | 0.419584721 |
| 0.470546871 | 0.604083180 | 0.423108578 |
| 0.251911879 | 0.708990693 | 0.424499899 |
| 0.320752978 | 0.815278351 | 0.421075076 |
| 0.916549742 | 0.754649758 | 0.622303784 |
| 0.464977980 | 0.817915857 | 0.418864757 |
| 0.104751207 | 0.390921146 | 0.417410046 |
| 0.171550408 | 0.497074455 | 0.420560122 |
| 0.390184224 | 0.392396599 | 0.425374150 |
| 0.321636349 | 0.286012352 | 0.422247797 |
| 0.916567624 | 0.254656851 | 0.622262299 |
| 0.177428007 | 0.283152461 | 0.418822527 |

9. L(C-C)* in Figure 3.

CONTCAR
1.0

| | | |
|---|---|---|
| 17.3481998444 | 0.0000000000 | 0.0000000000 |
| 0.0000000000 | 20.0319004059 | 0.0000000000 |
| 0.0000000000 | 0.0000000000 | 25.0000000000 |

| | | | |
|---|---|---|---|
| Au | C | N | H |
| 144 | 16 | 2 | 14 |

Direct

| | | |
|---|---|---|
| 0.000000000 | 0.833329976 | 0.095569998 |
| 0.083329998 | 0.958329976 | 0.095569998 |
| 0.000000000 | 0.583329976 | 0.095569998 |
| 0.083329998 | 0.708329976 | 0.095569998 |
| 0.166669995 | 0.833329976 | 0.095569998 |
| 0.250000000 | 0.958329976 | 0.095569998 |
| 0.000000000 | 0.333330005 | 0.095569998 |

| | | |
|---|---|---|
| 0.083329998 | 0.458330005 | 0.095569998 |
| 0.166669995 | 0.583329976 | 0.095569998 |
| 0.250000000 | 0.708329976 | 0.095569998 |
| 0.333330005 | 0.833329976 | 0.095569998 |
| 0.416669995 | 0.958329976 | 0.095569998 |
| 0.000000000 | 0.083329998 | 0.095569998 |
| 0.083329998 | 0.208330005 | 0.095569998 |
| 0.166669995 | 0.333330005 | 0.095569998 |
| 0.250000000 | 0.458330005 | 0.095569998 |
| 0.333330005 | 0.583329976 | 0.095569998 |
| 0.416669995 | 0.708329976 | 0.095569998 |
| 0.500000000 | 0.833329976 | 0.095569998 |
| 0.583329976 | 0.958329976 | 0.095569998 |
| 0.166669995 | 0.083329998 | 0.095569998 |
| 0.250000000 | 0.208330005 | 0.095569998 |
| 0.333330005 | 0.333330005 | 0.095569998 |
| 0.416669995 | 0.458330005 | 0.095569998 |
| 0.500000000 | 0.583329976 | 0.095569998 |
| 0.583329976 | 0.708329976 | 0.095569998 |
| 0.666670024 | 0.833329976 | 0.095569998 |
| 0.750000000 | 0.958329976 | 0.095569998 |
| 0.333330005 | 0.083329998 | 0.095569998 |
| 0.416669995 | 0.208330005 | 0.095569998 |
| 0.500000000 | 0.333330005 | 0.095569998 |
| 0.583329976 | 0.458330005 | 0.095569998 |
| 0.666670024 | 0.583329976 | 0.095569998 |
| 0.750000000 | 0.708329976 | 0.095569998 |
| 0.833329976 | 0.833329976 | 0.095569998 |
| 0.916670024 | 0.958329976 | 0.095569998 |
| 0.500000000 | 0.083329998 | 0.095569998 |
| 0.583329976 | 0.208330005 | 0.095569998 |
| 0.666670024 | 0.333330005 | 0.095569998 |
| 0.750000000 | 0.458330005 | 0.095569998 |
| 0.833329976 | 0.583329976 | 0.095569998 |
| 0.916670024 | 0.708329976 | 0.095569998 |
| 0.666670024 | 0.083329998 | 0.095569998 |
| 0.750000000 | 0.208330005 | 0.095569998 |
| 0.833329976 | 0.333330005 | 0.095569998 |
| 0.916670024 | 0.458330005 | 0.095569998 |
| 0.833329976 | 0.083329998 | 0.095569998 |
| 0.916670024 | 0.208330005 | 0.095569998 |
| 0.000000000 | 0.000000000 | 0.193918005 |
| 0.000000000 | 0.750000000 | 0.193918005 |
| 0.083329998 | 0.875000000 | 0.193918005 |
| 0.166669995 | 0.000000000 | 0.193918005 |
| 0.000000000 | 0.500000000 | 0.193918005 |
| 0.083329998 | 0.625000000 | 0.193918005 |
| 0.166669995 | 0.750000000 | 0.193918005 |
| 0.250000000 | 0.875000000 | 0.193918005 |
| 0.333330005 | 0.000000000 | 0.193918005 |
| 0.000000000 | 0.250000000 | 0.193918005 |
| 0.083329998 | 0.375000000 | 0.193918005 |
| 0.166669995 | 0.500000000 | 0.193918005 |
| 0.250000000 | 0.625000000 | 0.193918005 |
| 0.333330005 | 0.750000000 | 0.193918005 |
| 0.416669995 | 0.875000000 | 0.193918005 |
| 0.500000000 | 0.000000000 | 0.193918005 |
| 0.083329998 | 0.125000000 | 0.193918005 |
| 0.166669995 | 0.250000000 | 0.193918005 |
| 0.250000000 | 0.375000000 | 0.193918005 |

| | | |
|---|---|---|
| 0.333330005 | 0.500000000 | 0.193918005 |
| 0.416669995 | 0.625000000 | 0.193918005 |
| 0.500000000 | 0.750000000 | 0.193918005 |
| 0.583329976 | 0.875000000 | 0.193918005 |
| 0.666670024 | 0.000000000 | 0.193918005 |
| 0.250000000 | 0.125000000 | 0.193918005 |
| 0.333330005 | 0.250000000 | 0.193918005 |
| 0.416669995 | 0.375000000 | 0.193918005 |
| 0.500000000 | 0.500000000 | 0.193918005 |
| 0.583329976 | 0.625000000 | 0.193918005 |
| 0.666670024 | 0.750000000 | 0.193918005 |
| 0.750000000 | 0.875000000 | 0.193918005 |
| 0.833329976 | 0.000000000 | 0.193918005 |
| 0.416669995 | 0.125000000 | 0.193918005 |
| 0.500000000 | 0.250000000 | 0.193918005 |
| 0.583329976 | 0.375000000 | 0.193918005 |
| 0.666670024 | 0.500000000 | 0.193918005 |
| 0.750000000 | 0.625000000 | 0.193918005 |
| 0.833329976 | 0.750000000 | 0.193918005 |
| 0.916670024 | 0.875000000 | 0.193918005 |
| 0.583329976 | 0.125000000 | 0.193918005 |
| 0.666670024 | 0.250000000 | 0.193918005 |
| 0.750000000 | 0.375000000 | 0.193918005 |
| 0.833329976 | 0.500000000 | 0.193918005 |
| 0.916670024 | 0.625000000 | 0.193918005 |
| 0.750000000 | 0.125000000 | 0.193918005 |
| 0.833329976 | 0.250000000 | 0.193918005 |
| 0.916670024 | 0.375000000 | 0.193918005 |
| 0.916670024 | 0.125000000 | 0.193918005 |
| 0.999769926 | 0.915375471 | 0.292147577 |
| 0.997921824 | 0.666263998 | 0.292531729 |
| 0.081821017 | 0.792013168 | 0.292462200 |
| 0.166536033 | 0.915907621 | 0.292404205 |
| 0.998444438 | 0.416067421 | 0.292241693 |
| 0.080161922 | 0.541201532 | 0.289730728 |
| 0.163846150 | 0.667871654 | 0.288191050 |
| 0.250353754 | 0.792759717 | 0.291074187 |
| 0.333809078 | 0.916312516 | 0.292490989 |
| 0.002689903 | 0.163629085 | 0.294255823 |
| 0.083659813 | 0.290587813 | 0.290005803 |
| 0.165067926 | 0.415026307 | 0.290098637 |
| 0.251322865 | 0.537623882 | 0.300166100 |
| 0.335566461 | 0.667414784 | 0.288500130 |
| 0.417897999 | 0.792061031 | 0.292050898 |
| 0.500669956 | 0.915901303 | 0.292179555 |
| 0.083775766 | 0.040073499 | 0.292062074 |
| 0.167384923 | 0.164063424 | 0.291783273 |
| 0.250370771 | 0.288781792 | 0.290871024 |
| 0.334536254 | 0.412908196 | 0.289926201 |
| 0.418898553 | 0.540644765 | 0.289660066 |
| 0.501604319 | 0.666095734 | 0.292014271 |
| 0.583705723 | 0.790592492 | 0.292715847 |
| 0.666697800 | 0.914982200 | 0.292129129 |
| 0.250687242 | 0.040006477 | 0.292317182 |
| 0.333951831 | 0.164763615 | 0.291916162 |
| 0.417681009 | 0.289210081 | 0.291891664 |
| 0.500198662 | 0.414700657 | 0.292091429 |
| 0.584282279 | 0.540583789 | 0.291987181 |
| 0.667017400 | 0.665614128 | 0.292883068 |
| 0.749866843 | 0.789959192 | 0.293303579 |

| | | |
|---|---|---|
| 0.832471788 | 0.914755106 | 0.291124701 |
| 0.417217046 | 0.040358383 | 0.292306304 |
| 0.500180960 | 0.165329784 | 0.291749209 |
| 0.583436251 | 0.290452868 | 0.293056369 |
| 0.666127264 | 0.415798783 | 0.292650551 |
| 0.749707162 | 0.540458798 | 0.291784286 |
| 0.832303226 | 0.665572345 | 0.292958021 |
| 0.915829420 | 0.790350556 | 0.292690903 |
| 0.583092809 | 0.040167894 | 0.292408586 |
| 0.665994346 | 0.164996937 | 0.291503698 |
| 0.748727322 | 0.291439056 | 0.290622383 |
| 0.832842171 | 0.415970862 | 0.292423338 |
| 0.915271699 | 0.541179240 | 0.293242931 |
| 0.749355912 | 0.040117115 | 0.291954011 |
| 0.830824971 | 0.163894206 | 0.293525249 |
| 0.916397452 | 0.293833286 | 0.297473222 |
| 0.916544855 | 0.039044257 | 0.290961593 |
| 0.254407793 | 0.759133279 | 0.421231687 |
| 0.320844382 | 0.719958961 | 0.416540653 |
| 0.315668792 | 0.651583135 | 0.405763268 |
| 0.243116066 | 0.620122850 | 0.400174946 |
| 0.176619425 | 0.660534918 | 0.405258745 |
| 0.182181552 | 0.728953063 | 0.415833443 |
| 0.233066827 | 0.548309088 | 0.386894166 |
| 0.879588842 | 0.904118836 | 0.422089577 |
| 0.182322100 | 0.295558602 | 0.419020325 |
| 0.133294687 | 0.350803882 | 0.421816975 |
| 0.163686603 | 0.415373236 | 0.422236592 |
| 0.243674025 | 0.425917804 | 0.419642955 |
| 0.292371899 | 0.370299697 | 0.416651309 |
| 0.261913091 | 0.305563658 | 0.416302174 |
| 0.275488526 | 0.497134596 | 0.422775954 |
| 0.359180719 | 0.498405308 | 0.416631013 |
| 0.917989731 | 0.950621486 | 0.430345207 |
| 0.426162362 | 0.499493629 | 0.411660910 |
| 0.377660394 | 0.742918968 | 0.420265108 |
| 0.368492693 | 0.622371554 | 0.402377427 |
| 0.119735911 | 0.637342989 | 0.402367026 |
| 0.129698932 | 0.758725107 | 0.419303298 |
| 0.171515465 | 0.536243975 | 0.389240980 |
| 0.844205201 | 0.861044824 | 0.414202750 |
| 0.258922219 | 0.812586188 | 0.428883195 |
| 0.070940740 | 0.343861043 | 0.423441231 |
| 0.124108255 | 0.457594335 | 0.425138086 |
| 0.354740709 | 0.377163589 | 0.414669305 |
| 0.300909847 | 0.262908369 | 0.414062411 |
| 0.264895916 | 0.514270902 | 0.464436710 |
| 0.916469157 | 0.208082601 | 0.325522214 |
| 0.158612460 | 0.245112777 | 0.418472618 |

10. L(C=C) in Figure 3.

```
CONTCAR
1.0
      17.3481998444         0.0000000000         0.0000000000
       0.0000000000        20.0319004059         0.0000000000
       0.0000000000         0.0000000000        25.0000000000
   Au    C    N    H
  144   16    2   14
```

Direct

| | | |
|---|---|---|
| 0.000000000 | 0.833329976 | 0.095569998 |
| 0.083329998 | 0.958329976 | 0.095569998 |
| 0.000000000 | 0.583329976 | 0.095569998 |
| 0.083329998 | 0.708329976 | 0.095569998 |
| 0.166669995 | 0.833329976 | 0.095569998 |
| 0.250000000 | 0.958329976 | 0.095569998 |
| 0.000000000 | 0.333330005 | 0.095569998 |
| 0.083329998 | 0.458330005 | 0.095569998 |
| 0.166669995 | 0.583329976 | 0.095569998 |
| 0.250000000 | 0.708329976 | 0.095569998 |
| 0.333330005 | 0.833329976 | 0.095569998 |
| 0.416669995 | 0.958329976 | 0.095569998 |
| 0.000000000 | 0.083329998 | 0.095569998 |
| 0.083329998 | 0.208330005 | 0.095569998 |
| 0.166669995 | 0.333330005 | 0.095569998 |
| 0.250000000 | 0.458330005 | 0.095569998 |
| 0.333330005 | 0.583329976 | 0.095569998 |
| 0.416669995 | 0.708329976 | 0.095569998 |
| 0.500000000 | 0.833329976 | 0.095569998 |
| 0.583329976 | 0.958329976 | 0.095569998 |
| 0.166669995 | 0.083329998 | 0.095569998 |
| 0.250000000 | 0.208330005 | 0.095569998 |
| 0.333330005 | 0.333330005 | 0.095569998 |
| 0.416669995 | 0.458330005 | 0.095569998 |
| 0.500000000 | 0.583329976 | 0.095569998 |
| 0.583329976 | 0.708329976 | 0.095569998 |
| 0.666670024 | 0.833329976 | 0.095569998 |
| 0.750000000 | 0.958329976 | 0.095569998 |
| 0.333330005 | 0.083329998 | 0.095569998 |
| 0.416669995 | 0.208330005 | 0.095569998 |
| 0.500000000 | 0.333330005 | 0.095569998 |
| 0.583329976 | 0.458330005 | 0.095569998 |
| 0.666670024 | 0.583329976 | 0.095569998 |
| 0.750000000 | 0.708329976 | 0.095569998 |
| 0.833329976 | 0.833329976 | 0.095569998 |
| 0.916670024 | 0.958329976 | 0.095569998 |
| 0.500000000 | 0.083329998 | 0.095569998 |
| 0.583329976 | 0.208330005 | 0.095569998 |
| 0.666670024 | 0.333330005 | 0.095569998 |
| 0.750000000 | 0.458330005 | 0.095569998 |
| 0.833329976 | 0.583329976 | 0.095569998 |
| 0.916670024 | 0.708329976 | 0.095569998 |
| 0.666670024 | 0.083329998 | 0.095569998 |
| 0.750000000 | 0.208330005 | 0.095569998 |
| 0.833329976 | 0.333330005 | 0.095569998 |
| 0.916670024 | 0.458330005 | 0.095569998 |
| 0.833329976 | 0.083329998 | 0.095569998 |
| 0.916670024 | 0.208330005 | 0.095569998 |
| 0.000000000 | 0.000000000 | 0.193920001 |
| 0.000000000 | 0.750000000 | 0.193920001 |
| 0.083329998 | 0.875000000 | 0.193920001 |
| 0.166669995 | 0.000000000 | 0.193920001 |
| 0.000000000 | 0.500000000 | 0.193920001 |
| 0.083329998 | 0.625000000 | 0.193920001 |
| 0.166669995 | 0.750000000 | 0.193920001 |
| 0.250000000 | 0.875000000 | 0.193920001 |
| 0.333330005 | 0.000000000 | 0.193920001 |
| 0.000000000 | 0.250000000 | 0.193920001 |
| 0.083329998 | 0.375000000 | 0.193920001 |

| | | |
|---|---|---|
| 0.166669995 | 0.500000000 | 0.193920001 |
| 0.250000000 | 0.625000000 | 0.193920001 |
| 0.333330005 | 0.750000000 | 0.193920001 |
| 0.416669995 | 0.875000000 | 0.193920001 |
| 0.500000000 | 0.000000000 | 0.193920001 |
| 0.083329998 | 0.125000000 | 0.193920001 |
| 0.166669995 | 0.250000000 | 0.193920001 |
| 0.250000000 | 0.375000000 | 0.193920001 |
| 0.333330005 | 0.500000000 | 0.193920001 |
| 0.416669995 | 0.625000000 | 0.193920001 |
| 0.500000000 | 0.750000000 | 0.193920001 |
| 0.583329976 | 0.875000000 | 0.193920001 |
| 0.666670024 | 0.000000000 | 0.193920001 |
| 0.250000000 | 0.125000000 | 0.193920001 |
| 0.333330005 | 0.250000000 | 0.193920001 |
| 0.416669995 | 0.375000000 | 0.193920001 |
| 0.500000000 | 0.500000000 | 0.193920001 |
| 0.583329976 | 0.625000000 | 0.193920001 |
| 0.666670024 | 0.750000000 | 0.193920001 |
| 0.750000000 | 0.875000000 | 0.193920001 |
| 0.833329976 | 0.000000000 | 0.193920001 |
| 0.416669995 | 0.125000000 | 0.193920001 |
| 0.500000000 | 0.250000000 | 0.193920001 |
| 0.583329976 | 0.375000000 | 0.193920001 |
| 0.666670024 | 0.500000000 | 0.193920001 |
| 0.750000000 | 0.625000000 | 0.193920001 |
| 0.833329976 | 0.750000000 | 0.193920001 |
| 0.916670024 | 0.875000000 | 0.193920001 |
| 0.583329976 | 0.125000000 | 0.193920001 |
| 0.666670024 | 0.250000000 | 0.193920001 |
| 0.750000000 | 0.375000000 | 0.193920001 |
| 0.833329976 | 0.500000000 | 0.193920001 |
| 0.916670024 | 0.625000000 | 0.193920001 |
| 0.750000000 | 0.125000000 | 0.193920001 |
| 0.833329976 | 0.250000000 | 0.193920001 |
| 0.916670024 | 0.375000000 | 0.193920001 |
| 0.916670024 | 0.125000000 | 0.193920001 |
| 0.999856412 | 0.914202213 | 0.291615039 |
| 0.999149859 | 0.663793266 | 0.292401761 |
| 0.082602009 | 0.789265335 | 0.291742533 |
| 0.166414529 | 0.914219081 | 0.292303115 |
| 0.999397516 | 0.413782597 | 0.292471379 |
| 0.082516894 | 0.538968742 | 0.292807907 |
| 0.165031135 | 0.664286911 | 0.290575206 |
| 0.249764159 | 0.789737821 | 0.290872395 |
| 0.333452791 | 0.914069414 | 0.292228162 |
| 0.999850988 | 0.163521275 | 0.292159617 |
| 0.083012253 | 0.288451314 | 0.291410983 |
| 0.165616736 | 0.414149880 | 0.290783376 |
| 0.249288887 | 0.539186060 | 0.290494084 |
| 0.333775699 | 0.664083362 | 0.290775448 |
| 0.417116255 | 0.789132059 | 0.291870594 |
| 0.500144005 | 0.913757563 | 0.292139351 |
| 0.083222143 | 0.038968123 | 0.292395592 |
| 0.166506648 | 0.163357615 | 0.292278379 |
| 0.250033051 | 0.287922025 | 0.290923864 |
| 0.333934247 | 0.413471192 | 0.290513545 |
| 0.417157918 | 0.538973331 | 0.291454405 |
| 0.500211179 | 0.663857460 | 0.292155206 |
| 0.583516777 | 0.788486660 | 0.292302191 |

| | | |
|---|---|---|
| 0.666725278 | 0.913456082 | 0.292294890 |
| 0.250035614 | 0.038788944 | 0.292454243 |
| 0.333443522 | 0.163333744 | 0.292274117 |
| 0.416971326 | 0.288302839 | 0.292308509 |
| 0.500098109 | 0.413575560 | 0.292249560 |
| 0.583505809 | 0.538766325 | 0.292292178 |
| 0.666614711 | 0.663415968 | 0.292227030 |
| 0.750001132 | 0.788399339 | 0.292544276 |
| 0.833105445 | 0.913670480 | 0.292145520 |
| 0.416891754 | 0.038820207 | 0.292220086 |
| 0.500156522 | 0.163596407 | 0.292142749 |
| 0.583359599 | 0.288500607 | 0.292292476 |
| 0.666580617 | 0.413609564 | 0.292100132 |
| 0.749953628 | 0.538540721 | 0.292432964 |
| 0.832973897 | 0.663400531 | 0.292267054 |
| 0.916237056 | 0.788442552 | 0.291826546 |
| 0.583497822 | 0.038661074 | 0.292322785 |
| 0.666795433 | 0.163459644 | 0.292074978 |
| 0.750033200 | 0.288480073 | 0.292147845 |
| 0.833209753 | 0.413567275 | 0.292238414 |
| 0.916482270 | 0.538702965 | 0.292514175 |
| 0.749993086 | 0.038537398 | 0.292630672 |
| 0.833368361 | 0.163520977 | 0.292114228 |
| 0.916636884 | 0.288595706 | 0.292324781 |
| 0.916447103 | 0.038684729 | 0.292597026 |
| 0.247471079 | 0.774148285 | 0.418978810 |
| 0.315017223 | 0.735840797 | 0.418225825 |
| 0.311729521 | 0.666290283 | 0.418265909 |
| 0.239754379 | 0.633026958 | 0.419337660 |
| 0.172190860 | 0.672761619 | 0.419352263 |
| 0.175702602 | 0.742284060 | 0.419638574 |
| 0.228431687 | 0.561199069 | 0.419224590 |
| 0.943251729 | 0.860719681 | 0.421737373 |
| 0.211902604 | 0.301228642 | 0.419068813 |
| 0.155643404 | 0.351346076 | 0.418468714 |
| 0.176697016 | 0.418492168 | 0.419018418 |
| 0.254998952 | 0.437584579 | 0.420897722 |
| 0.310972899 | 0.386562228 | 0.421001971 |
| 0.289635330 | 0.319317192 | 0.420163929 |
| 0.279132903 | 0.508551896 | 0.421230584 |
| 0.360389739 | 0.519379497 | 0.421930969 |
| 0.997919917 | 0.830788136 | 0.433949679 |
| 0.427562982 | 0.525811017 | 0.421018630 |
| 0.371143609 | 0.760458589 | 0.417071551 |
| 0.365353256 | 0.638110340 | 0.417886794 |
| 0.115964271 | 0.648150742 | 0.419621378 |
| 0.122532934 | 0.771440268 | 0.420401305 |
| 0.167557269 | 0.547291994 | 0.417693168 |
| 0.892744541 | 0.888348341 | 0.409929961 |
| 0.250765502 | 0.828563690 | 0.419131994 |
| 0.094684377 | 0.338035613 | 0.416976452 |
| 0.131079778 | 0.455914468 | 0.418316841 |
| 0.372044146 | 0.399624258 | 0.421905071 |
| 0.334309667 | 0.280958325 | 0.420001090 |
| 0.996547699 | 0.239481613 | 0.435415626 |
| 0.995318830 | 0.238921478 | 0.405245423 |
| 0.195060819 | 0.248759151 | 0.418314815 |

11. L(C=C)** in Figure 3.

```
CONTCAR
1.0
      17.3481998444         0.0000000000         0.0000000000
       0.0000000000        20.0319004059         0.0000000000
       0.0000000000         0.0000000000        25.0000000000
   Au    C    N    H
  144   16    2   14
Direct
     0.000000000         0.833329976         0.095569998
     0.083329998         0.958329976         0.095569998
     0.000000000         0.583329976         0.095569998
     0.083329998         0.708329976         0.095569998
     0.166669995         0.833329976         0.095569998
     0.250000000         0.958329976         0.095569998
     0.000000000         0.333330005         0.095569998
     0.083329998         0.458330005         0.095569998
     0.166669995         0.583329976         0.095569998
     0.250000000         0.708329976         0.095569998
     0.333330005         0.833329976         0.095569998
     0.416669995         0.958329976         0.095569998
     0.000000000         0.083329998         0.095569998
     0.083329998         0.208330005         0.095569998
     0.166669995         0.333330005         0.095569998
     0.250000000         0.458330005         0.095569998
     0.333330005         0.583329976         0.095569998
     0.416669995         0.708329976         0.095569998
     0.500000000         0.833329976         0.095569998
     0.583329976         0.958329976         0.095569998
     0.166669995         0.083329998         0.095569998
     0.250000000         0.208330005         0.095569998
     0.333330005         0.333330005         0.095569998
     0.416669995         0.458330005         0.095569998
     0.500000000         0.583329976         0.095569998
     0.583329976         0.708329976         0.095569998
     0.666670024         0.833329976         0.095569998
     0.750000000         0.958329976         0.095569998
     0.333330005         0.083329998         0.095569998
     0.416669995         0.208330005         0.095569998
     0.500000000         0.333330005         0.095569998
     0.583329976         0.458330005         0.095569998
     0.666670024         0.583329976         0.095569998
     0.750000000         0.708329976         0.095569998
     0.833329976         0.833329976         0.095569998
     0.916670024         0.958329976         0.095569998
     0.500000000         0.083329998         0.095569998
     0.583329976         0.208330005         0.095569998
     0.666670024         0.333330005         0.095569998
     0.750000000         0.458330005         0.095569998
     0.833329976         0.583329976         0.095569998
     0.916670024         0.708329976         0.095569998
     0.666670024         0.083329998         0.095569998
     0.750000000         0.208330005         0.095569998
     0.833329976         0.333330005         0.095569998
     0.916670024         0.458330005         0.095569998
     0.833329976         0.083329998         0.095569998
     0.916670024         0.208330005         0.095569998
     0.000000000         0.000000000         0.193918005
     0.000000000         0.750000000         0.193918005
     0.083329998         0.875000000         0.193918005
```

| | | |
|---|---|---|
| 0.166669995 | 0.000000000 | 0.193918005 |
| 0.000000000 | 0.500000000 | 0.193918005 |
| 0.083329998 | 0.625000000 | 0.193918005 |
| 0.166669995 | 0.750000000 | 0.193918005 |
| 0.250000000 | 0.875000000 | 0.193918005 |
| 0.333330005 | 0.000000000 | 0.193918005 |
| 0.000000000 | 0.250000000 | 0.193918005 |
| 0.083329998 | 0.375000000 | 0.193918005 |
| 0.166669995 | 0.500000000 | 0.193918005 |
| 0.250000000 | 0.625000000 | 0.193918005 |
| 0.333330005 | 0.750000000 | 0.193918005 |
| 0.416669995 | 0.875000000 | 0.193918005 |
| 0.500000000 | 0.000000000 | 0.193918005 |
| 0.083329998 | 0.125000000 | 0.193918005 |
| 0.166669995 | 0.250000000 | 0.193918005 |
| 0.250000000 | 0.375000000 | 0.193918005 |
| 0.333330005 | 0.500000000 | 0.193918005 |
| 0.416669995 | 0.625000000 | 0.193918005 |
| 0.500000000 | 0.750000000 | 0.193918005 |
| 0.583329976 | 0.875000000 | 0.193918005 |
| 0.666670024 | 0.000000000 | 0.193918005 |
| 0.250000000 | 0.125000000 | 0.193918005 |
| 0.333330005 | 0.250000000 | 0.193918005 |
| 0.416669995 | 0.375000000 | 0.193918005 |
| 0.500000000 | 0.500000000 | 0.193918005 |
| 0.583329976 | 0.625000000 | 0.193918005 |
| 0.666670024 | 0.750000000 | 0.193918005 |
| 0.750000000 | 0.875000000 | 0.193918005 |
| 0.833329976 | 0.000000000 | 0.193918005 |
| 0.416669995 | 0.125000000 | 0.193918005 |
| 0.500000000 | 0.250000000 | 0.193918005 |
| 0.583329976 | 0.375000000 | 0.193918005 |
| 0.666670024 | 0.500000000 | 0.193918005 |
| 0.750000000 | 0.625000000 | 0.193918005 |
| 0.833329976 | 0.750000000 | 0.193918005 |
| 0.916670024 | 0.875000000 | 0.193918005 |
| 0.583329976 | 0.125000000 | 0.193918005 |
| 0.666670024 | 0.250000000 | 0.193918005 |
| 0.750000000 | 0.375000000 | 0.193918005 |
| 0.833329976 | 0.500000000 | 0.193918005 |
| 0.916670024 | 0.625000000 | 0.193918005 |
| 0.750000000 | 0.125000000 | 0.193918005 |
| 0.833329976 | 0.250000000 | 0.193918005 |
| 0.916670024 | 0.375000000 | 0.193918005 |
| 0.916670024 | 0.125000000 | 0.193918005 |
| 0.999897480 | 0.913612127 | 0.291693896 |
| 0.999568641 | 0.663558662 | 0.292240560 |
| 0.082852826 | 0.788652241 | 0.292576939 |
| 0.166463718 | 0.913948238 | 0.292285144 |
| 0.999522746 | 0.413838685 | 0.292402327 |
| 0.082962237 | 0.539076149 | 0.292584598 |
| 0.165767550 | 0.663920462 | 0.292429537 |
| 0.249131173 | 0.789396942 | 0.291837424 |
| 0.333114266 | 0.914337814 | 0.291972309 |
| 0.999994636 | 0.163725749 | 0.292162746 |
| 0.083120622 | 0.288718283 | 0.292008191 |
| 0.166033477 | 0.414260089 | 0.290937752 |
| 0.249691546 | 0.539251149 | 0.290784329 |
| 0.332109451 | 0.664658129 | 0.289935201 |
| 0.417019993 | 0.789929748 | 0.290795714 |

| | | |
|---|---|---|
| 0.500302792 | 0.914364457 | 0.292038709 |
| 0.083103202 | 0.038755868 | 0.292210758 |
| 0.166378930 | 0.163608760 | 0.292318702 |
| 0.249878362 | 0.288220704 | 0.291051358 |
| 0.334597886 | 0.413650542 | 0.290201962 |
| 0.417201698 | 0.539203763 | 0.291126996 |
| 0.500532627 | 0.663930655 | 0.291384757 |
| 0.583995700 | 0.788942277 | 0.291933328 |
| 0.666686833 | 0.913724899 | 0.292285085 |
| 0.249809489 | 0.039110281 | 0.292166144 |
| 0.333327323 | 0.163751423 | 0.292194664 |
| 0.417058378 | 0.288475394 | 0.292252958 |
| 0.500569701 | 0.413891107 | 0.292764038 |
| 0.583382726 | 0.538895786 | 0.291905284 |
| 0.666977823 | 0.663771152 | 0.292265147 |
| 0.750243664 | 0.788405836 | 0.292922080 |
| 0.832815230 | 0.913721740 | 0.291127533 |
| 0.416658103 | 0.039117169 | 0.292223364 |
| 0.500033259 | 0.163807571 | 0.292167455 |
| 0.583334029 | 0.288704306 | 0.292549223 |
| 0.666727901 | 0.413558125 | 0.292124540 |
| 0.750273407 | 0.538903713 | 0.291465700 |
| 0.833297908 | 0.663462043 | 0.292574763 |
| 0.916390479 | 0.788356066 | 0.292354554 |
| 0.583509862 | 0.039093759 | 0.292188913 |
| 0.666761339 | 0.163780138 | 0.292089105 |
| 0.750073493 | 0.288559854 | 0.292294532 |
| 0.833074987 | 0.413590103 | 0.292804301 |
| 0.916543603 | 0.538573146 | 0.292663723 |
| 0.750004768 | 0.038858511 | 0.292390227 |
| 0.833400190 | 0.163628384 | 0.292135298 |
| 0.916707814 | 0.288674921 | 0.292326063 |
| 0.916651785 | 0.038856819 | 0.292319506 |
| 0.455345839 | 0.756964207 | 0.417249203 |
| 0.492166191 | 0.694554806 | 0.418047845 |
| 0.449245214 | 0.635791779 | 0.418877512 |
| 0.367766827 | 0.637626469 | 0.418691039 |
| 0.331698745 | 0.700802147 | 0.417296261 |
| 0.374856085 | 0.759749353 | 0.416896611 |
| 0.319819331 | 0.577874720 | 0.419159293 |
| 0.888475120 | 0.901832759 | 0.420541167 |
| 0.211364418 | 0.333389878 | 0.418584675 |
| 0.173720300 | 0.395382434 | 0.417723328 |
| 0.215927348 | 0.454587787 | 0.418186873 |
| 0.297365069 | 0.453689963 | 0.419278771 |
| 0.334169865 | 0.390813887 | 0.419131964 |
| 0.291835368 | 0.331446052 | 0.419160157 |
| 0.344909191 | 0.513698399 | 0.419524461 |
| 0.672616839 | 0.514637887 | 0.422341436 |
| 0.907031298 | 0.956815958 | 0.428290099 |
| 0.615361333 | 0.540848196 | 0.434319705 |
| 0.554993749 | 0.691505075 | 0.418431878 |
| 0.480099827 | 0.588397622 | 0.420186937 |
| 0.268783301 | 0.703470945 | 0.416792631 |
| 0.345419526 | 0.807906210 | 0.416056961 |
| 0.257706821 | 0.587587833 | 0.418712497 |
| 0.871384084 | 0.851020098 | 0.412983537 |
| 0.489266247 | 0.802825987 | 0.416852951 |
| 0.110853434 | 0.397418708 | 0.416820288 |
| 0.185066521 | 0.502066553 | 0.417433888 |

```
0.397131562  0.388861239  0.419373065
0.321938455  0.283597171  0.419185609
0.407064646  0.504231870  0.419877410
0.725445032  0.490413696  0.410652131
0.177979708  0.287218660  0.418388635
```

12. L(C□C) in Figure 3.

```
CONTCAR
1.0
      17.3481998444         0.0000000000         0.0000000000
       0.0000000000        20.0319004059         0.0000000000
       0.0000000000         0.0000000000        25.0000000000
   Au    C    N    H
  144   16    2   14
Direct
     0.000000000         0.833329976         0.095569998
     0.083329998         0.958329976         0.095569998
     0.000000000         0.583329976         0.095569998
     0.083329998         0.708329976         0.095569998
     0.166669995         0.833329976         0.095569998
     0.250000000         0.958329976         0.095569998
     0.000000000         0.333330005         0.095569998
     0.083329998         0.458330005         0.095569998
     0.166669995         0.583329976         0.095569998
     0.250000000         0.708329976         0.095569998
     0.333330005         0.833329976         0.095569998
     0.416669995         0.958329976         0.095569998
     0.000000000         0.083329998         0.095569998
     0.083329998         0.208330005         0.095569998
     0.166669995         0.333330005         0.095569998
     0.250000000         0.458330005         0.095569998
     0.333330005         0.583329976         0.095569998
     0.416669995         0.708329976         0.095569998
     0.500000000         0.833329976         0.095569998
     0.583329976         0.958329976         0.095569998
     0.166669995         0.083329998         0.095569998
     0.250000000         0.208330005         0.095569998
     0.333330005         0.333330005         0.095569998
     0.416669995         0.458330005         0.095569998
     0.500000000         0.583329976         0.095569998
     0.583329976         0.708329976         0.095569998
     0.666670024         0.833329976         0.095569998
     0.750000000         0.958329976         0.095569998
     0.333330005         0.083329998         0.095569998
     0.416669995         0.208330005         0.095569998
     0.500000000         0.333330005         0.095569998
     0.583329976         0.458330005         0.095569998
     0.666670024         0.583329976         0.095569998
     0.750000000         0.708329976         0.095569998
     0.833329976         0.833329976         0.095569998
     0.916670024         0.958329976         0.095569998
     0.500000000         0.083329998         0.095569998
     0.583329976         0.208330005         0.095569998
     0.666670024         0.333330005         0.095569998
     0.750000000         0.458330005         0.095569998
     0.833329976         0.583329976         0.095569998
     0.916670024         0.708329976         0.095569998
     0.666670024         0.083329998         0.095569998
```

| | | |
|---|---|---|
| 0.750000000 | 0.208330005 | 0.095569998 |
| 0.833329976 | 0.333330005 | 0.095569998 |
| 0.916670024 | 0.458330005 | 0.095569998 |
| 0.833329976 | 0.083329998 | 0.095569998 |
| 0.916670024 | 0.208330005 | 0.095569998 |
| 0.000000000 | 0.000000000 | 0.193918005 |
| 0.000000000 | 0.750000000 | 0.193918005 |
| 0.083329998 | 0.875000000 | 0.193918005 |
| 0.166669995 | 0.000000000 | 0.193918005 |
| 0.000000000 | 0.500000000 | 0.193918005 |
| 0.083329998 | 0.625000000 | 0.193918005 |
| 0.166669995 | 0.750000000 | 0.193918005 |
| 0.250000000 | 0.875000000 | 0.193918005 |
| 0.333330005 | 0.000000000 | 0.193918005 |
| 0.000000000 | 0.250000000 | 0.193918005 |
| 0.083329998 | 0.375000000 | 0.193918005 |
| 0.166669995 | 0.500000000 | 0.193918005 |
| 0.250000000 | 0.625000000 | 0.193918005 |
| 0.333330005 | 0.750000000 | 0.193918005 |
| 0.416669995 | 0.875000000 | 0.193918005 |
| 0.500000000 | 0.000000000 | 0.193918005 |
| 0.083329998 | 0.125000000 | 0.193918005 |
| 0.166669995 | 0.250000000 | 0.193918005 |
| 0.250000000 | 0.375000000 | 0.193918005 |
| 0.333330005 | 0.500000000 | 0.193918005 |
| 0.416669995 | 0.625000000 | 0.193918005 |
| 0.500000000 | 0.750000000 | 0.193918005 |
| 0.583329976 | 0.875000000 | 0.193918005 |
| 0.666670024 | 0.000000000 | 0.193918005 |
| 0.250000000 | 0.125000000 | 0.193918005 |
| 0.333330005 | 0.250000000 | 0.193918005 |
| 0.416669995 | 0.375000000 | 0.193918005 |
| 0.500000000 | 0.500000000 | 0.193918005 |
| 0.583329976 | 0.625000000 | 0.193918005 |
| 0.666670024 | 0.750000000 | 0.193918005 |
| 0.750000000 | 0.875000000 | 0.193918005 |
| 0.833329976 | 0.000000000 | 0.193918005 |
| 0.416669995 | 0.125000000 | 0.193918005 |
| 0.500000000 | 0.250000000 | 0.193918005 |
| 0.583329976 | 0.375000000 | 0.193918005 |
| 0.666670024 | 0.500000000 | 0.193918005 |
| 0.750000000 | 0.625000000 | 0.193918005 |
| 0.833329976 | 0.750000000 | 0.193918005 |
| 0.916670024 | 0.875000000 | 0.193918005 |
| 0.583329976 | 0.125000000 | 0.193918005 |
| 0.666670024 | 0.250000000 | 0.193918005 |
| 0.750000000 | 0.375000000 | 0.193918005 |
| 0.833329976 | 0.500000000 | 0.193918005 |
| 0.916670024 | 0.625000000 | 0.193918005 |
| 0.750000000 | 0.125000000 | 0.193918005 |
| 0.833329976 | 0.250000000 | 0.193918005 |
| 0.916670024 | 0.375000000 | 0.193918005 |
| 0.916670024 | 0.125000000 | 0.193918005 |
| 0.999916852 | 0.914317966 | 0.291716188 |
| 0.999876499 | 0.664234757 | 0.292061508 |
| 0.083065651 | 0.789343119 | 0.292528480 |
| 0.166488498 | 0.914573669 | 0.292107195 |
| 0.999699533 | 0.414423376 | 0.292277992 |
| 0.083105788 | 0.539642096 | 0.292436898 |
| 0.166258991 | 0.664647222 | 0.292468876 |

| | | |
|---|---|---|
| 0.249384791 | 0.789934456 | 0.291918188 |
| 0.333221644 | 0.914726555 | 0.292012721 |
| 0.999898911 | 0.164407969 | 0.292098135 |
| 0.083109848 | 0.289328545 | 0.291992068 |
| 0.166219383 | 0.414846420 | 0.291016132 |
| 0.249760568 | 0.539904535 | 0.291290194 |
| 0.332662970 | 0.665077031 | 0.290059090 |
| 0.417153627 | 0.790353417 | 0.290806800 |
| 0.500397027 | 0.914775193 | 0.292054981 |
| 0.083084837 | 0.039381795 | 0.292109221 |
| 0.166347370 | 0.164141893 | 0.292258203 |
| 0.249744177 | 0.288701087 | 0.291139543 |
| 0.334342957 | 0.414200068 | 0.290102988 |
| 0.417236269 | 0.539448798 | 0.291571110 |
| 0.500844717 | 0.664288342 | 0.291240156 |
| 0.584119141 | 0.789440930 | 0.291990936 |
| 0.666768312 | 0.914256036 | 0.292441636 |
| 0.249828011 | 0.039551724 | 0.292058080 |
| 0.333291948 | 0.164197803 | 0.292212814 |
| 0.416895568 | 0.288999885 | 0.292159438 |
| 0.500489235 | 0.414314270 | 0.292645842 |
| 0.583514929 | 0.539290726 | 0.291961730 |
| 0.667210340 | 0.664243996 | 0.292292774 |
| 0.750305533 | 0.789073646 | 0.292894930 |
| 0.832886875 | 0.914395154 | 0.291104823 |
| 0.416690320 | 0.039481830 | 0.292127579 |
| 0.499912381 | 0.164346144 | 0.292160660 |
| 0.583267272 | 0.289262205 | 0.292557329 |
| 0.666790962 | 0.414100349 | 0.291716576 |
| 0.750428975 | 0.539458454 | 0.291650534 |
| 0.833503783 | 0.664232016 | 0.292485744 |
| 0.916559875 | 0.789141059 | 0.292358369 |
| 0.583506107 | 0.039564941 | 0.292079836 |
| 0.666627586 | 0.164428443 | 0.291955531 |
| 0.750013947 | 0.289162487 | 0.292496085 |
| 0.833128154 | 0.414128751 | 0.292723417 |
| 0.916642189 | 0.539163649 | 0.292399168 |
| 0.749928772 | 0.039438389 | 0.292235523 |
| 0.833299041 | 0.164303541 | 0.292040259 |
| 0.916661739 | 0.289359242 | 0.292409241 |
| 0.916568875 | 0.039463550 | 0.292151004 |
| 0.451146245 | 0.764521420 | 0.418672740 |
| 0.498078376 | 0.707594514 | 0.419443488 |
| 0.465481669 | 0.643961430 | 0.419961542 |
| 0.384400368 | 0.636369407 | 0.419699669 |
| 0.337477267 | 0.694043577 | 0.418333501 |
| 0.370897025 | 0.757425785 | 0.418105513 |
| 0.350762904 | 0.571950018 | 0.419772238 |
| 0.881003320 | 0.901975453 | 0.421812385 |
| 0.222019583 | 0.323338419 | 0.419904292 |
| 0.174754411 | 0.380056471 | 0.419413894 |
| 0.207128748 | 0.443769991 | 0.419509441 |
| 0.288103223 | 0.451867104 | 0.420086294 |
| 0.335276157 | 0.394314021 | 0.419771463 |
| 0.302185446 | 0.330815226 | 0.419981062 |
| 0.321426421 | 0.516394436 | 0.419794768 |
| 0.677164733 | 0.504193902 | 0.421678752 |
| 0.914406657 | 0.951360047 | 0.429762393 |
| 0.630292475 | 0.542860389 | 0.433747739 |
| 0.560737133 | 0.712557256 | 0.419647664 |

| | | |
|---|---|---|
| 0.502515912 | 0.599993527 | 0.421183676 |
| 0.274942696 | 0.688423932 | 0.417731792 |
| 0.334009498 | 0.801549196 | 0.417047799 |
| 0.850305974 | 0.856245697 | 0.414171666 |
| 0.916638911 | 0.254680783 | 0.622365713 |
| 0.477016479 | 0.814179420 | 0.418324828 |
| 0.112167045 | 0.374537259 | 0.418761104 |
| 0.170397952 | 0.487963080 | 0.419006795 |
| 0.397775382 | 0.400242716 | 0.419636250 |
| 0.339333087 | 0.286839932 | 0.419654816 |
| 0.918229938 | 0.217304513 | 0.620256662 |
| 0.720341086 | 0.468414068 | 0.409945637 |
| 0.196371123 | 0.273598403 | 0.419896007 |
